# A New Pose Estimation Algorithm Using a Perspective-Ray-Based Scaled Orthographic Projection with Iteration

**DOI:** 10.1371/journal.pone.0134029

**Published:** 2015-07-21

**Authors:** Pengfei Sun, Changku Sun, Wenqiang Li, Peng Wang

**Affiliations:** 1 State Key Laboratory of Precision Measuring Technology and Instruments, Tianjin University, Tianjin, China; 2 Science and Technology on Electro-optic Control Laboratory, Luoyang Institute of Electro-optic Equipment, Luoyang, China; Glasgow University, UNITED KINGDOM

## Abstract

Pose estimation aims at measuring the position and orientation of a calibrated camera using known image features. The pinhole model is the dominant camera model in this field. However, the imaging precision of this model is not accurate enough for an advanced pose estimation algorithm. In this paper, a new camera model, called incident ray tracking model, is introduced. More importantly, an advanced pose estimation algorithm based on the perspective ray in the new camera model, is proposed. The perspective ray, determined by two positioning points, is an abstract mathematical equivalent of the incident ray. In the proposed pose estimation algorithm, called perspective-ray-based scaled orthographic projection with iteration (PRSOI), an approximate ray-based projection is calculated by a linear system and refined by iteration. Experiments on the PRSOI have been conducted, and the results demonstrate that it is of high accuracy in the six degrees of freedom (DOF) motion. And it outperforms three other state-of-the-art algorithms in terms of accuracy during the contrast experiment.

## Introduction

Estimating the pose of a calibrated camera has lots of applications in augmented reality, air refueling, and unmanned aerial vehicle (UAV) navigation [1–3]. The augmented reality often operates on the basis of prior knowledge of the environment, which limits range and accuracy of registration. Pose estimation attempts to locate 3D features in the feature map, and provides registration when the reference map is in the sensing range [4]. In air refueling, a single monocular camera is mounted on the receiver aircraft while the probe and drogue is mounted on the tanker aircraft. Pose estimation algorithm is proposed for the purpose of tracking the drogue during the capture stage of autonomous aerial refueling [5]. In UAV navigation, pose estimation is employed in the formation flying of UAVs. To guarantee the relative positions of these UAVs, the IR-LEDs on the leader UAV is captured by the IR-camera on the follower UAV and the detected features are transmitted to the pose estimation algorithm [6].

Pose estimation, also known in the literature as the Perspective-*n*-Point (P*n*P) problem, measures the position and orientation of a calibrated camera with known image features [7]. The features available to solve the P*n*P problem are usually given in the form of a set of point correspondences, each constituting a space point expressed in object coordinates and its image projection expressed in image coordinates. In the past few decades, a huge amount of work has been done to address the problem. Various solutions to the P*n*P problem, including the EP*n*P [8], the DLS [9], the RP*n*P [10], the ASP*n*P [11], the LHM [12], etc., are developed. To the best of our knowledge, these P*n*P solutions can show high accuracy only when dealing with dozens or even hundreds of point correspondences. Unfortunately, considering the terrible environment in pose estimation applications, it is hard to offer too many stable and distinguishable point correspondences. Although the DLS is applicable to situations of *n*≤7, the moving range of the target object is extremely limited [9]. The P*n*P solutions, especially the P4P solutions, have been in great demand in recent years. The P4P solutions can be classified into two types: model-based solutions, which depend on the approximation of a camera model, and geometric configuration solutions that handle the relationship between image space and object space with geometrical characteristic such as distance, angle, parallel, vertical, etc.. POSIT is a popular solution to the non-coplanar P4P problem and is one of the representative solutions in the first category [13]. Scaled orthographic projection is employed in the algorithm, and the rotation matrix and translation vector of a calibrated camera is obtained through the projection. Iteration is also introduced to refresh the old image coordinates of feature points, and then repeat the previous steps. The iteration does not stop until the output has satisfied the preset accuracy or the algorithm is circulated for preset times. For the stability and high accuracy, the POSIT is continuously introduced into applications in complex interference environment [14–19]. The latter solutions take advantage of the geometric configuration of the special feature points. The geometric configuration of the P4P problem is a core research. Liu. M. L. et al. [20] made full use of the geometric configuration of the four non-coplanar feature points, including the angle between two perspective lines, the mixed product among the perspective lines, the segments in object space, etc.. The follow-up researches did not surpass the category of the geometric configuration by Liu. M. L.. Z. Y. Hu et al. [21] mathematically analyzed the geometric configuration of non-coplanar P4P problem. They parameterized the relationship between the numbers of possible solutions and the numbers of geometric configuration. Wu PC et al. [22] focused on the plausible pose, and proposed an analytical motion model to interpret, or even eliminate, the geometric illusion. Yang Guo [23] researched the coplanar P4P problem. By converting perspective transformation to affine transformation and using invariance to 3D affine transformation, it is found that the upper bound of the coplanar P4P problem is two. A technique based on a singular value decomposition (SVD) is also proposed for the coplanar P4P problem by Yang Guo, unverified by any real test. To improve estimation accuracy, Long Li et al. [24] introduced Frobenius norm into the determinant of rotation matrix, instead of the SVD-based method. Unfortunately, the proposed method did not contribute to accuracy and noise resistance, it only reduced the runtime. Bujnak M. et al. [25] and Kuang Y. et al. [26] focused on the recovery of the unknown focal length from the P4P solutions, and were not interested in the accuracy of the P4P solutions. From the studies in [13–26], it can be concluded that the research concerning accuracy improvement of the P4P solutions is slow and unattractive.

To sum up, the camera model of the above solutions is a pinhole camera, in which all the incident rays are projected directly onto the detector plane through a single point, called the effective pinhole of the camera model [27]. In practice, the incident rays are deviated on account of the compound lenses. The P4P solutions are negatively influenced by the imprecise camera model. There are still other expressions proposed to describe the camera model [28–31]. By using lens geometry model, the geometric relationship between images and objects is established via Snell’s Law and skew ray tracking in [28] and [29]. The camera model is represented by a matrix equation that relates the parameters of the image plane with the incident ray. But it is complex because each incident ray is represented by a set of six pose parameters. In a general imaging model, the cameral is regarded as “black box” [30, 31]. A set of virtual sensing elements called “raxel” is used to describe a linear mapping from incident rays to the image plane. The “raxel” is composed of three parameters: an image projection, the yaw and pitch directions of the projective ray. The calibration of “raxel” is tedious and entirely depends on the accuracy of rotation stage. In this paper, an incident ray tracking model (IRT) is proposed, where two reference planes are regarded as camera model parameters. Through analyzing the geometric properties of the proposed model, the incident ray is mathematically summarized as a perspective ray which is positioned by two points respectively located in the two reference planes. Considering the excellent scaled orthographic projection, a perspective-ray-based scaled orthographic projection is employed in this paper. The projection formulates a linear system which calculates the approximation of object pose, and iteration loops are also introduced to obtain a more accurate approximation. The camera calibration based on the incident ray tracking model (IRT) and the perspective-ray-based scaled orthographic projection with iteration (PRSOI) for pose estimation will be described in detail in the following sections.

## Incident Ray Tracking Model

### Incident ray formulation

**Fig 1:**
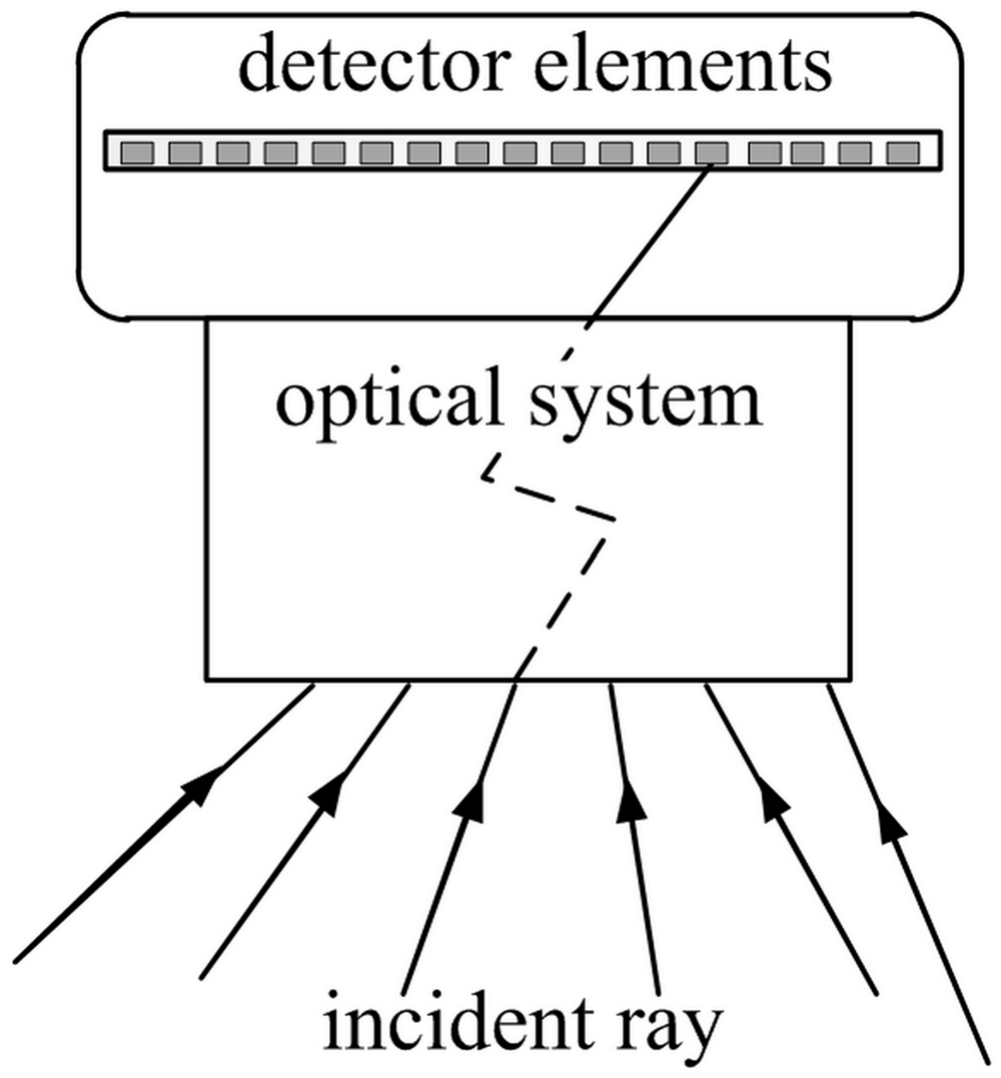
An incident ray passing through an imaging system which is absorbed by detector elements (pixel).

Fig 1 shows the imaging system, helpful to formulate the mathematical model of imaging sensors. Irrespective of its specific design, the purpose of an imaging system is to map incident rays from the scene onto pixels on the detector.

Each pixel in Fig 1 collects energy from the incident ray in the optical system that has a non-zero aperture size. However, the incident ray can be represented by a perspective ray when studying the geometric properties of the imaging system. As shown in Fig 1, the system maps the incident ray to the pixel. Because the path that incident ray traverses from scene to the pixel can be arbitrarily complex, the incident ray should be replaced by an abstract mathematical equivalent that is referred to as a perspective ray l (I, P^*m*^, P^*n*^). The IRT is composed of the incident rays, in the field of view. In the following section, the parameters of the IRT will be introduced.

### Parameters of camera model

**Fig 2:**
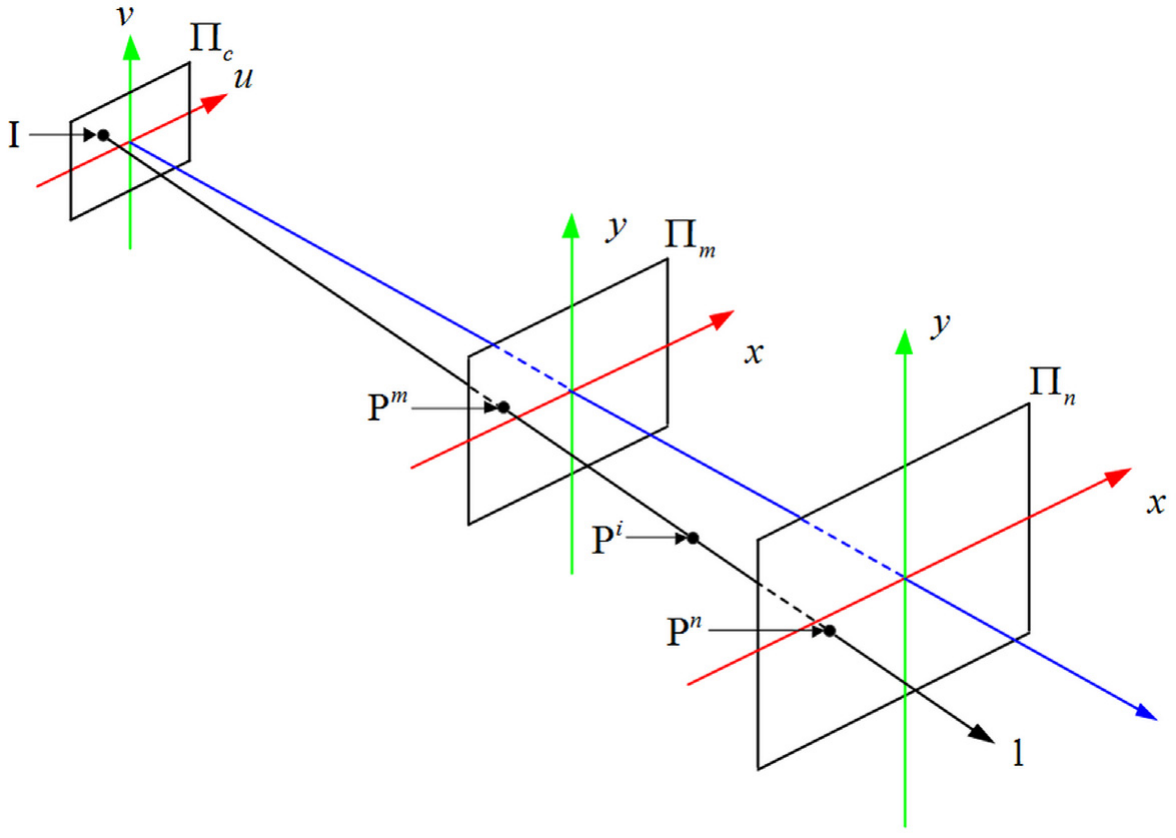
Geometrization of the IRT.

If the radiometric response function of each perspective ray is computable, one can linearize the radiometric response with respect to the image plane. In our context of camera model, the ray to image mapping may be parameterized as Fig 2.

A point P^*i*^(*x*,*y*,*t*) imaged at (*x*,*y*) at depth *t* is imaged along a perspective ray l (*t* is the vertical distance from P^*i*^ to the Π_*n*_). It will be more convenient to represent the model if the perspective rays, such as l, are arranged on two planes called reference planes, such as Π_*m*_ and Π_*n*_. Each perspective ray will intersect the two reference planes respectively at only one point, namely P^*m*^ and P^*n*^. The reference planes could be written as a function:

$$\{\begin{array}{c} \Pi_{m}(x,y)=\left\{{\text{P}}^{m}\right\}\\ \Pi_{n}(x,y)=\left\{{\text{P}}^{n}\right\} \end{array}$$ (1)

A perspective ray could be determined uniquely through the reference planes Π_*m*_ and Π_*n*_, and the IRT is parameterized by the two reference planes.

### Computing parameters

**Fig 3:**
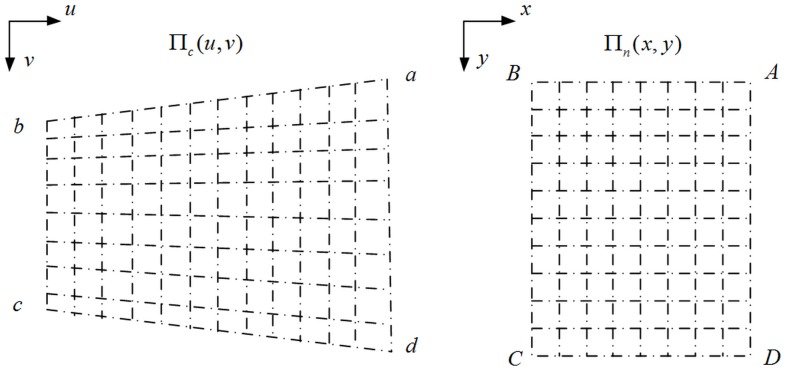
A mapping between reference plane and image plane. The intersection points of dotted lines in plane ABCD corresponds to the ones in plane abcd.

The parameters used to specify the IRT are derived from the reference planes. The perspective ray passes through the two reference planes Π_*m*_(*x*, *y*) and Π_*n*_(*x*, *y*), and intersects the image plane Π_*c*_(*u*, *v*) at point I(*u*,*v*). Ignoring the position of the planes, the mapping from Π_*n*_(*x*, *y*) to Π_*c*_(*u*, *v*) is represented as Fig 3.

There is a one-to-one mapping between the image plane and the reference plane. As the two planes are both represented by a set of points, the mapping is recasted as the following equation:

$$\left\{ \begin{array}{l} x=\sum_{i=0}^{n}\sum_{j=0}^{n-i}C_{ij}u_{}^{i}v_{}^{j}\\ y=\sum_{i=0}^{n}\sum_{j=0}^{n-i}D_{ij}u_{}^{i}v_{}^{j} \end{array}\right.$$ (2)

where (*C*_*ij*_,*D*_*ij*_) are the mapping parameters, *n* is the order of the mapping, (*x*,*y*) is the space coordinates of the points in the plane ABCD while (*u*,*v*) is the image coordinates of them in the plane abcd.

The (*C*_*ij*_,*D*_*ij*_) are obtained using Levenberg-Marquard method [32]. The reference plane Π_*m*_(*x*, *y*) is represented by rational functions $g_{x}^{m}(u,v)$ and $g_{y}^{m}(u,v)$, consisting of $C_{ij}^{m}$ and $D_{ij}^{m}$. *m* can be replaced by *n*.

## Pose Estimation Based on Perspective Ray

### Object pose formulation

**Fig 4:**
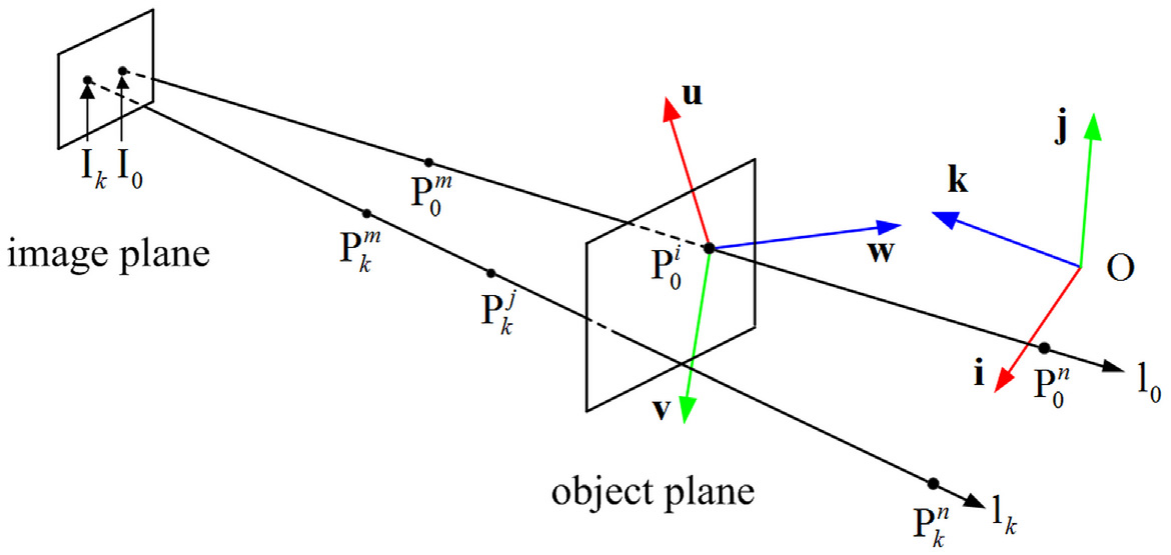
The perspective rays used for object pose.

Considering the geometrical features of the perspective ray ${\text{l}}_{k}({\text{I}}_{k},{\text{P}}_{k}^{m},{\text{P}}_{k}^{n})$, which is described in Fig 4. The points ${\text{P}}_{0}^{i}$ and ${\text{P}}_{k}^{j}$ are located on the object. ${\text{P}}_{0}^{i}\text{-}\text{uvw}$ is the object coordinate system, and O-**ijk** is the reference plane coordinate system.

Pose estimation in this paper aims to compute the rotation matrix and translation vector of the object. The purpose of the rotation matrix is to transform the object coordinates such as $\vec{{\text{P}}_{0}^{i}{\text{P}}_{k}^{j}}$ into coordinates defined in the reference plane coordinate system such as $\vec{{\text{P}}_{0}^{n}{\text{P}}_{k}^{n}}$ (*n* represents a point located on the plane Π_*n*_). The dot product $\vec{{\text{P}}_{0}^{i}{\text{P}}_{k}^{j}}•i$ between the vector $\vec{{\text{P}}_{0}^{i}{\text{P}}_{k}^{j}}$ and the first row of the matrix correctly provides the projection of this vector on the unit vector **i** of the reference plane coordinate system. The rotation matrix can therefore be written as:

$$R=\left[\begin{array}{ccc} i_{u} & i_{v} & i_{w}\\ j_{u} & j_{v} & j_{w}\\ k_{u} & k_{v} & k_{w} \end{array}\right]$$ (3)

where *i*_*u*_, *i*_*v*_, *i*_*w*_ are the coordinates of **i** in the object coordinate system. To compute the rotation matrix, it is only needed to compute **i** and **j** in the object coordinate system. The vector **k** is then obtained by the cross-product **i × j**.

The translation vector, **T**, is the vector $\vec{{\text{OP}}_{0}^{i}}$. The point ${\text{P}}_{0}^{i}$ is determined by the perspective ray l_*0*_ which can be expressed as:

$$\left\{ \begin{array}{l} f_{x}^{0}(z)=g_{x}^{n}(u_{0},v_{0})+(g_{x}^{m}(u_{0},v_{0})-g_{x}^{n}(u_{0},v_{0}))(z-z_{}^{n})/\left(z_{}^{m}-z_{}^{n}\right)\\ f_{y}^{0}(z)=g_{y}^{n}(u_{0},v_{0})+(g_{y}^{m}(u_{0},v_{0})-g_{y}^{n}(u_{0},v_{0}))(z-z_{}^{n})/\left(z_{}^{m}-z_{}^{n}\right) \end{array}\right.$$ (4)

where *z* ^*m*^ and *z* ^*n*^ are respectively the z coordinate of the planes Π_*m*_ and Π_*n*_. From Eq (4), the vector $\vec{{\text{OP}}_{0}^{i}}$ could be expressed as:

$$\vec{{\text{OP}}_{0}^{i}}=(f_{x}^{0}(z_{}^{i})-g_{x}^{n}(0,0),f_{y}^{0}(z_{}^{i})-g_{y}^{n}(0,0),z^{i}-z^{n})$$ (5)

where *z* ^*i*^ is the z coordinate of the plane Π_*i*_. Therefore to compute the object translation, only the *z* coordinate needs computing. Thus the object pose is fully defined once the unknowns **i**, **j** and *z* are found.

### Projection on the perspective ray

**Fig 5:**
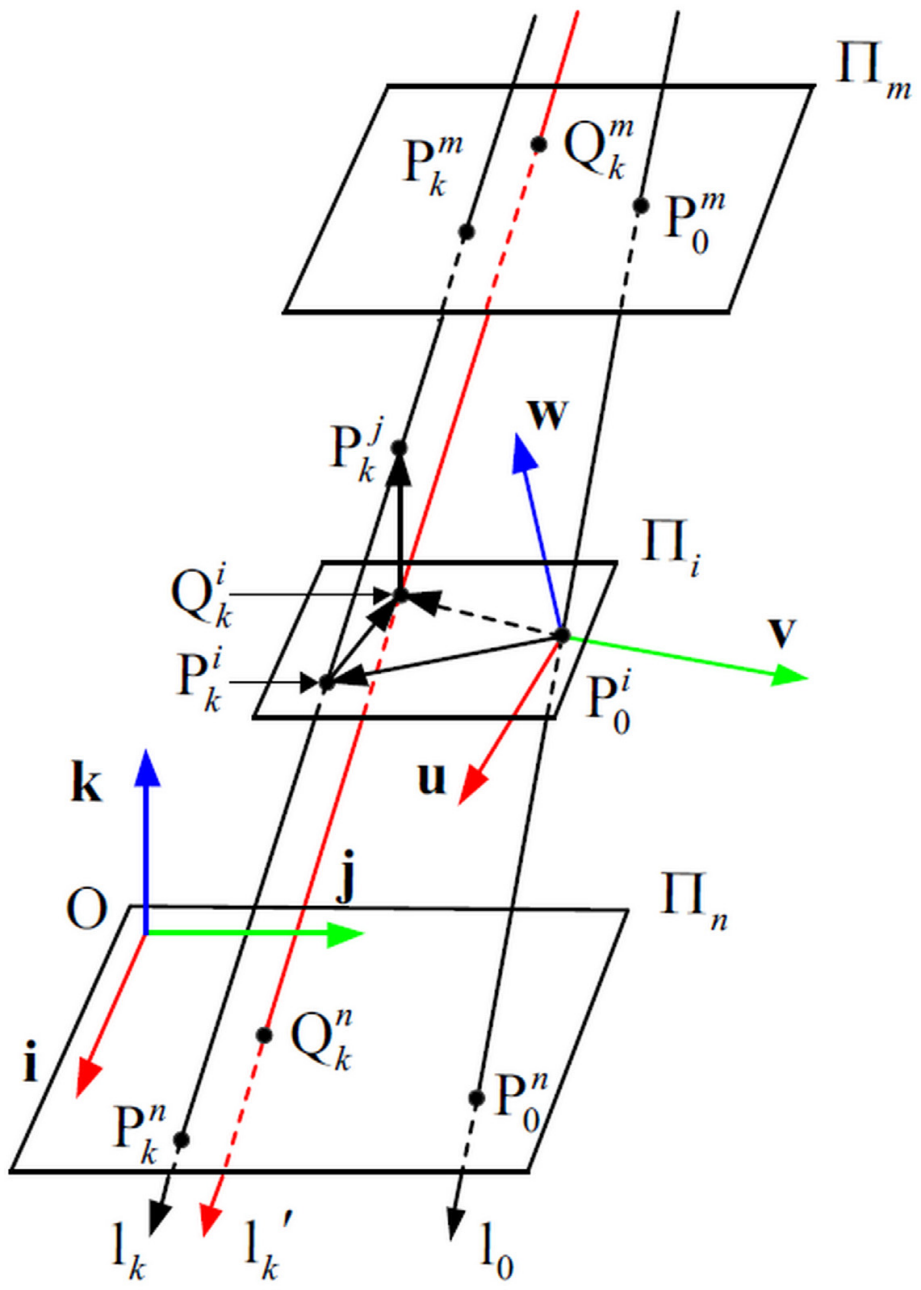
An imaging model of the perspective rays. It includes a perspective projection and a scaled orthographic projection.

The image point corresponding to the feature point, which projects on the perspective ray, is shown in Fig 5. Only two feature points ${\text{P}}_{0}^{i}$ and ${\text{P}}_{k}^{j}$ appeared in the projection. The perspective rays l_*0*_ and l_*k*_ are respectively in correspondence with the feature points ${\text{P}}_{0}^{i}$ and ${\text{P}}_{k}^{j}$, and are computed by the calibration parameters. The object coordinate system is centered at ${\text{P}}_{0}^{i}$, and the coordinate of ${\text{P}}_{k}^{j}$ relative to ${\text{P}}_{0}^{i}$ is known. The point ${\text{P}}_{0}^{i}$ locates on the plane Π_*i*_ which parallels to the planes Π_*m*_ and Π_*n*_.

Scaled orthographic projection is an approximation to the perspective projection. It is assumed that the depths of different points can all be set as the same depth *z* ^*i*^. The geometric construction to obtain the perspective ray l_*k*_ of ${\text{P}}_{k}^{j}$ in a perspective projection and the perspective ray 1_*k*_′ of ${\text{P}}_{k}^{j}$ in a scaled orthographic projection is shown in Fig 5. The point ${\text{P}}_{k}^{j}$ is projected on the plane Π_*i*_ at ${\text{Q}}_{k}^{i}$ by a scaled orthographic projection.

### Formulations of projections

#### Formulations of perspective projection

**Fig 6:**
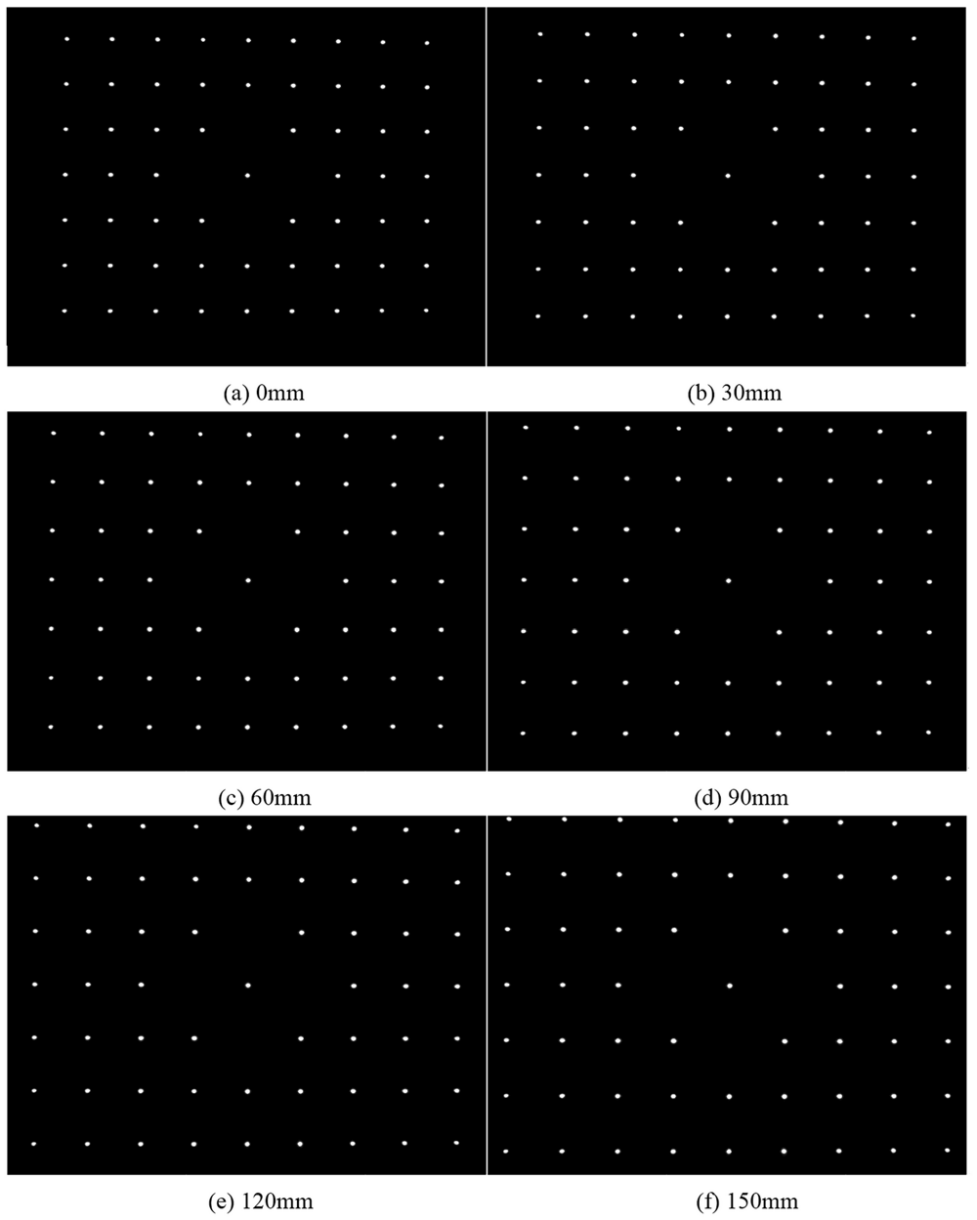
Captured images at six positions.

Now consider the equations that characterize a perspective projection and relate the unknown row vectors **i** and **j** of the rotation matrix and the unknown *z* ^*i*^ coordinate of the translation vector to the known coordinates of the vector $\vec{{\text{P}}_{0}^{i}{\text{P}}_{k}^{j}}$ in the object coordinate system, and to the known coordinates of ${\text{P}}_{0}^{n}$. In Fig 6, the perspective ray l_*k*_ intersects the plane Π_*i*_ in ${\text{P}}_{k}^{i}$, and ${\text{P}}_{k}^{j}$ projects on the plane Π_*i*_ at ${\text{Q}}_{k}^{i}$. The vector $\vec{{\text{P}}_{0}^{i}{\text{P}}_{k}^{j}}$ is the sum of three vectors:

$$\vec{{\text{P}}_{0}^{i}{\text{P}}_{k}^{j}}=\vec{{\text{P}}_{0}^{i}{\text{P}}_{k}^{i}}+\vec{{\text{P}}_{k}^{i}{\text{Q}}_{k}^{i}}+\vec{{\text{Q}}_{k}^{i}{\text{P}}_{k}^{j}}$$ (6)

The vector $\vec{{\text{P}}_{0}^{i}{\text{P}}_{k}^{i}}$ is constrained by two perspective rays l_*0*_ and l_*k*_. It can be expressed as:

$$\vec{{\text{P}}_{0}^{i}{\text{P}}_{k}^{i}}=(f_{x}^{k}(z^{i})-f_{x}^{0}(z^{i}),f_{y}^{k}(z^{i})-f_{y}^{0}(z^{i}),0)$$ (7)

where $\left(f_{x}^{0},f_{y}^{0}\right)$ and $\left(f_{x}^{k},f_{y}^{k}\right)$ are the functions of l_*0*_ and l_*k*_. The vector $\vec{{\text{P}}_{k}^{i}{\text{Q}}_{k}^{i}}$ is also constrained by l_*k*_ and ${\text{l}}_{k}{}^{\prime }$. For the z coordinate of ${\text{P}}_{k}^{j}$ is *z* ^*i*^′ = *z* ^*i*^(1+*ε* ^*i*^) ($\varepsilon^{i}=\vec{{\text{P}}_{0}^{i}{\text{P}}_{k}^{j}}•k/z^{i}$), the vector $\vec{{\text{P}}_{k}^{i}{\text{Q}}_{k}^{i}}$ is defined as:

$$\vec{{\text{P}}_{k}^{i}{\text{Q}}_{k}^{i}}=(f_{x}^{k}(z^{i}{}^{\prime })-f_{x}^{k}(z^{i}),f_{y}^{k}(z^{i}{}^{\prime })-f_{y}^{k}(z^{i}),0)$$ (8)

The vector $\vec{{\text{Q}}_{k}^{i}{\text{P}}_{k}^{j}}$ is perpendicular to the reference plane Π_*i*_, and it can be defined as:

$$\vec{{\text{Q}}_{k}^{i}{\text{P}}_{k}^{j}}=(0,0,z^{i}\cdot \varepsilon^{i})$$ (9)

The sum of the three vectors can then be expressed as:

$$\vec{{\text{P}}_{0}^{i}{\text{P}}_{k}^{j}}=(f_{x}^{k}(z^{i}{}^{\prime })-f_{x}^{0}(z^{i}),f_{y}^{k}(z^{i}{}^{\prime })-f_{y}^{0}(z^{i}),z^{i}\cdot \varepsilon^{i})$$ (10)

Then take the dot product of Eq (10) with the unit vector **i** and **j**. The dot products $\vec{{\text{P}}_{0}^{i}{\text{P}}_{k}^{j}}•i$ and $\vec{{\text{P}}_{0}^{i}{\text{P}}_{k}^{j}}•j$ are expressed as:

$$\{\begin{array}{c} \vec{{\text{P}}_{0}^{i}{\text{P}}_{k}^{j}}\cdot i=f_{x}^{k}(z^{i}{}^{\prime })-f_{x}^{0}(z^{i})\\ \vec{{\text{P}}_{0}^{i}{\text{P}}_{k}^{j}}\cdot j=f_{y}^{k}(z^{i}{}^{\prime })-f_{y}^{0}(z^{i}) \end{array}$$ (11)

Solving Eq (11) for the unknowns would provide all the information required to define the object pose.

#### Formulations of scaled orthographic projection

The right hand sides of Eq (11), the terms $f_{x}^{k}(z^{i}{}^{\prime })$ and $f_{y}^{k}(z^{i}{}^{\prime })$, are in fact the coordinates of the point ${\text{Q}}_{k}^{i}$, which are the scaled orthographic projections of the feature point ${\text{P}}_{k}^{j}$. Consider the points ${\text{P}}_{0}^{i}$, ${\text{P}}_{k}^{j}$, and the projections ${\text{Q}}_{k}^{i}$ of ${\text{P}}_{k}^{j}$ on the plane Π_*i*_, the vector $\vec{{\text{P}}_{0}^{i}{\text{P}}_{k}^{j}}$ is the sum of two vectors $\vec{{\text{P}}_{0}^{i}{\text{Q}}_{k}^{i}}$ and $\vec{{\text{Q}}_{k}^{i}{\text{P}}_{k}^{j}}$. The vector $\vec{{\text{P}}_{0}^{i}{\text{Q}}_{k}^{i}}$ should be represented as:

$$\vec{{\text{P}}_{0}^{i}{\text{Q}}_{k}^{i}}=(f_{x}^{k}(z^{i}{}^{\prime })-f_{x}^{0}(z^{i}),f_{y}^{k}(z^{i}{}^{\prime })-f_{y}^{0}(z^{i}),0)$$ (12)

Then take the dot product of the vector $\vec{{\text{P}}_{0}^{i}{\text{P}}_{k}^{j}}$ with the unit vector **i**. The dot product $\vec{{\text{Q}}_{k}^{i}{\text{P}}_{k}^{j}}•i$ is zero, and the dot product $\vec{{\text{P}}_{0}^{i}{\text{Q}}_{k}^{i}}•i$ is the *x* coordinate $f_{x}^{k}(z^{i}{}^{\prime })-f_{x}^{0}(z^{i})$. Consequently, the dot products $\vec{{\text{P}}_{0}^{i}{\text{P}}_{k}^{j}}•i$ and $\vec{{\text{P}}_{0}^{i}{\text{P}}_{k}^{j}}•j$ are similar to Eq (11).

### Iteration for scaled orthographic projection

Eq (11) can also be written:

$$\{\begin{array}{c} \vec{{\text{P}}_{0}^{i}{\text{P}}_{k}^{j}}\cdot i=f_{x}^{k}(z^{i}(1+\varepsilon^{i}))-f_{x}^{0}(z^{i})\\ \vec{{\text{P}}_{0}^{i}{\text{P}}_{k}^{j}}\cdot j=f_{y}^{k}(z^{i}(1+\varepsilon^{i}))-f_{y}^{0}(z^{i}) \end{array}$$ (13)

As the points ${\text{P}}_{0}^{i}$ and ${\text{P}}_{0}^{n}$, ${\text{P}}_{k}^{j}$ and ${\text{P}}_{k}^{n}$ respectively locate in the perspective rays l_0_ and l_*k*_, Eq (13) could be approximated as:

$$\{\begin{array}{c} \vec{{\text{P}}_{0}^{i}{\text{P}}_{k}^{j}}\cdot I=f_{x}^{k}(z^{n})-f_{x}^{0}(z^{n})\\ \vec{{\text{P}}_{0}^{i}{\text{P}}_{k}^{j}}\cdot J=f_{y}^{k}(z^{n})-f_{y}^{0}(z^{n}) \end{array}$$ (14)

where **I** = *s*_*i*_·**i**, **j** = *s*_*i*_·**j**. Eq (14) provides a linear system of equations in which the only unknowns are respectively the coordinates of **I** and **J**. The norm of **I** and **J** are respectively the scaling factor *s*_*i*_ and *s*_*j*_ between the vector $\vec{{\text{P}}_{0}^{i}{\text{P}}_{k}^{j}}$ and $\vec{{\text{P}}_{0}^{n}{\text{P}}_{k}^{n}}$. Then the length of the two vectors can be written as:

$$\left|\vec{{\text{P}}_{0}^{n}{\text{P}}_{k}^{n}}\right|=\frac{s_{i}+s_{j}}{2}\left|\vec{{\text{P}}_{0}^{i}{\text{P}}_{k}^{j}}\right|$$ (15)

It can be parameterized as:

$$\left|\vec{{\text{P}}_{0}^{n}{\text{P}}_{k}^{n}}\right|=\frac{s_{i}+s_{j}}{2}\sqrt{{\left(f_{x}^{k}(z^{i}{}^{\prime })-f_{x}^{0}(z^{i})\right)}^{2}+{\left(f_{y}^{k}(z^{i}{}^{\prime })-f_{y}^{0}(z^{i})\right)}^{2}+{\left(z^{i}\cdot \varepsilon^{i}\right)}^{2}}$$ (16)

If values are given to the term *ε* ^*i*^, *z* ^*i*^ is obtained from Eq (16).

The proposed algorithm, used to determine the pose by solving the linear system, is called perspective-ray-based scaled orthographic projection (PRSO). The solution of the PRSO algorithm is only an approximation if the values given to the term *ε* ^*i*^ are not exact. But once the unknowns **i** and **j** have been computed, more exact values can be computed for the term *ε* ^*i*^, and the equations can be solved again with these better values. The iteration algorithm is named PRSOI (PRSO with Iterations). It generally makes the values of **i**, **j** and *z* ^*i*^ converge towards values which correspond to a correct pose through iterations.

Initially, the term *ε* ^*i*^ is equal to zero. In fact, it can be assumed that ${\text{P}}_{k}^{j}$ and ${\text{Q}}_{k}^{i}$ coincide. When tracking an object, the initial value for the term *ε* ^*i*^ is preferably chosen equal to the value obtained at the last iteration of the pose estimation for the previous image. The computed error of coordinates, which is between the projection point ${\text{Q}}_{k}^{n}$ of ${\text{P}}_{k}^{j}$ in the prior iteration and the one in the current iteration, reaches the minimum at the end of iterations.

### Solving the system of PRSO algorithm

Within the preceding iterative algorithm, the solution of Eq (14) is still a problem. This equation could be rewritten in a more compact form:

$$\{\begin{array}{c} \vec{{\text{P}}_{0}^{i}{\text{P}}_{k}^{j}}\cdot I=\xi^{i}\\ \vec{{\text{P}}_{0}^{i}{\text{P}}_{k}^{j}}\cdot J=\eta^{i} \end{array}$$ (17)

where $\xi^{i}=f_{x}^{k}(z^{n})-f_{x}^{0}(z^{n})$,$\eta^{i}=f_{y}^{k}(z^{n})-f_{y}^{0}(z^{n})$. The dot products of this equation are expressed in terms of vector coordinates in the object coordinate frame:

$$\{\begin{array}{l} {}[\begin{array}{ccc} u_{i} & v_{i} & w_{i} \end{array}]{\left[{\begin{array}{ccc} i_{u} & i_{v} & i \end{array}}_{w}\right]}^{T}=\xi^{i}\\ {}[\begin{array}{ccc} u_{i} & v_{i} & w_{i} \end{array}]{\left[{\begin{array}{ccc} j_{u} & j_{v} & j \end{array}}_{w}\right]}^{T}=\eta^{i} \end{array}$$ (18)

These are linear equations where the unknowns are the coordinates of **I** and **J**. The other parameters are known: $f_{x}^{0}$,$f_{y}^{0}$,$f_{x}^{k}$,$f_{y}^{k}$ are the known functions of l_*0*_ and l_*k*_, and *u*_*i*_, *v*_*i*_, *w*_*i*_ are the known coordinates of ${\text{P}}_{k}^{j}$ in the object coordinate frame. Substitute the *n* feature points for Eq (18), a linear system is generated for the coordinates of the unknown vectors **I** and **J**:

$$\{\begin{array}{l} A\cdot I=x^{\prime }\\ A\cdot J=y^{\prime } \end{array}$$ (19)

where **A** is the matrix of the coordinates of the object points in the object coordinate frame, $x^{\prime }={\left[\begin{array}{lllll} \xi_{0}^{i} & \cdots & \xi_{j}^{i} & \cdots & \xi_{n}^{i} \end{array}\right]}^{T}$, $y^{\prime }={\left[\begin{array}{lllll} \eta_{0}^{i} & \cdots & \eta_{j}^{i} & \cdots & \eta_{n}^{i} \end{array}\right]}^{T}$. In general, if there are at least four non-coplanar points, the least square solution of the linear system is given by:

$$\{\begin{array}{l} Ι=B\cdot x^{\prime }\\ J=B\cdot y^{\prime } \end{array}$$ (20)

where the object matrix **B** is the pseudo inverse of the matrix **A**. Once the least square solutions to **I** and **J** are obtained, the unit vectors **i** and **j** are simply obtained by normalizing **I** and **J**.

Now the translation vector **T** of the object can be obtained. It is vector $\vec{{\text{OP}}_{0}^{i}}$, and *z* ^*i*^ is computed by Eq (16). Then the vector **T** is computed by Eq (5).

## Experiment Results

### Camera calibration results

In the experiment, a domestically developed CCD camera with image resolution 768×576 pixels, pixel size 0.0083mm×0.0086mm, and field angle 60°, is used. It is fixed on a linear stage via a bracket. The type of the linear stage is Zolix KSA300-11-X, with repeatability of 3μm, straightness of 10μm, and travel of 300mm. The calibration target is a solid circular array pattern with 7×9 circular points evenly distributed. The size of the target is 500×600mm^2^, and the distance between the adjacent points is 60mm in the horizontal and vertical directions.

Fix the target on the optical platform, and then move the camera to make the calibration target cover most of the field of view. The captured images are taken at six different positions, and the distance between the adjacent positions is 30mm. Two specific images, such as the images captured at 0mm and 150mm, are regarded as the calibration data. As the camera parameters are obtained, the captured images, including the two specific ones, are introduced into the IRT to compute the space error of the calibration points. The captured images are shown in Fig 6.

Table 1 lists the camera parameters. The reference planes Π_*m*_ and Π_*n*_ are described as the fifth order polynomials.

**Table 1:** Camera parameters.

Parameters	Π_*m*_(0mm)		Π_*n*_(150mm)	
	*$g_{x}^{m}\left(u,v\right)$*	*$g_{y}^{m}\left(u,v\right)$*	*$g_{x}^{n}\left(u,v\right)$*	*$g_{y}^{n}\left(u,v\right)$*
**(*C***_**00**_ **, *D***_**00**_ **)**	-2.654E+02	-1.865E+02	-3.226E+02	-2.208E+02
**(*C***_**10**_ **, *D***_**10**_ **)**	6.726E-01	-4.893E-04	8.756E-01	-8.562E-03
**(*C***_**01**_ **, *D***_**01**_ **)**	2.479E-02	7.093E-01	2.791E-03	8.171E-01
**(*C***_**20**_ **, *D***_**20**_ **)**	9.571E-05	-1.246E-05	-2.688E-04	2.292E-05
**(*C***_**11**_ **, *D***_**11**_ **)**	-1.179E-04	-1.156E-04	1.512E-04	8.542E-05
**(*C***_**02**_ **, *D***_**02**_ **)**	1.432E-06	-1.468E-05	-6.406E-05	8.410E-05
**(*C***_**30**_ **, *D***_**30**_ **)**	-3.328E-07	2.558E-08	5.994E-07	-9.413E-08
**(*C***_**21**_ **, *D***_**21**_ **)**	4.067E-07	1.902E-07	-6.830E-07	-3.960E-07
**(*C***_**12**_ **, *D***_**12**_ **)**	-2.716E-08	5.363E-08	-8.002E-08	-2.445E-07
**(*C***_**03**_ **, *D***_**03**_ **)**	-6.565E-08	-8.220E-09	2.443E-07	-1.878E-07
**(*C***_**40**_ **, *D***_**40**_ **)**	5.180E-10	-6.346E-11	-5.699E-10	1.449E-10
**(*C***_**31**_ **, *D***_**31**_ **)**	-7.111E-10	-8.815E-12	1.226E-09	8.289E-10
**(*C***_**22**_ **, *D***_**22**_ **)**	-2.782E-12	1.190E-10	-4.663E-10	1.900E-10
**(*C***_**13**_ **, *D***_**13**_ **)**	-3.097E-11	-1.939E-10	3.740E-10	8.478E-11
**(*C***_**04**_ **, *D***_**04**_ **)**	2.582E-10	3.799E-11	-1.669E-11	5.031E-10
**(*C***_**50**_ **, *D***_**50**_ **)**	-2.645E-13	4.620E-14	2.081E-13	-9.186E-14
**(*C***_**41**_ **, *D***_**41**_ **)**	4.766E-13	-1.756E-13	-7.830E-13	-6.358E-13
**(*C***_**32**_ **, *D***_**32**_ **)**	4.283E-14	-1.214E-13	3.333E-13	-5.590E-14
**(*C***_**23**_ **, *D***_**23**_ **)**	-1.650E-13	1.326E-13	-1.334E-13	-1.493E-13
**(*C***_**14**_ **, *D***_**14**_ **)**	-5.200E-14	-2.771E-13	2.536E-13	-3.852E-13
**(*C***_**05**_ **, *D***_**05**_ **)**	1.835E-14	3.582E-13	-5.181E-13	2.097E-13

**Fig 7:**
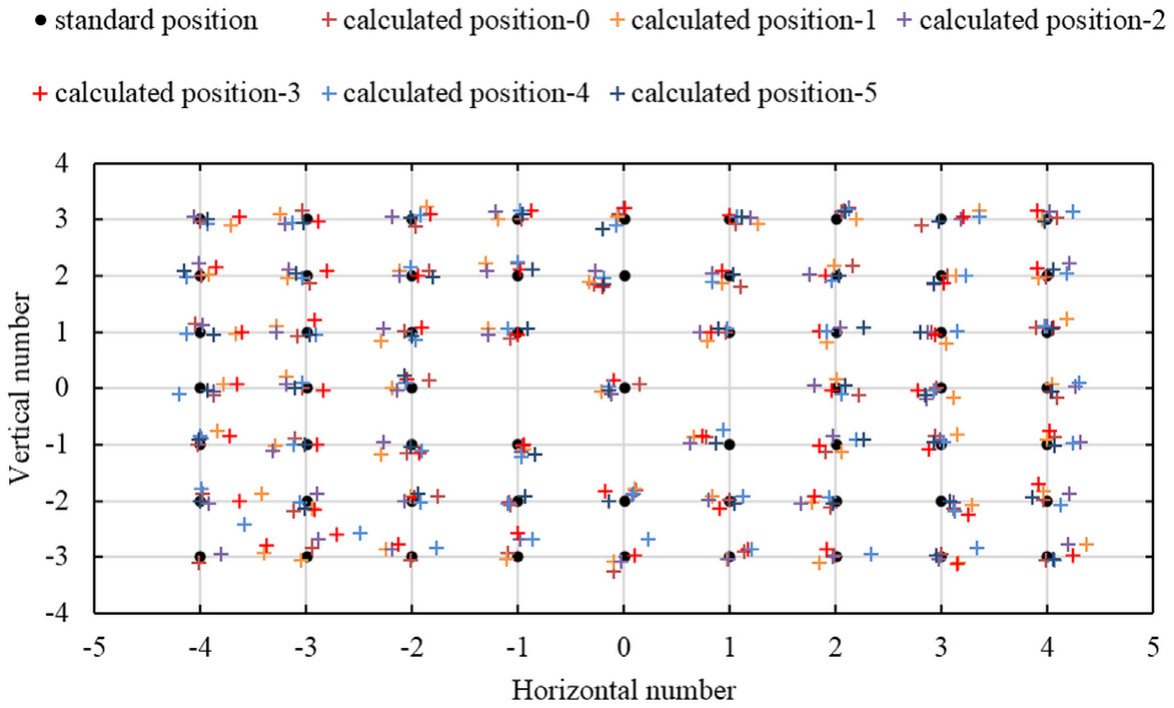
Position distribution of the calibration points. The “●” represents the standard two-dimensional coordinate of the calibration points while the “+” represents the calculated two-dimensional coordinate of them.

Fig 7 shows the position distribution of the calibration points. The standard position is the standard coordinates of the calibration points, and the calculated position is the calculated coordinates of the calibration points obtained by the IRT and the image coordinates from the captured images.

The root mean square error (RMSE) of the calculated calibration points is 0.17mm in horizontal direction, and 0.12mm in vertical direction. According to the error statistics of the calibration points, it is obvious that the camera can be described by the IRT completely.

### Pose estimation results

**Fig 8:**
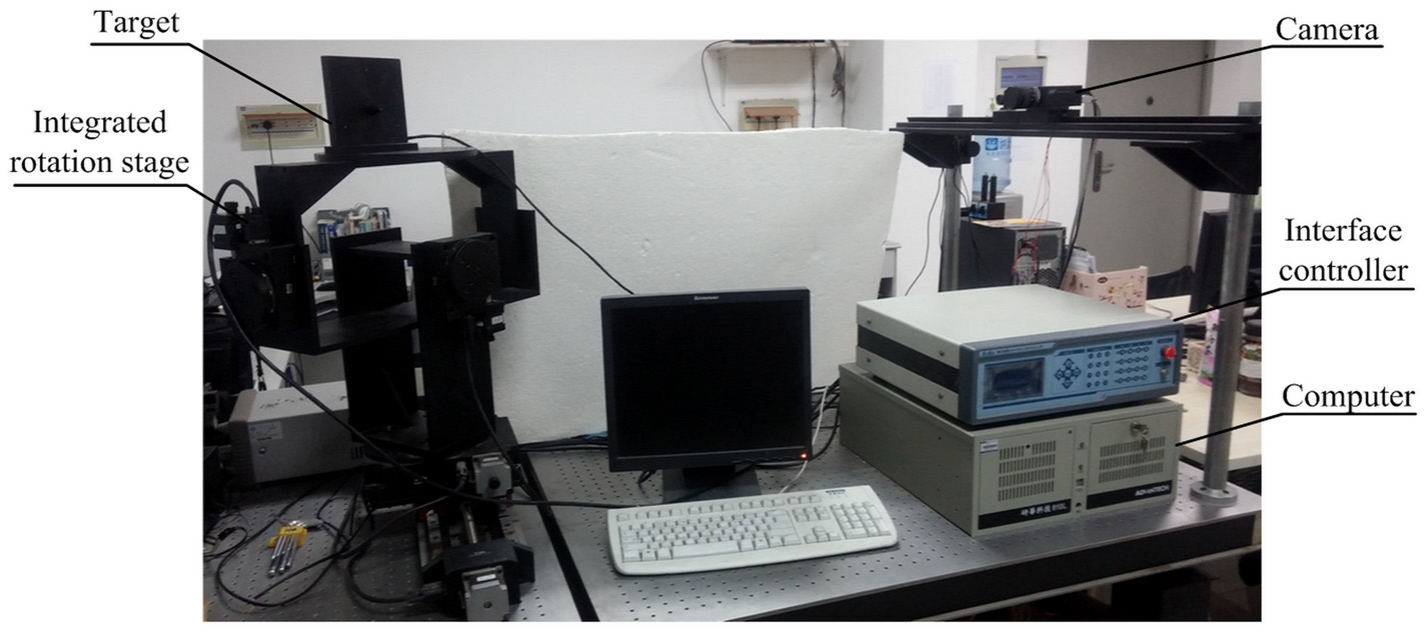
Experiment devices.

The experiment devices for pose estimation are shown in Fig 8. The integrated rotation stage is composed of three rotation stages: Zolix RAK-200 in the yaw direction, Zolix RAK-100 in the pitch and roll directions. The repeatability of the RAK-200 is 0.005°, load 50kg. The repeatability of the RAk-100 is 0.005°, load 30kg. The type of interface controller is Zolix MC600-4B, two-phase stepping motor, closed-loop control. The codes of the P4P solutions are run in Microsoft Visual Studio 2010 environment on a computer with 3.40 GHz CPU.

**Fig 9:**
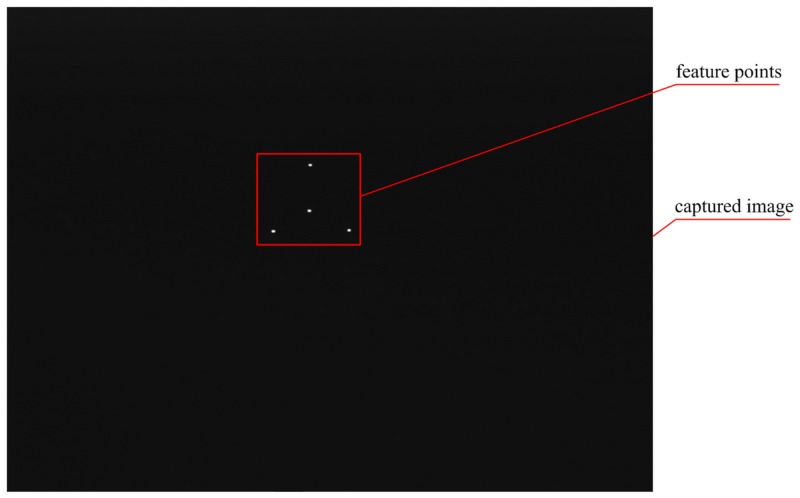
A sample image used for pose estimation.

During the experimental process, the target is fixed on the rotating platform, and the image is captured at every 1°. The rotating angle of the target between the initial position and the current position is measured by the two captured images. The three directions of rotational motion are tested. Then fix the target on the linear stage, and capture the image of it at every 2mm. The moving distance of the target between the initial position and the current position is also measured by the two captured images. The three directions of translational motion are tested. Fig 9 shows a real image of four non-coplanar feature points captured by the calibrated camera.

**Fig 10:**
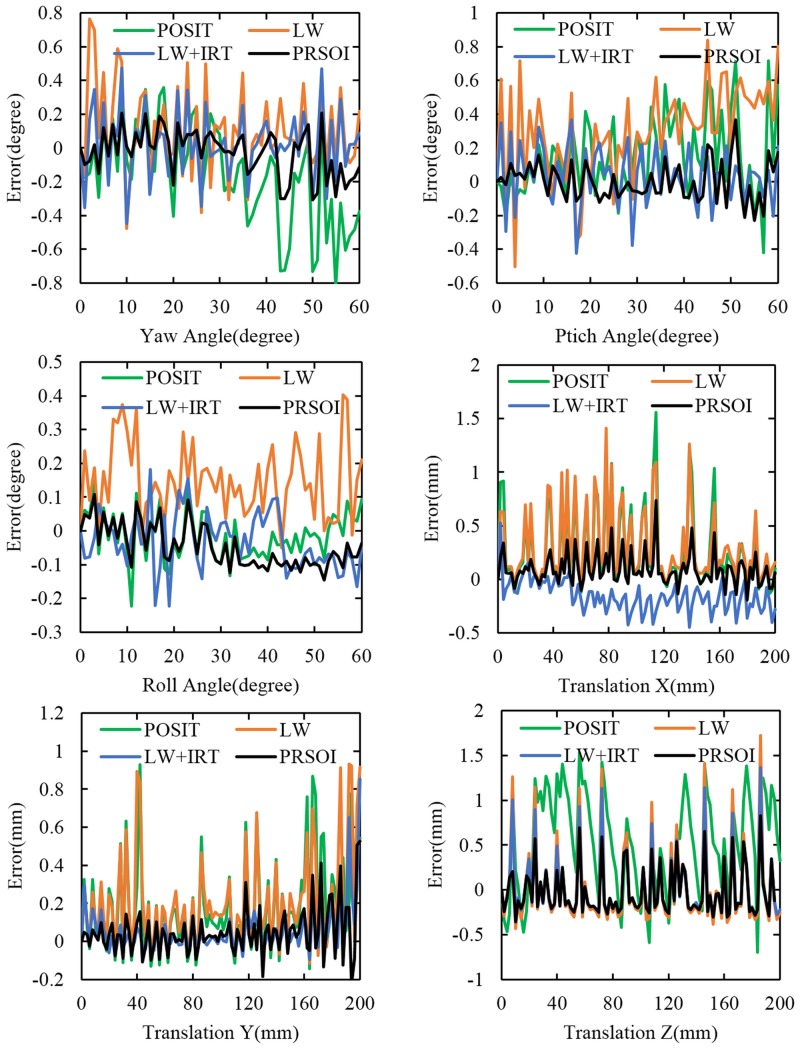
Pose estimation error distribution.

Notice the central part in the Fig 9: the effective coverage of the four feature points in the captured image is just about 1.49%. This is very different from the captured image of the popular P*n*P solutions. The PRSOI is tested by the captured data, and compared with the state-of-the-art P4P solutions. For the pinhole camera, the geometric configuration solution by Liu ML and Wong KH [20], denoted by LW in short, as well as the popular iterative solution POSIT [13], are considered. For the IRT camera, the LW+IRT solution is considered, since the LW incorporates the IRT. The results of the P4P solutions are shown in Fig 10. The calculated pose of the target are checked by comparison with the standard positions which are obtained from the interface controller.

Statistics are used in estimation error analysis, and the RMSE of the P4P solutions are summarized in Table 2.

**Table 2:** The RMSE of the PnP algorithms.

method	*e*_*ry*_ ^a^(*deg*.)	*e*_*rp*_ ^b^(*deg*.)	*e*_*rr*_ ^c^(*deg*.)	*e*_*tx*_ ^d^(*mm*)	*e*_*ty*_ ^e^(*mm*)	*e*_*tz*_ ^f^(*mm*)
**POSIT**	0.290	0.243	0.071	0.369	0.241	0.552
**LW**	0.258	0.277	0.110	0.340	0.248	0.448
**LW+IRT**	0.201	0.176	0.083	0.146	0.130	0.362
**PRSOI**	0.136	0.115	0.062	0.152	0.128	0.272

^a^ry is rotation in yaw direction,

^b^rp is rotation in pitch direction,

^c^rr is rotation in roll direction,

^d^tx is translation in x direction,

^e^ty is translation in y direction, and

^f^tz is translation in z direction.

Through the comparison between the LW and the LW+IRT, it is obvious that the accuracy of the LW+IRT is higher than that of the LW. The result suggested that the IRT is effective in the P4P solutions. As the accuracy of the PRSOI is higher than that of the POSIT, it demonstrates that the perspective-ray-based scaled orthographic projection is superior to the scaled orthographic projection in a pinhole camera. Considering the accuracy of the four P4P solutions, it can be proved that the accuracy of the PRSOI outperforms the other three state-of-the-art P4P solutions.

The PRSOI is an iterative solution, though powerful, does have a shortfall: planning the correct pose for each position is slow. In this paper, accuracy is the major concern while computational cost is ignored.

## Conclusion

This paper puts forward and deeply analyzes the IRT and the PRSOI. The IRT, which with definite geometric meaning, consists of two reference planes Π_*m*_ and Π_*n*_. The PRSOI introduces the IRT into a scaled orthographic projection, then adopts an iteration to make the perspective-ray-based scaled orthographic projection more accurate. Four non-coplanar points are used as feature points in the real image experiment. And three other P4P solutions are introduced to be compared with the PRSOI. Experiment results demonstrated that the PRSOI is of high accuracy in the six-DOF motion. The P4P solution proposed in this paper is of significance in the P4P applications such as the positioning of mechanical arm, the four-wheel aligners, the installation of super-huge workpiece, etc..

To the best of our knowledge, it is the first study to incorporate the perspective ray with the scaled orthographic projection, and the incorporation works effectively in the P4P situation.

## Supporting Information

S1 Dataset Camera captured dataset. This archive contains the captured data files used as the basis for the P4P solutions described in the manuscript. The data are provided in a directory hierarchy where each degree of freedom has a separate directory. And the calibration data is the captured data used in the camera calibration.
(ZIP)
