# Supplementary figures and images for: A New Pose Estimation Algorithm Using a Perspective-Ray-Based Scaled Orthographic Projection with Iteration (part 2 of 2)
Source: PLoS One. 2015 Jul 21;10(7):e0134029. doi: 10.1371/journal.pone.0134029 (PMC4509906; doi:10.1371/journal.pone.0134029)

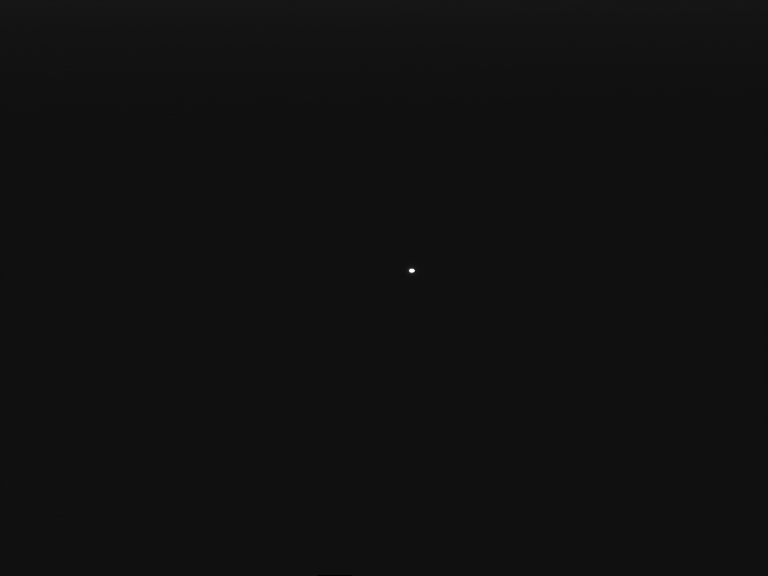

Supplement: S1 Dataset — This archive contains the captured data files used as the basis for the P4P solutions described in the manuscript. The data are provided in a directory hierarchy where each degree of freedom has a separate directory. And the calibration data is the captured data used in the camera calibration. (ZIP) [file pone.0134029.s001.zip › S1_Dataset/Pitch Angle/(3,0,0,2).tif]

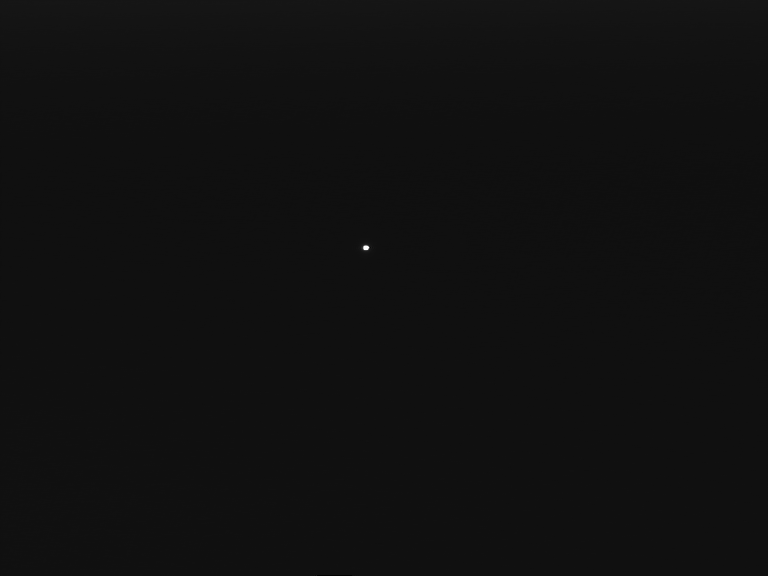

Supplement: S1 Dataset — This archive contains the captured data files used as the basis for the P4P solutions described in the manuscript. The data are provided in a directory hierarchy where each degree of freedom has a separate directory. And the calibration data is the captured data used in the camera calibration. (ZIP) [file pone.0134029.s001.zip › S1_Dataset/Pitch Angle/(3,0,0,3).tif]

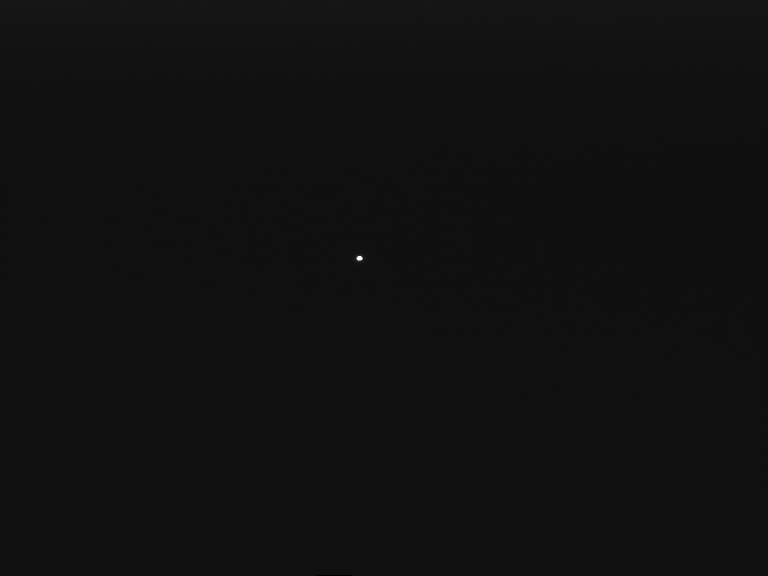

Supplement: S1 Dataset — This archive contains the captured data files used as the basis for the P4P solutions described in the manuscript. The data are provided in a directory hierarchy where each degree of freedom has a separate directory. And the calibration data is the captured data used in the camera calibration. (ZIP) [file pone.0134029.s001.zip › S1_Dataset/Pitch Angle/(30,0,0,0).tif]

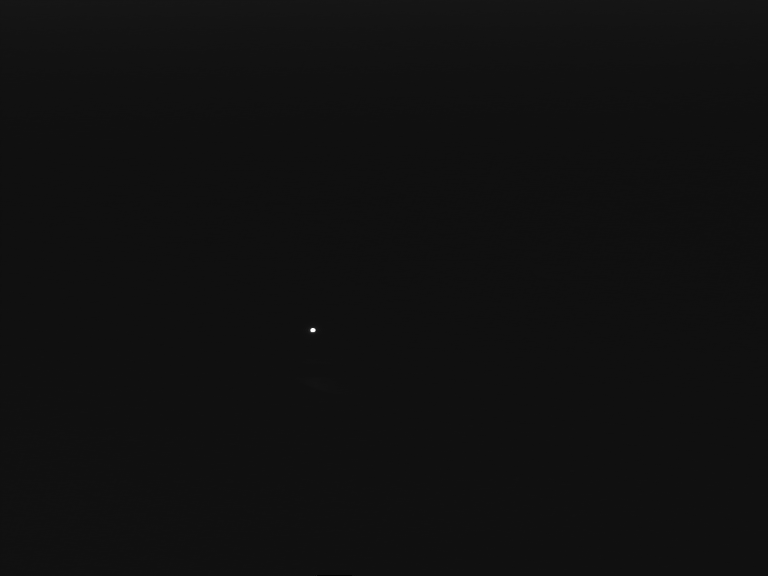

Supplement: S1 Dataset — This archive contains the captured data files used as the basis for the P4P solutions described in the manuscript. The data are provided in a directory hierarchy where each degree of freedom has a separate directory. And the calibration data is the captured data used in the camera calibration. (ZIP) [file pone.0134029.s001.zip › S1_Dataset/Pitch Angle/(30,0,0,1).tif]

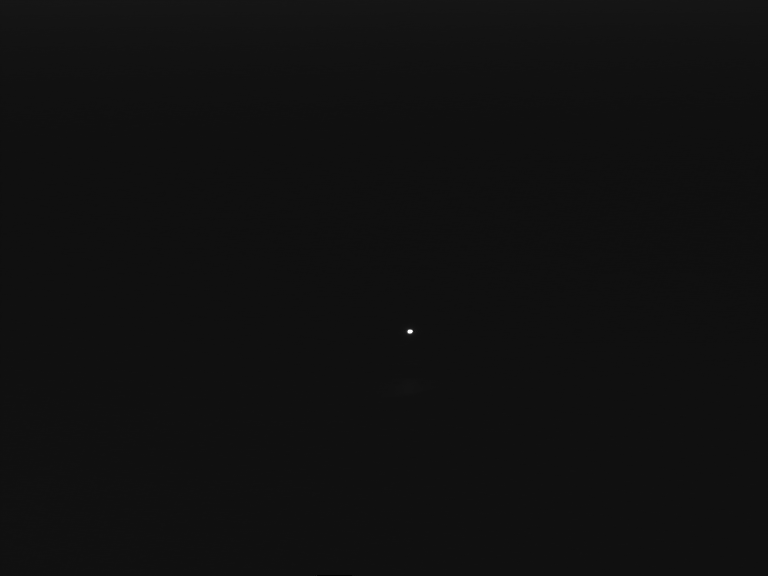

Supplement: S1 Dataset — This archive contains the captured data files used as the basis for the P4P solutions described in the manuscript. The data are provided in a directory hierarchy where each degree of freedom has a separate directory. And the calibration data is the captured data used in the camera calibration. (ZIP) [file pone.0134029.s001.zip › S1_Dataset/Pitch Angle/(30,0,0,2).tif]

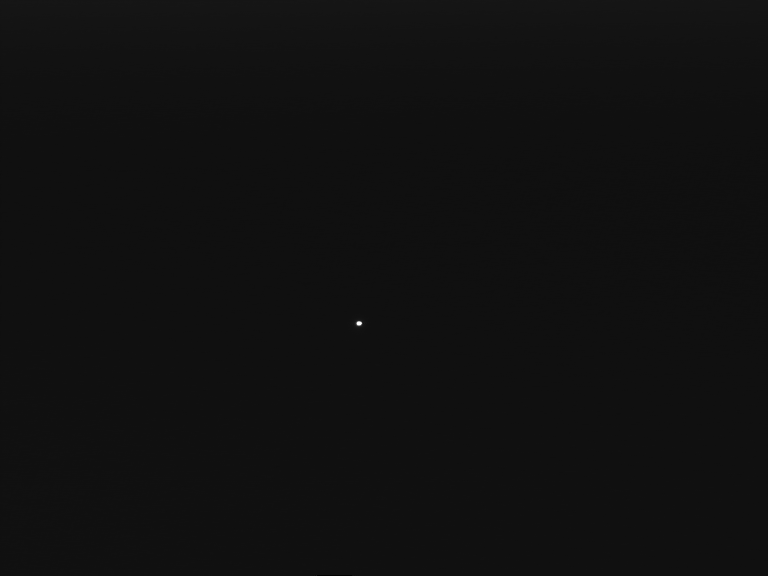

Supplement: S1 Dataset — This archive contains the captured data files used as the basis for the P4P solutions described in the manuscript. The data are provided in a directory hierarchy where each degree of freedom has a separate directory. And the calibration data is the captured data used in the camera calibration. (ZIP) [file pone.0134029.s001.zip › S1_Dataset/Pitch Angle/(30,0,0,3).tif]

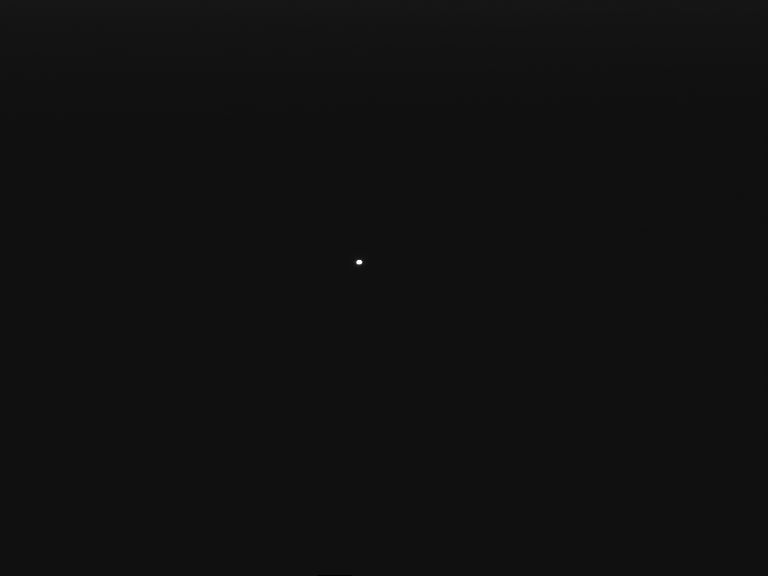

Supplement: S1 Dataset — This archive contains the captured data files used as the basis for the P4P solutions described in the manuscript. The data are provided in a directory hierarchy where each degree of freedom has a separate directory. And the calibration data is the captured data used in the camera calibration. (ZIP) [file pone.0134029.s001.zip › S1_Dataset/Pitch Angle/(31,0,0,0).tif]

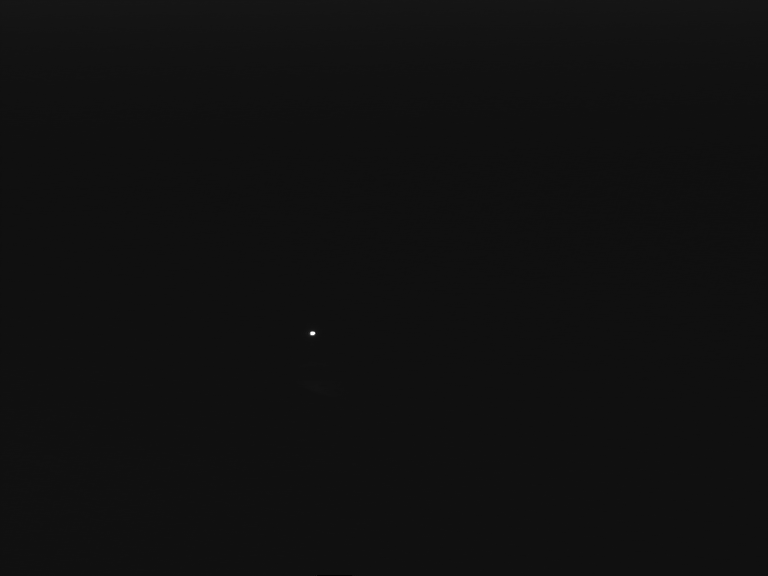

Supplement: S1 Dataset — This archive contains the captured data files used as the basis for the P4P solutions described in the manuscript. The data are provided in a directory hierarchy where each degree of freedom has a separate directory. And the calibration data is the captured data used in the camera calibration. (ZIP) [file pone.0134029.s001.zip › S1_Dataset/Pitch Angle/(31,0,0,1).tif]

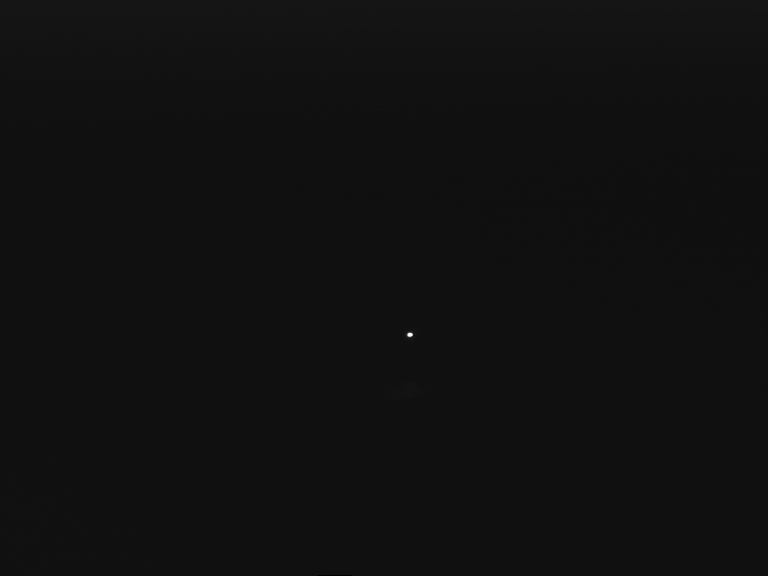

Supplement: S1 Dataset — This archive contains the captured data files used as the basis for the P4P solutions described in the manuscript. The data are provided in a directory hierarchy where each degree of freedom has a separate directory. And the calibration data is the captured data used in the camera calibration. (ZIP) [file pone.0134029.s001.zip › S1_Dataset/Pitch Angle/(31,0,0,2).tif]

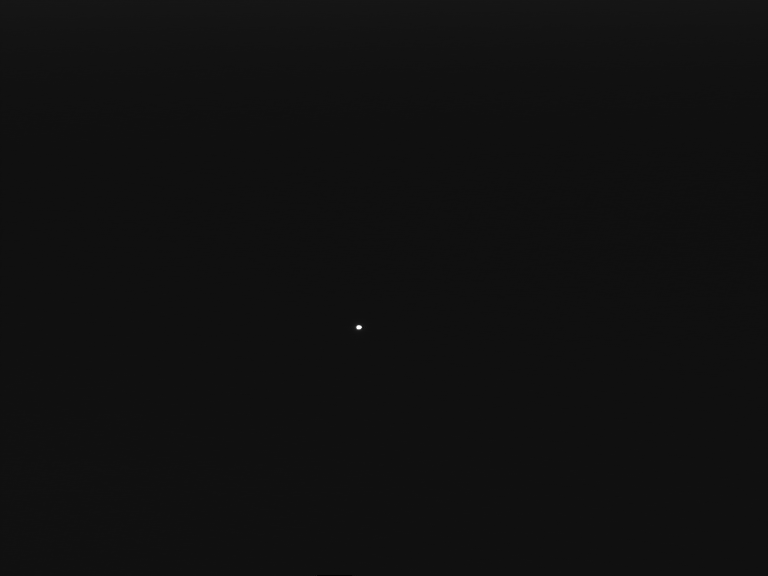

Supplement: S1 Dataset — This archive contains the captured data files used as the basis for the P4P solutions described in the manuscript. The data are provided in a directory hierarchy where each degree of freedom has a separate directory. And the calibration data is the captured data used in the camera calibration. (ZIP) [file pone.0134029.s001.zip › S1_Dataset/Pitch Angle/(31,0,0,3).tif]

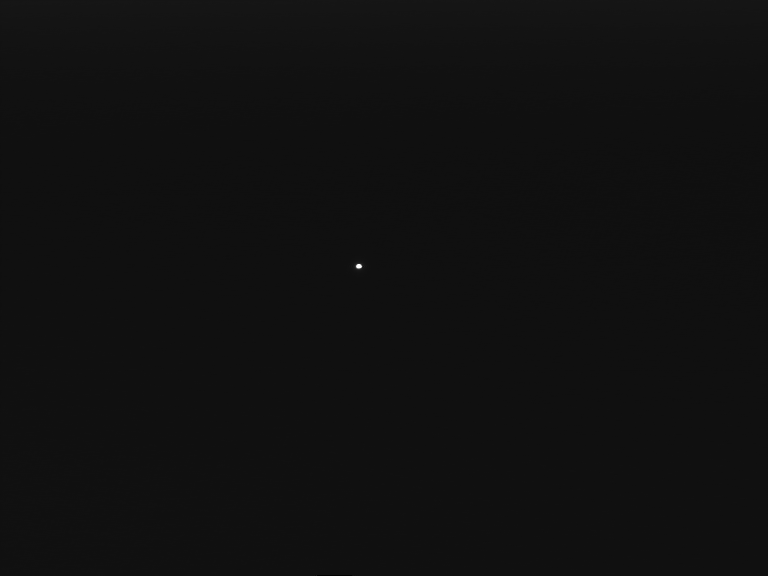

Supplement: S1 Dataset — This archive contains the captured data files used as the basis for the P4P solutions described in the manuscript. The data are provided in a directory hierarchy where each degree of freedom has a separate directory. And the calibration data is the captured data used in the camera calibration. (ZIP) [file pone.0134029.s001.zip › S1_Dataset/Pitch Angle/(32,0,0,0).tif]

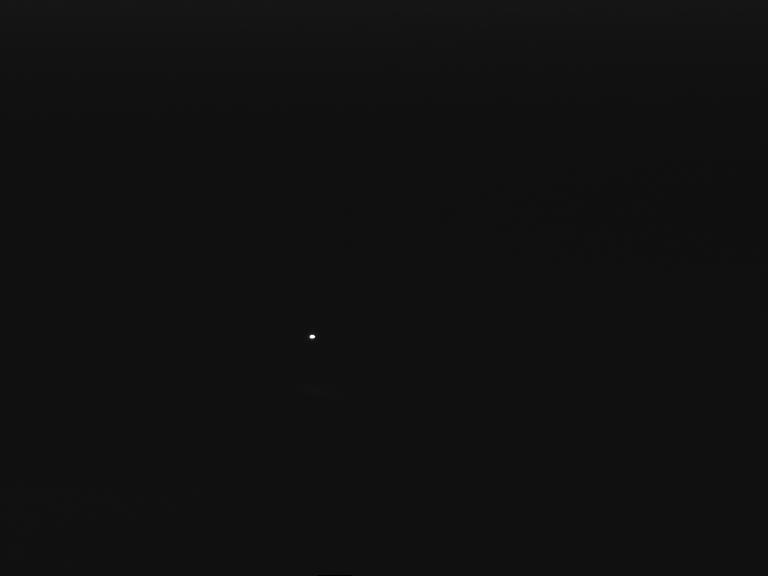

Supplement: S1 Dataset — This archive contains the captured data files used as the basis for the P4P solutions described in the manuscript. The data are provided in a directory hierarchy where each degree of freedom has a separate directory. And the calibration data is the captured data used in the camera calibration. (ZIP) [file pone.0134029.s001.zip › S1_Dataset/Pitch Angle/(32,0,0,1).tif]

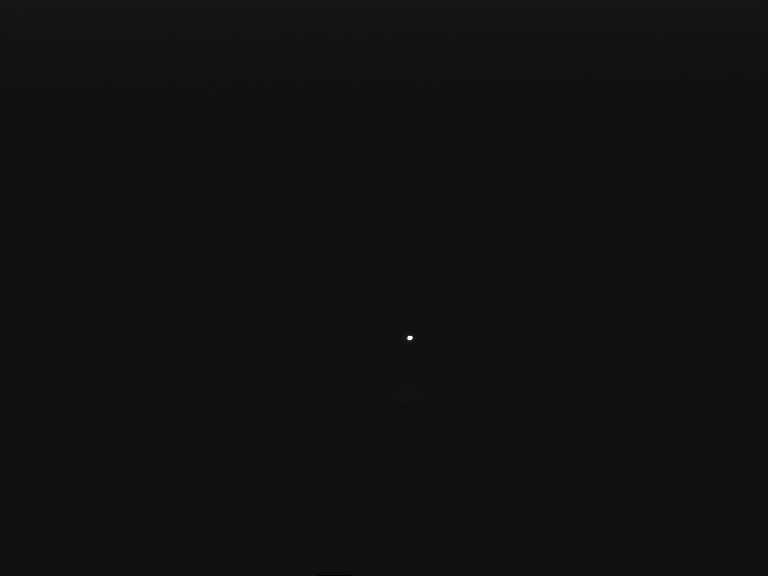

Supplement: S1 Dataset — This archive contains the captured data files used as the basis for the P4P solutions described in the manuscript. The data are provided in a directory hierarchy where each degree of freedom has a separate directory. And the calibration data is the captured data used in the camera calibration. (ZIP) [file pone.0134029.s001.zip › S1_Dataset/Pitch Angle/(32,0,0,2).tif]

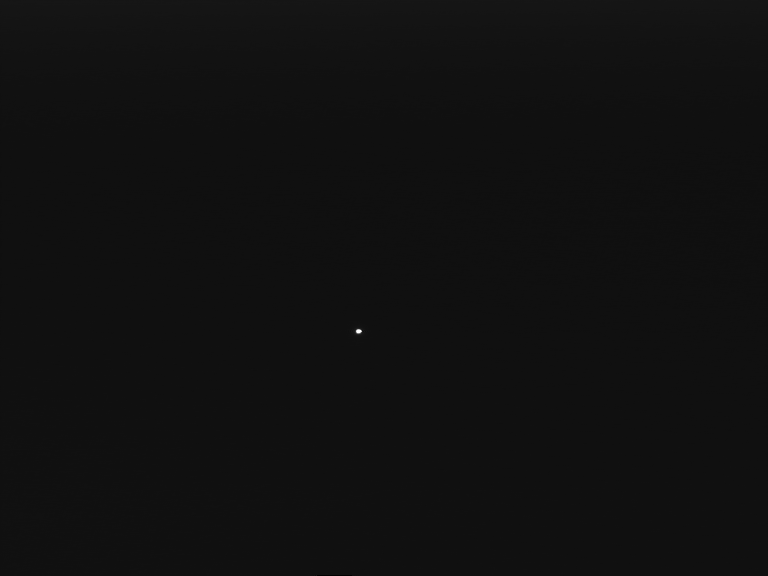

Supplement: S1 Dataset — This archive contains the captured data files used as the basis for the P4P solutions described in the manuscript. The data are provided in a directory hierarchy where each degree of freedom has a separate directory. And the calibration data is the captured data used in the camera calibration. (ZIP) [file pone.0134029.s001.zip › S1_Dataset/Pitch Angle/(32,0,0,3).tif]

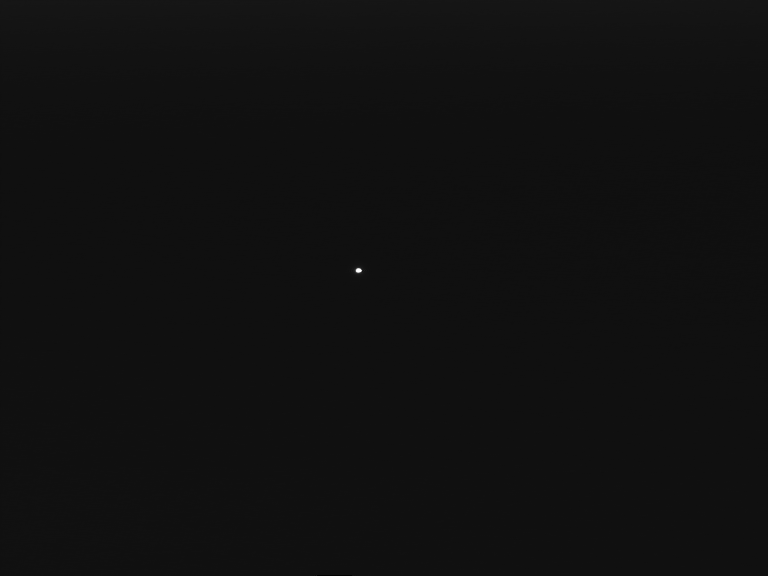

Supplement: S1 Dataset — This archive contains the captured data files used as the basis for the P4P solutions described in the manuscript. The data are provided in a directory hierarchy where each degree of freedom has a separate directory. And the calibration data is the captured data used in the camera calibration. (ZIP) [file pone.0134029.s001.zip › S1_Dataset/Pitch Angle/(33,0,0,0).tif]

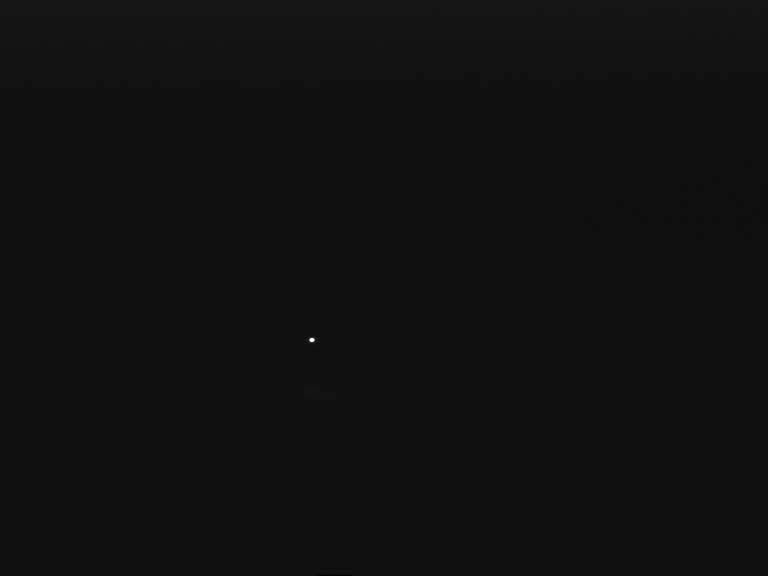

Supplement: S1 Dataset — This archive contains the captured data files used as the basis for the P4P solutions described in the manuscript. The data are provided in a directory hierarchy where each degree of freedom has a separate directory. And the calibration data is the captured data used in the camera calibration. (ZIP) [file pone.0134029.s001.zip › S1_Dataset/Pitch Angle/(33,0,0,1).tif]

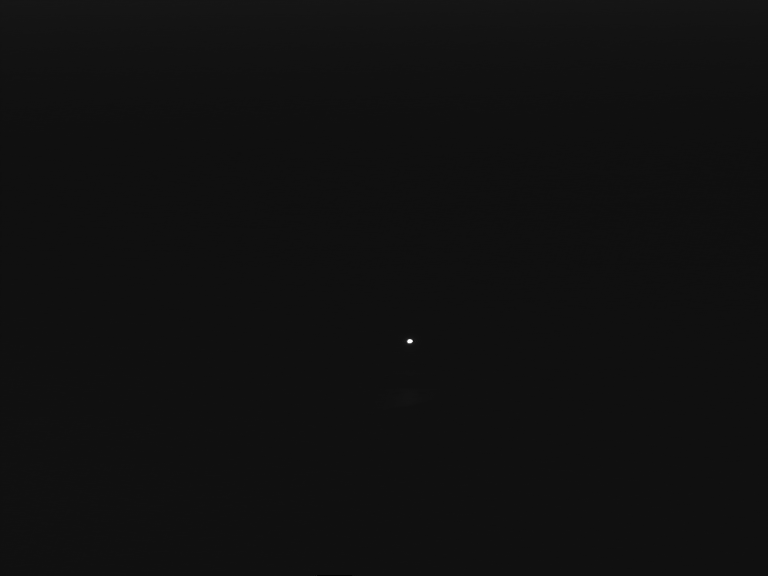

Supplement: S1 Dataset — This archive contains the captured data files used as the basis for the P4P solutions described in the manuscript. The data are provided in a directory hierarchy where each degree of freedom has a separate directory. And the calibration data is the captured data used in the camera calibration. (ZIP) [file pone.0134029.s001.zip › S1_Dataset/Pitch Angle/(33,0,0,2).tif]

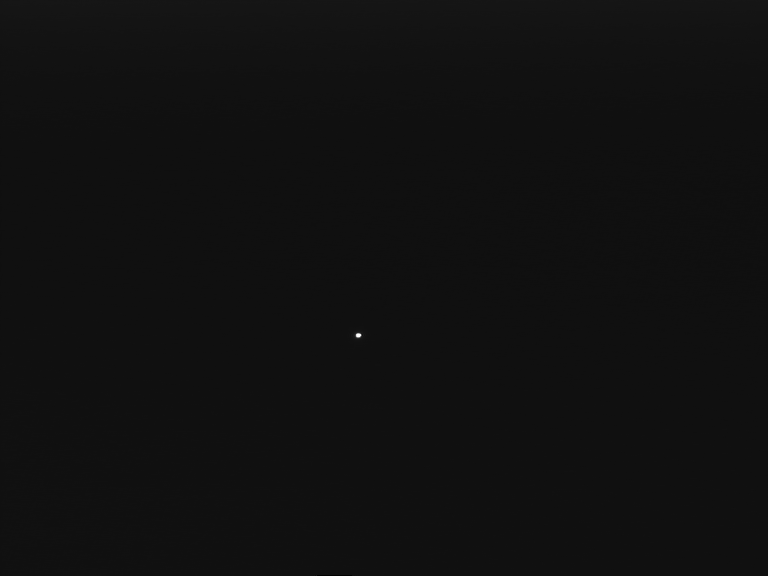

Supplement: S1 Dataset — This archive contains the captured data files used as the basis for the P4P solutions described in the manuscript. The data are provided in a directory hierarchy where each degree of freedom has a separate directory. And the calibration data is the captured data used in the camera calibration. (ZIP) [file pone.0134029.s001.zip › S1_Dataset/Pitch Angle/(33,0,0,3).tif]

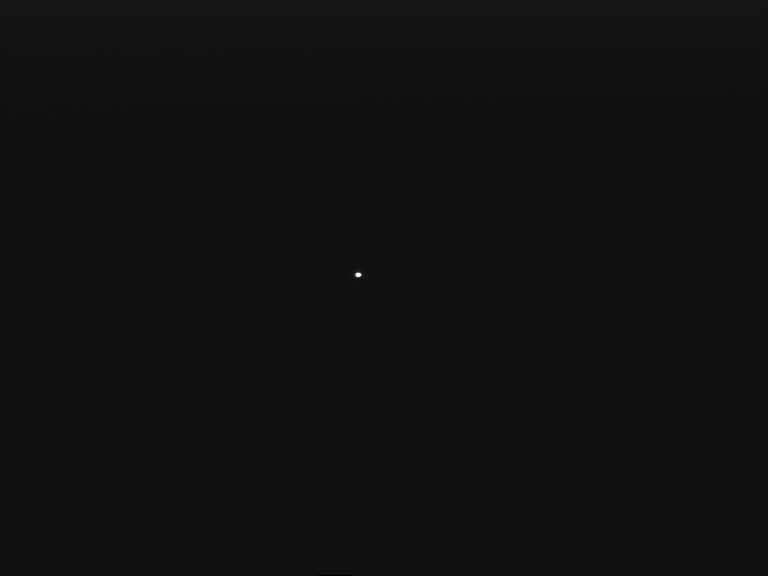

Supplement: S1 Dataset — This archive contains the captured data files used as the basis for the P4P solutions described in the manuscript. The data are provided in a directory hierarchy where each degree of freedom has a separate directory. And the calibration data is the captured data used in the camera calibration. (ZIP) [file pone.0134029.s001.zip › S1_Dataset/Pitch Angle/(34,0,0,0).tif]

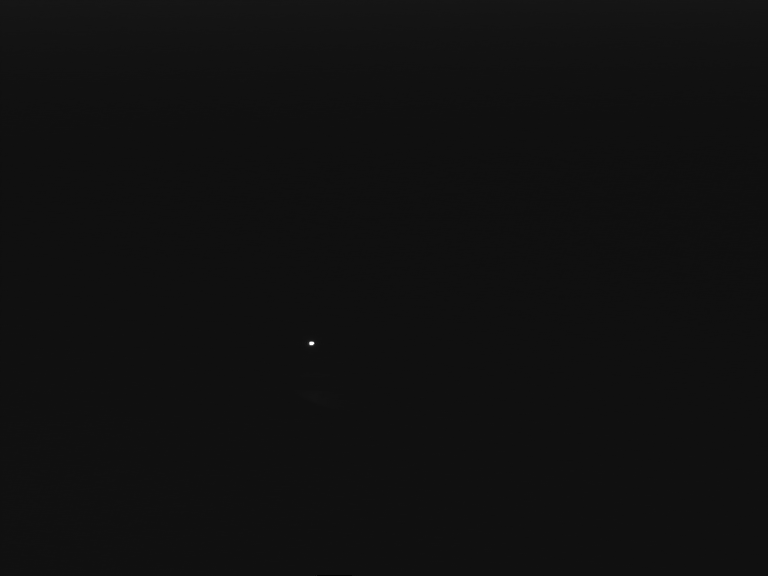

Supplement: S1 Dataset — This archive contains the captured data files used as the basis for the P4P solutions described in the manuscript. The data are provided in a directory hierarchy where each degree of freedom has a separate directory. And the calibration data is the captured data used in the camera calibration. (ZIP) [file pone.0134029.s001.zip › S1_Dataset/Pitch Angle/(34,0,0,1).tif]

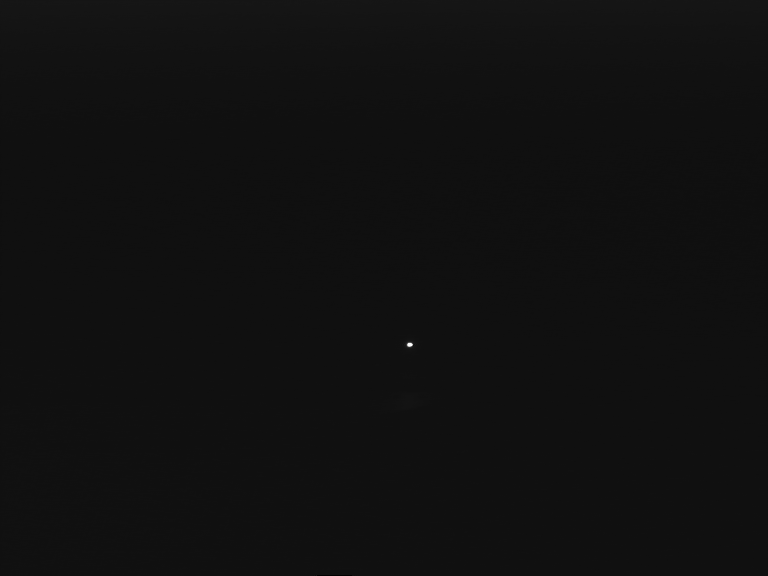

Supplement: S1 Dataset — This archive contains the captured data files used as the basis for the P4P solutions described in the manuscript. The data are provided in a directory hierarchy where each degree of freedom has a separate directory. And the calibration data is the captured data used in the camera calibration. (ZIP) [file pone.0134029.s001.zip › S1_Dataset/Pitch Angle/(34,0,0,2).tif]

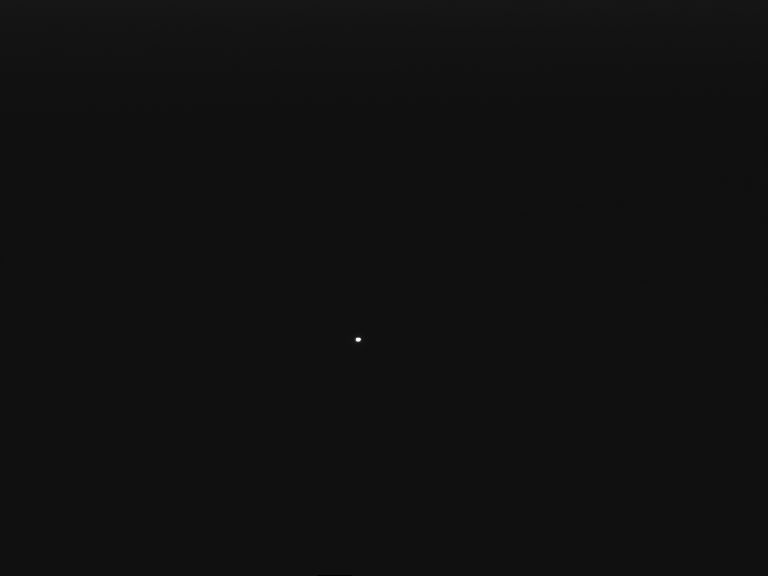

Supplement: S1 Dataset — This archive contains the captured data files used as the basis for the P4P solutions described in the manuscript. The data are provided in a directory hierarchy where each degree of freedom has a separate directory. And the calibration data is the captured data used in the camera calibration. (ZIP) [file pone.0134029.s001.zip › S1_Dataset/Pitch Angle/(34,0,0,3).tif]

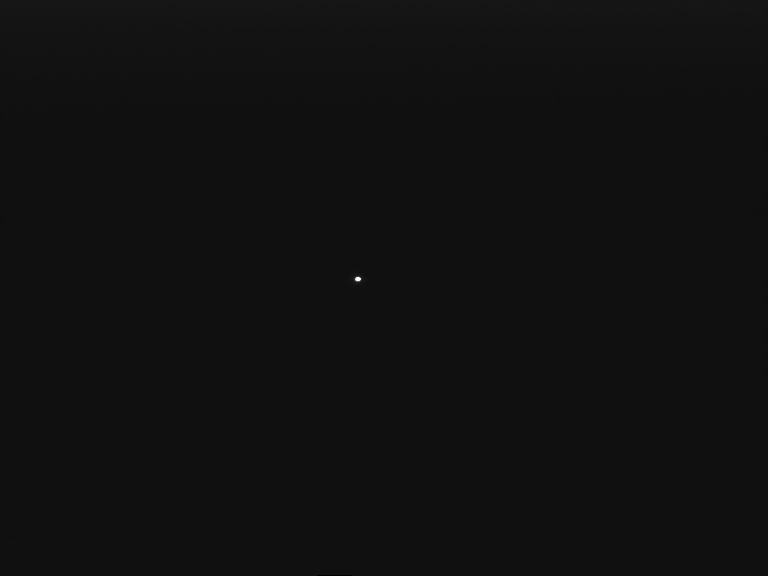

Supplement: S1 Dataset — This archive contains the captured data files used as the basis for the P4P solutions described in the manuscript. The data are provided in a directory hierarchy where each degree of freedom has a separate directory. And the calibration data is the captured data used in the camera calibration. (ZIP) [file pone.0134029.s001.zip › S1_Dataset/Pitch Angle/(35,0,0,0).tif]

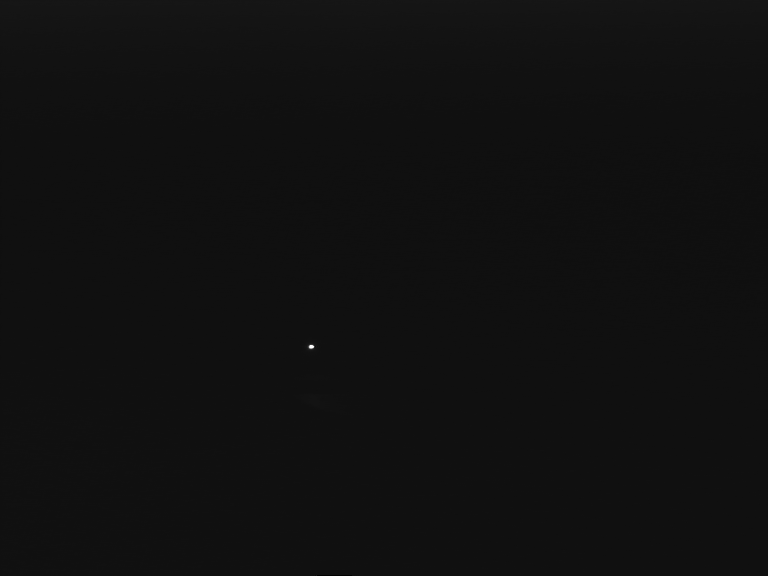

Supplement: S1 Dataset — This archive contains the captured data files used as the basis for the P4P solutions described in the manuscript. The data are provided in a directory hierarchy where each degree of freedom has a separate directory. And the calibration data is the captured data used in the camera calibration. (ZIP) [file pone.0134029.s001.zip › S1_Dataset/Pitch Angle/(35,0,0,1).tif]

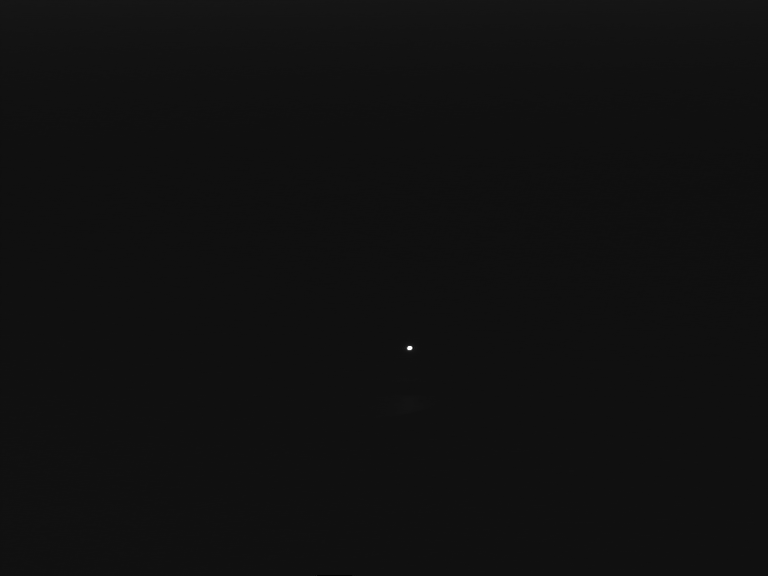

Supplement: S1 Dataset — This archive contains the captured data files used as the basis for the P4P solutions described in the manuscript. The data are provided in a directory hierarchy where each degree of freedom has a separate directory. And the calibration data is the captured data used in the camera calibration. (ZIP) [file pone.0134029.s001.zip › S1_Dataset/Pitch Angle/(35,0,0,2).tif]

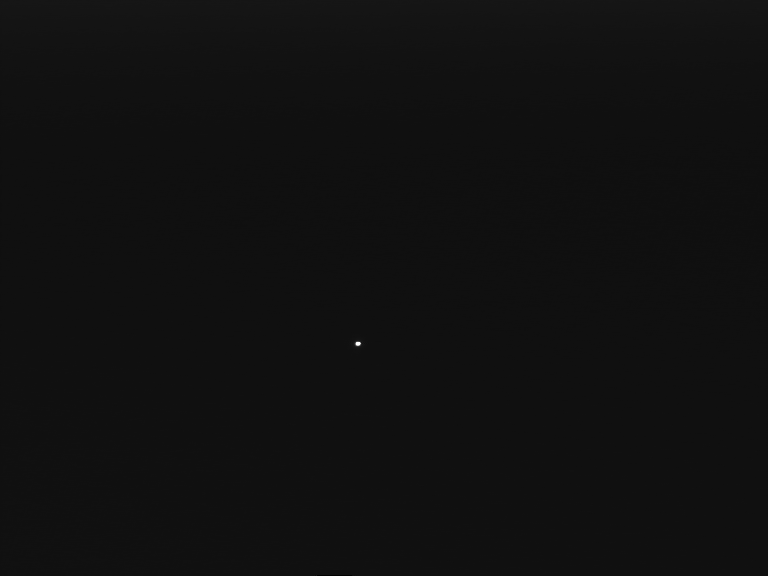

Supplement: S1 Dataset — This archive contains the captured data files used as the basis for the P4P solutions described in the manuscript. The data are provided in a directory hierarchy where each degree of freedom has a separate directory. And the calibration data is the captured data used in the camera calibration. (ZIP) [file pone.0134029.s001.zip › S1_Dataset/Pitch Angle/(35,0,0,3).tif]

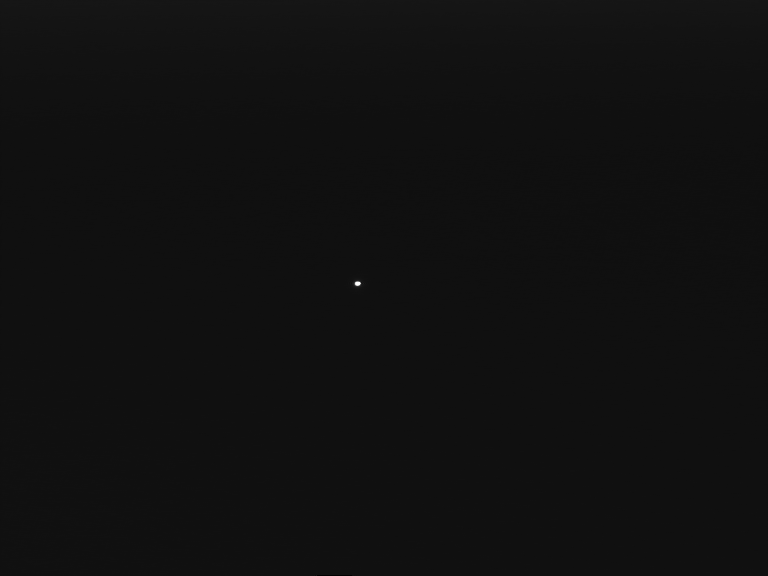

Supplement: S1 Dataset — This archive contains the captured data files used as the basis for the P4P solutions described in the manuscript. The data are provided in a directory hierarchy where each degree of freedom has a separate directory. And the calibration data is the captured data used in the camera calibration. (ZIP) [file pone.0134029.s001.zip › S1_Dataset/Pitch Angle/(36,0,0,0).tif]

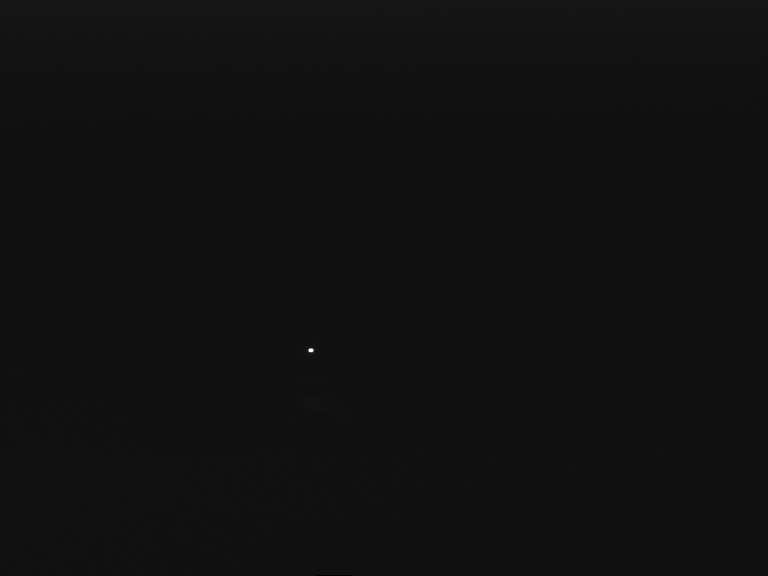

Supplement: S1 Dataset — This archive contains the captured data files used as the basis for the P4P solutions described in the manuscript. The data are provided in a directory hierarchy where each degree of freedom has a separate directory. And the calibration data is the captured data used in the camera calibration. (ZIP) [file pone.0134029.s001.zip › S1_Dataset/Pitch Angle/(36,0,0,1).tif]

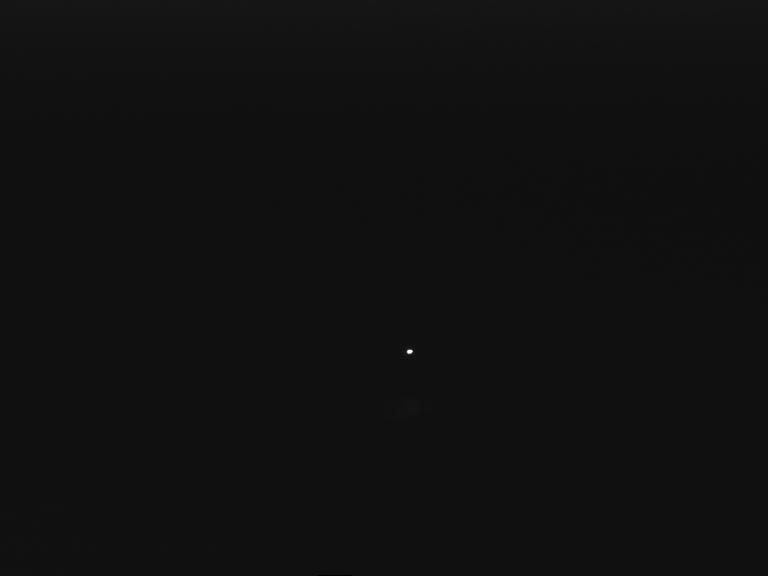

Supplement: S1 Dataset — This archive contains the captured data files used as the basis for the P4P solutions described in the manuscript. The data are provided in a directory hierarchy where each degree of freedom has a separate directory. And the calibration data is the captured data used in the camera calibration. (ZIP) [file pone.0134029.s001.zip › S1_Dataset/Pitch Angle/(36,0,0,2).tif]

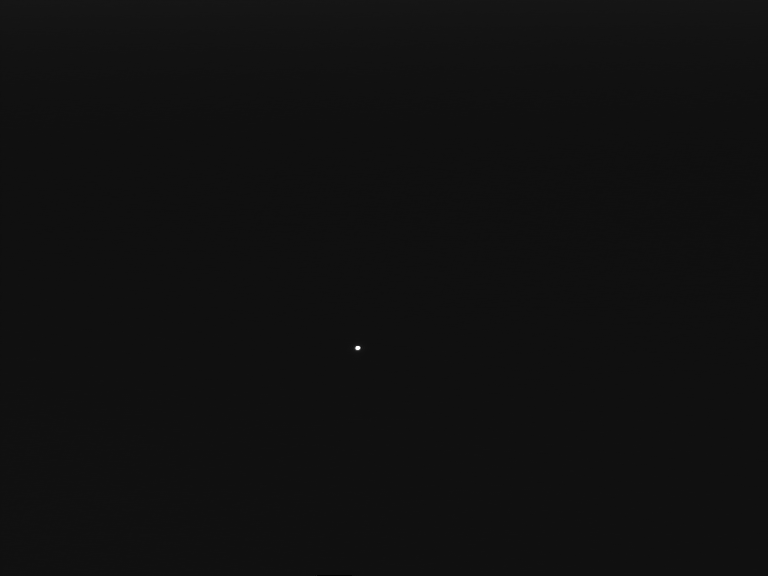

Supplement: S1 Dataset — This archive contains the captured data files used as the basis for the P4P solutions described in the manuscript. The data are provided in a directory hierarchy where each degree of freedom has a separate directory. And the calibration data is the captured data used in the camera calibration. (ZIP) [file pone.0134029.s001.zip › S1_Dataset/Pitch Angle/(36,0,0,3).tif]

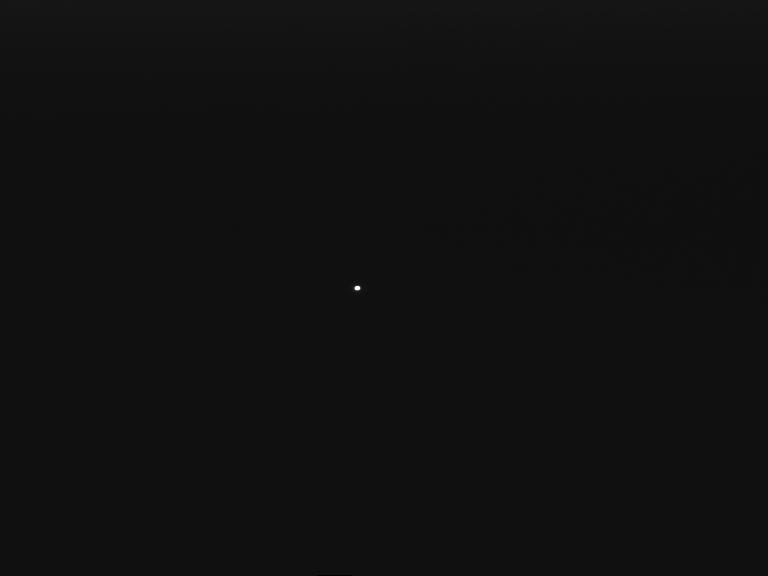

Supplement: S1 Dataset — This archive contains the captured data files used as the basis for the P4P solutions described in the manuscript. The data are provided in a directory hierarchy where each degree of freedom has a separate directory. And the calibration data is the captured data used in the camera calibration. (ZIP) [file pone.0134029.s001.zip › S1_Dataset/Pitch Angle/(37,0,0,0).tif]

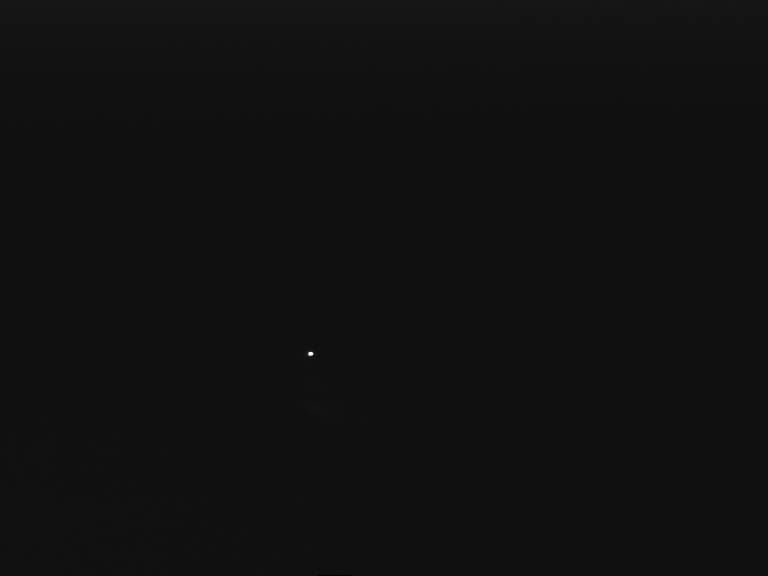

Supplement: S1 Dataset — This archive contains the captured data files used as the basis for the P4P solutions described in the manuscript. The data are provided in a directory hierarchy where each degree of freedom has a separate directory. And the calibration data is the captured data used in the camera calibration. (ZIP) [file pone.0134029.s001.zip › S1_Dataset/Pitch Angle/(37,0,0,1).tif]

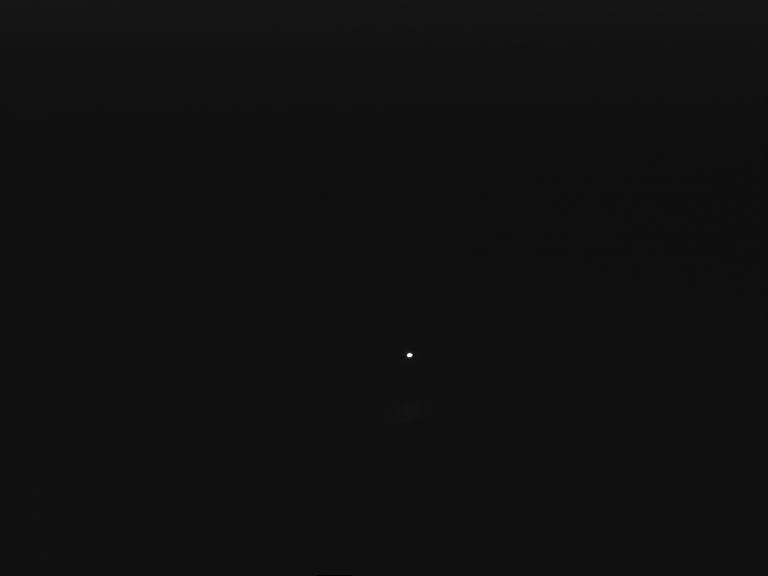

Supplement: S1 Dataset — This archive contains the captured data files used as the basis for the P4P solutions described in the manuscript. The data are provided in a directory hierarchy where each degree of freedom has a separate directory. And the calibration data is the captured data used in the camera calibration. (ZIP) [file pone.0134029.s001.zip › S1_Dataset/Pitch Angle/(37,0,0,2).tif]

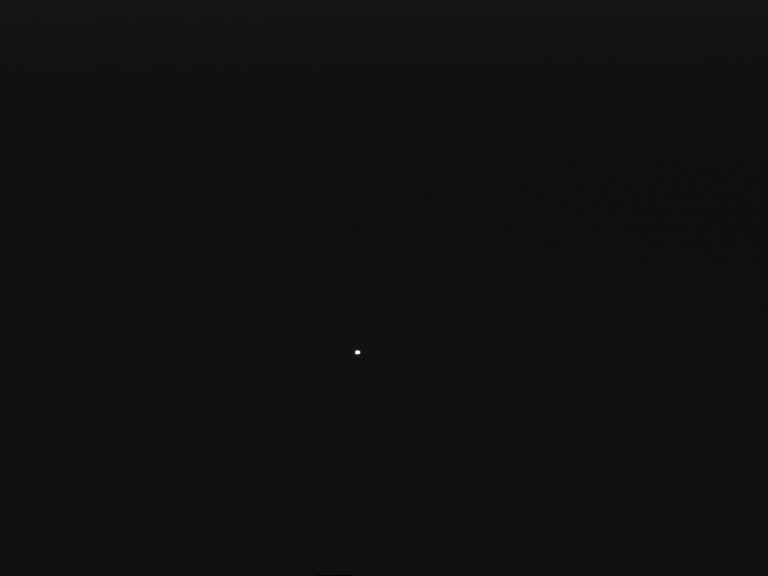

Supplement: S1 Dataset — This archive contains the captured data files used as the basis for the P4P solutions described in the manuscript. The data are provided in a directory hierarchy where each degree of freedom has a separate directory. And the calibration data is the captured data used in the camera calibration. (ZIP) [file pone.0134029.s001.zip › S1_Dataset/Pitch Angle/(37,0,0,3).tif]

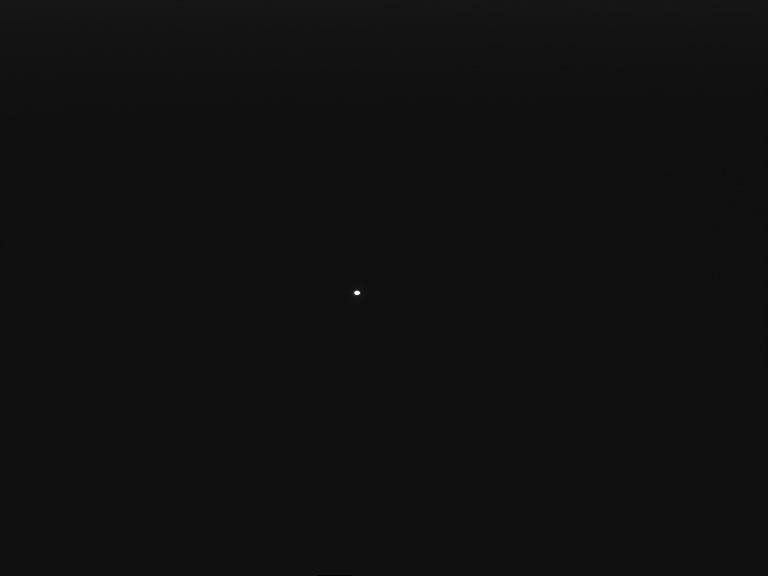

Supplement: S1 Dataset — This archive contains the captured data files used as the basis for the P4P solutions described in the manuscript. The data are provided in a directory hierarchy where each degree of freedom has a separate directory. And the calibration data is the captured data used in the camera calibration. (ZIP) [file pone.0134029.s001.zip › S1_Dataset/Pitch Angle/(38,0,0,0).tif]

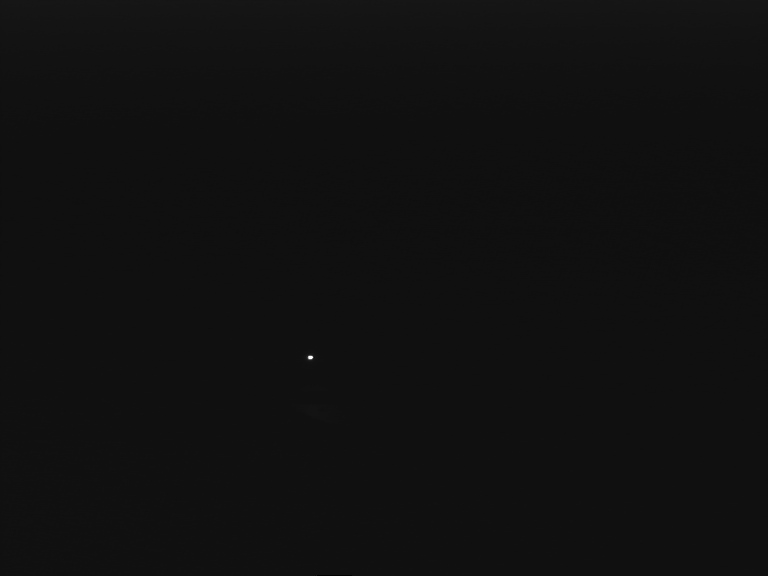

Supplement: S1 Dataset — This archive contains the captured data files used as the basis for the P4P solutions described in the manuscript. The data are provided in a directory hierarchy where each degree of freedom has a separate directory. And the calibration data is the captured data used in the camera calibration. (ZIP) [file pone.0134029.s001.zip › S1_Dataset/Pitch Angle/(38,0,0,1).tif]

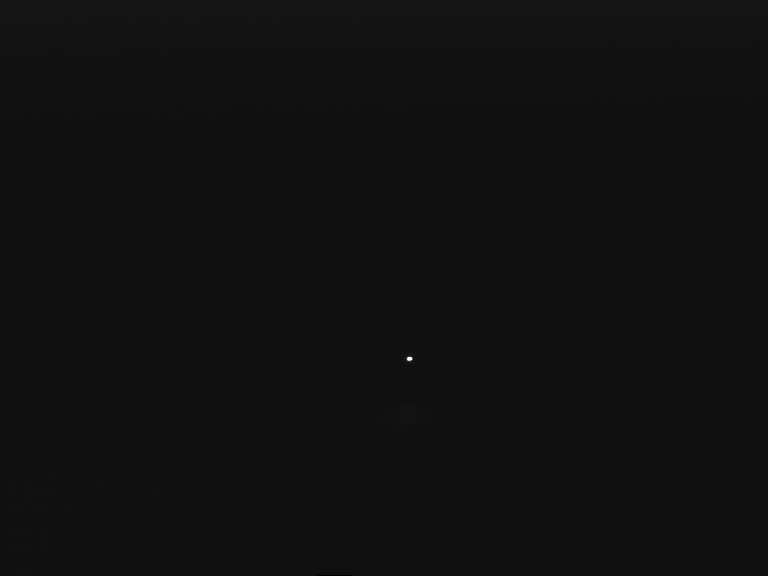

Supplement: S1 Dataset — This archive contains the captured data files used as the basis for the P4P solutions described in the manuscript. The data are provided in a directory hierarchy where each degree of freedom has a separate directory. And the calibration data is the captured data used in the camera calibration. (ZIP) [file pone.0134029.s001.zip › S1_Dataset/Pitch Angle/(38,0,0,2).tif]

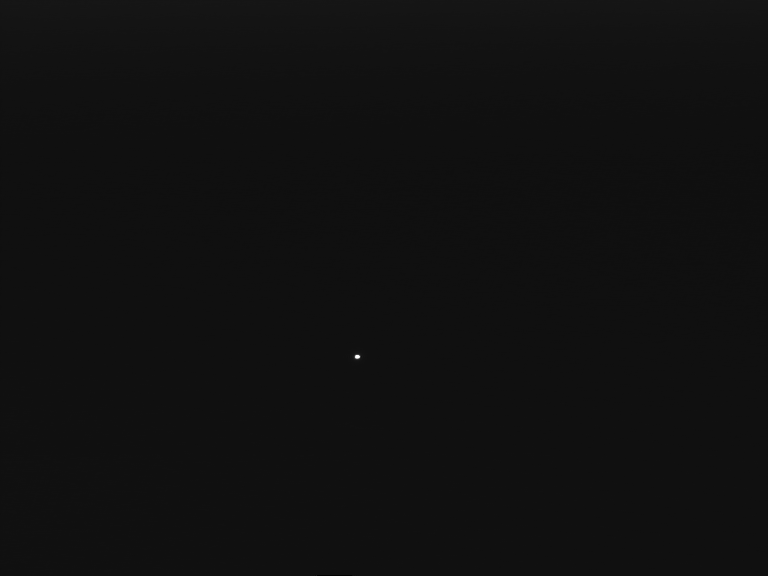

Supplement: S1 Dataset — This archive contains the captured data files used as the basis for the P4P solutions described in the manuscript. The data are provided in a directory hierarchy where each degree of freedom has a separate directory. And the calibration data is the captured data used in the camera calibration. (ZIP) [file pone.0134029.s001.zip › S1_Dataset/Pitch Angle/(38,0,0,3).tif]

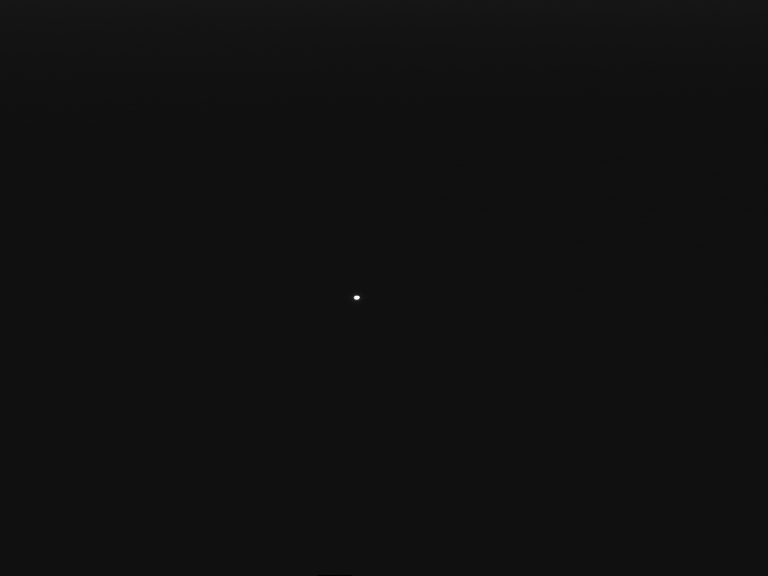

Supplement: S1 Dataset — This archive contains the captured data files used as the basis for the P4P solutions described in the manuscript. The data are provided in a directory hierarchy where each degree of freedom has a separate directory. And the calibration data is the captured data used in the camera calibration. (ZIP) [file pone.0134029.s001.zip › S1_Dataset/Pitch Angle/(39,0,0,0).tif]

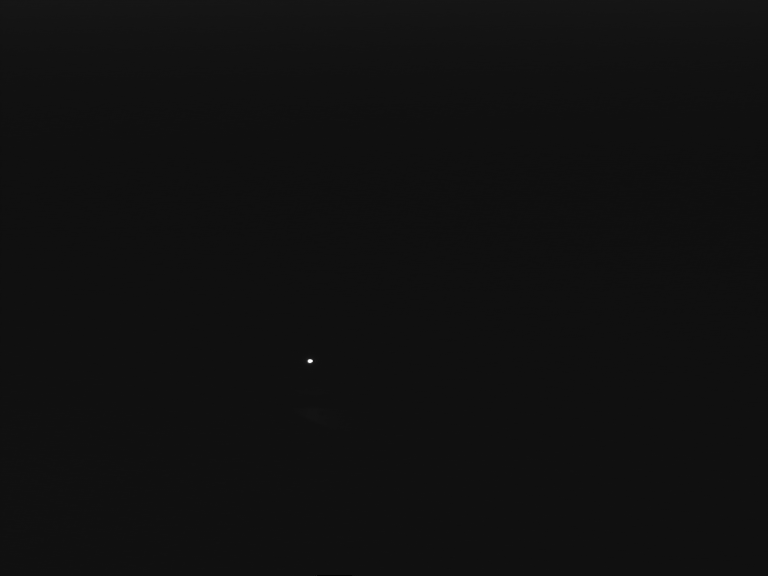

Supplement: S1 Dataset — This archive contains the captured data files used as the basis for the P4P solutions described in the manuscript. The data are provided in a directory hierarchy where each degree of freedom has a separate directory. And the calibration data is the captured data used in the camera calibration. (ZIP) [file pone.0134029.s001.zip › S1_Dataset/Pitch Angle/(39,0,0,1).tif]

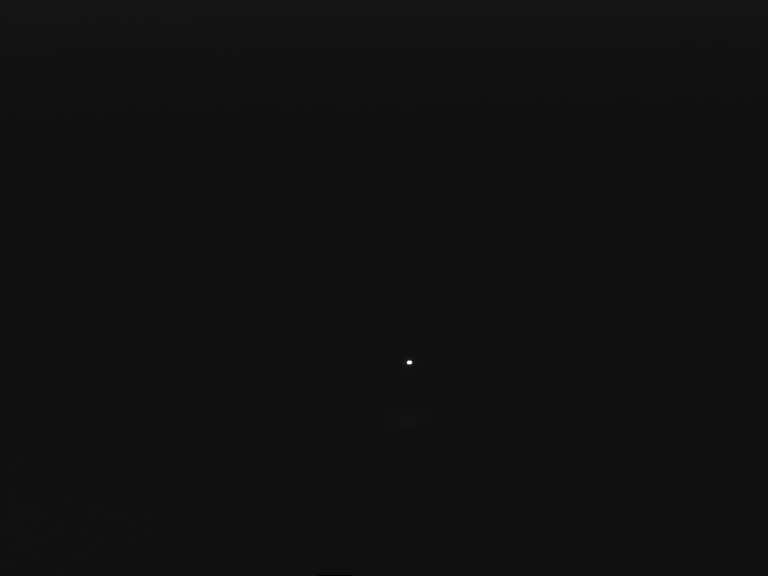

Supplement: S1 Dataset — This archive contains the captured data files used as the basis for the P4P solutions described in the manuscript. The data are provided in a directory hierarchy where each degree of freedom has a separate directory. And the calibration data is the captured data used in the camera calibration. (ZIP) [file pone.0134029.s001.zip › S1_Dataset/Pitch Angle/(39,0,0,2).tif]

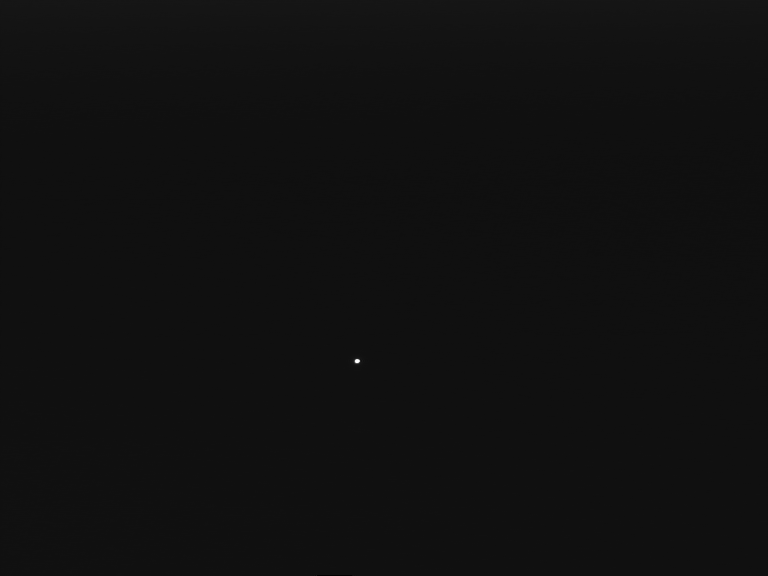

Supplement: S1 Dataset — This archive contains the captured data files used as the basis for the P4P solutions described in the manuscript. The data are provided in a directory hierarchy where each degree of freedom has a separate directory. And the calibration data is the captured data used in the camera calibration. (ZIP) [file pone.0134029.s001.zip › S1_Dataset/Pitch Angle/(39,0,0,3).tif]

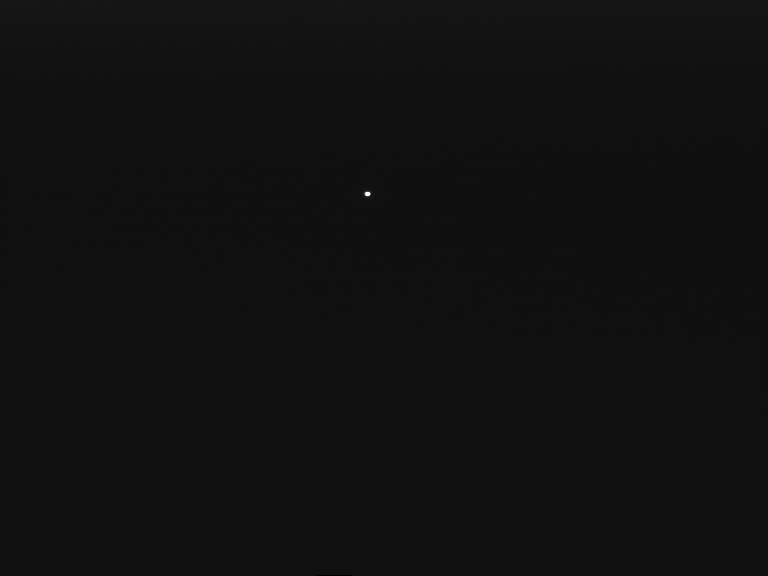

Supplement: S1 Dataset — This archive contains the captured data files used as the basis for the P4P solutions described in the manuscript. The data are provided in a directory hierarchy where each degree of freedom has a separate directory. And the calibration data is the captured data used in the camera calibration. (ZIP) [file pone.0134029.s001.zip › S1_Dataset/Pitch Angle/(4,0,0,0).tif]

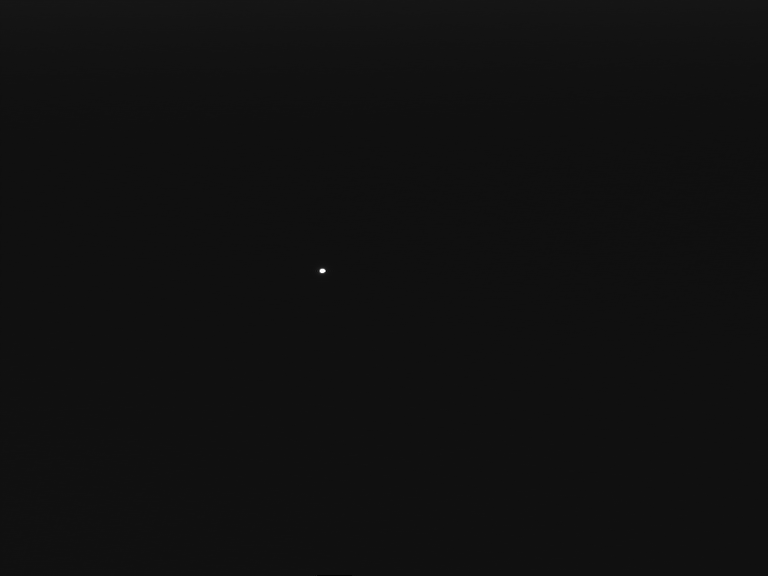

Supplement: S1 Dataset — This archive contains the captured data files used as the basis for the P4P solutions described in the manuscript. The data are provided in a directory hierarchy where each degree of freedom has a separate directory. And the calibration data is the captured data used in the camera calibration. (ZIP) [file pone.0134029.s001.zip › S1_Dataset/Pitch Angle/(4,0,0,1).tif]

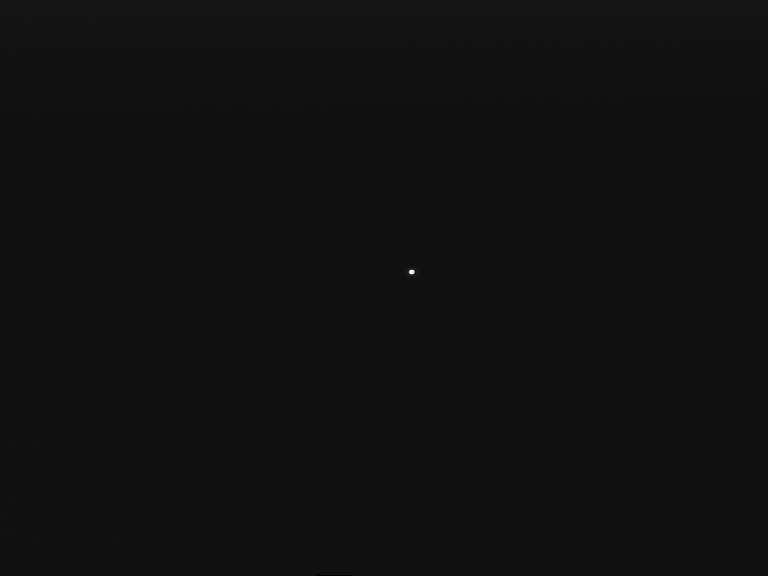

Supplement: S1 Dataset — This archive contains the captured data files used as the basis for the P4P solutions described in the manuscript. The data are provided in a directory hierarchy where each degree of freedom has a separate directory. And the calibration data is the captured data used in the camera calibration. (ZIP) [file pone.0134029.s001.zip › S1_Dataset/Pitch Angle/(4,0,0,2).tif]

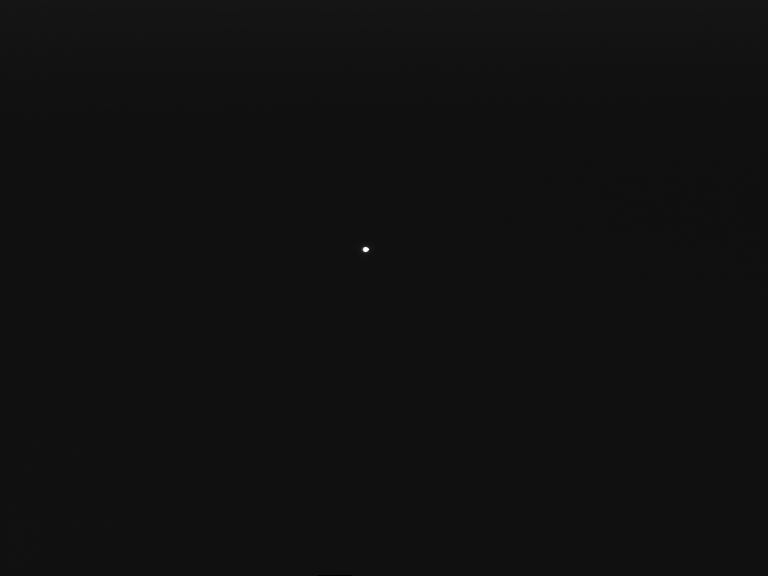

Supplement: S1 Dataset — This archive contains the captured data files used as the basis for the P4P solutions described in the manuscript. The data are provided in a directory hierarchy where each degree of freedom has a separate directory. And the calibration data is the captured data used in the camera calibration. (ZIP) [file pone.0134029.s001.zip › S1_Dataset/Pitch Angle/(4,0,0,3).tif]

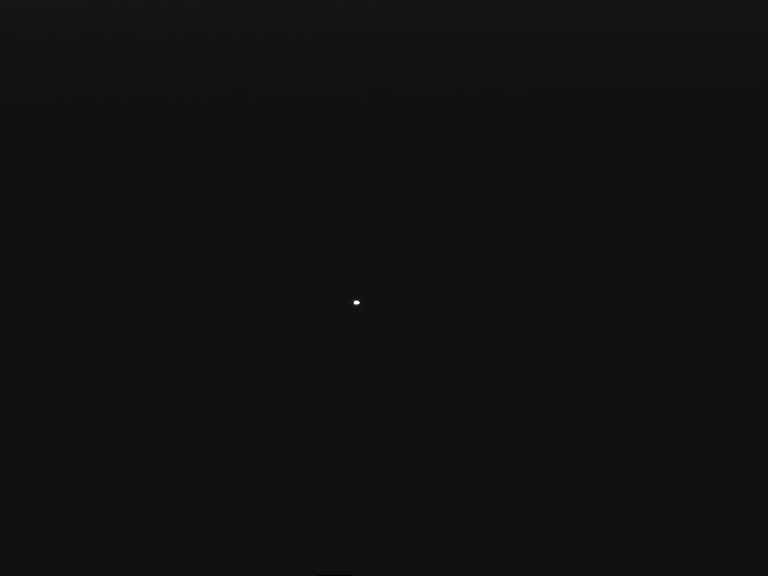

Supplement: S1 Dataset — This archive contains the captured data files used as the basis for the P4P solutions described in the manuscript. The data are provided in a directory hierarchy where each degree of freedom has a separate directory. And the calibration data is the captured data used in the camera calibration. (ZIP) [file pone.0134029.s001.zip › S1_Dataset/Pitch Angle/(40,0,0,0).tif]

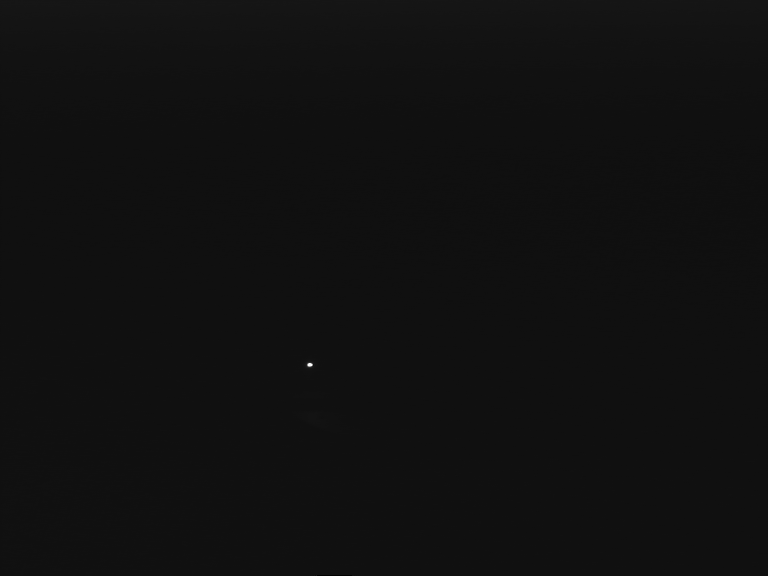

Supplement: S1 Dataset — This archive contains the captured data files used as the basis for the P4P solutions described in the manuscript. The data are provided in a directory hierarchy where each degree of freedom has a separate directory. And the calibration data is the captured data used in the camera calibration. (ZIP) [file pone.0134029.s001.zip › S1_Dataset/Pitch Angle/(40,0,0,1).tif]

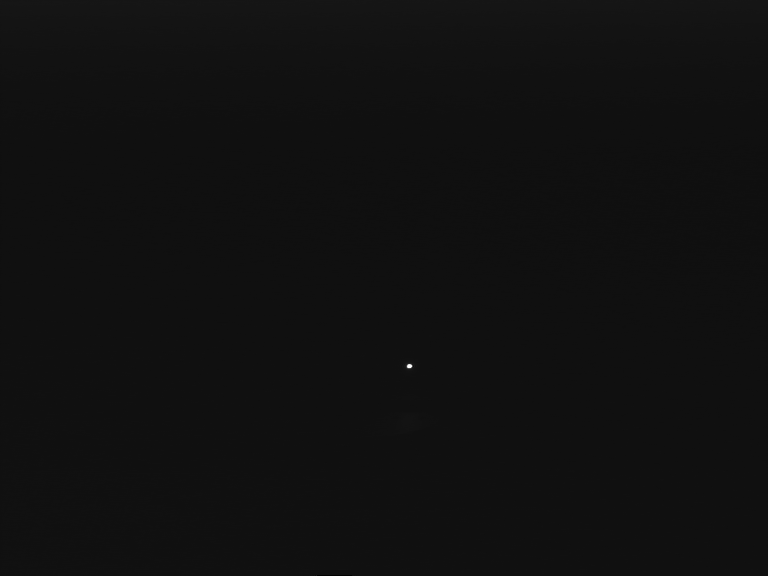

Supplement: S1 Dataset — This archive contains the captured data files used as the basis for the P4P solutions described in the manuscript. The data are provided in a directory hierarchy where each degree of freedom has a separate directory. And the calibration data is the captured data used in the camera calibration. (ZIP) [file pone.0134029.s001.zip › S1_Dataset/Pitch Angle/(40,0,0,2).tif]

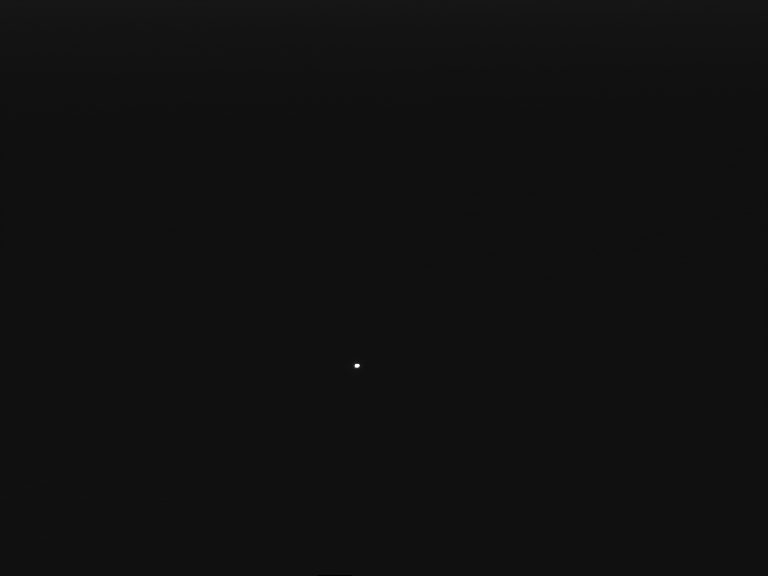

Supplement: S1 Dataset — This archive contains the captured data files used as the basis for the P4P solutions described in the manuscript. The data are provided in a directory hierarchy where each degree of freedom has a separate directory. And the calibration data is the captured data used in the camera calibration. (ZIP) [file pone.0134029.s001.zip › S1_Dataset/Pitch Angle/(40,0,0,3).tif]

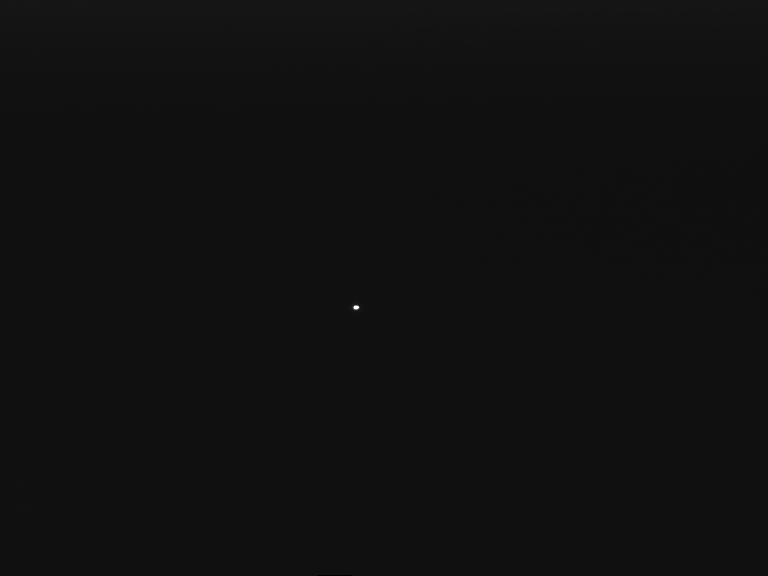

Supplement: S1 Dataset — This archive contains the captured data files used as the basis for the P4P solutions described in the manuscript. The data are provided in a directory hierarchy where each degree of freedom has a separate directory. And the calibration data is the captured data used in the camera calibration. (ZIP) [file pone.0134029.s001.zip › S1_Dataset/Pitch Angle/(41,0,0,0).tif]

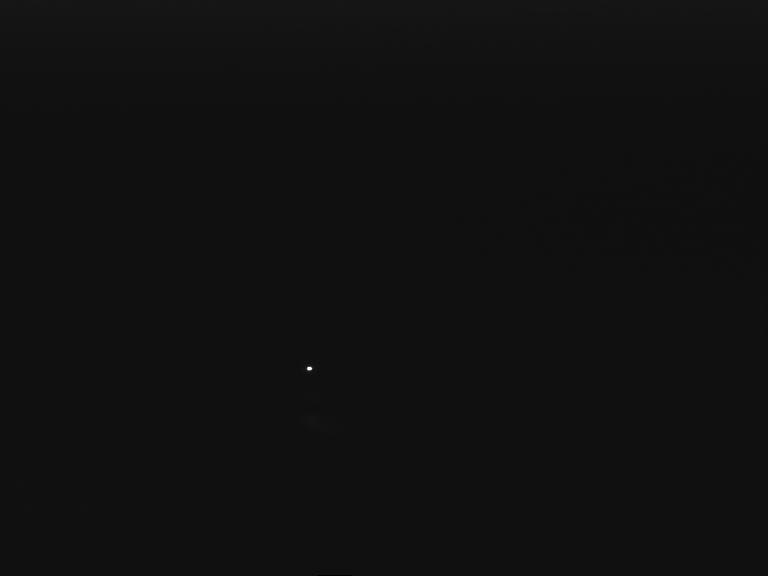

Supplement: S1 Dataset — This archive contains the captured data files used as the basis for the P4P solutions described in the manuscript. The data are provided in a directory hierarchy where each degree of freedom has a separate directory. And the calibration data is the captured data used in the camera calibration. (ZIP) [file pone.0134029.s001.zip › S1_Dataset/Pitch Angle/(41,0,0,1).tif]

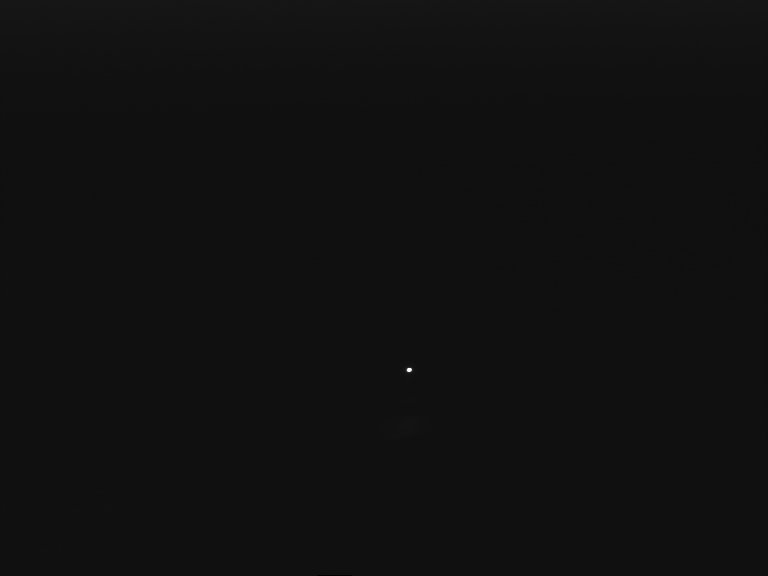

Supplement: S1 Dataset — This archive contains the captured data files used as the basis for the P4P solutions described in the manuscript. The data are provided in a directory hierarchy where each degree of freedom has a separate directory. And the calibration data is the captured data used in the camera calibration. (ZIP) [file pone.0134029.s001.zip › S1_Dataset/Pitch Angle/(41,0,0,2).tif]

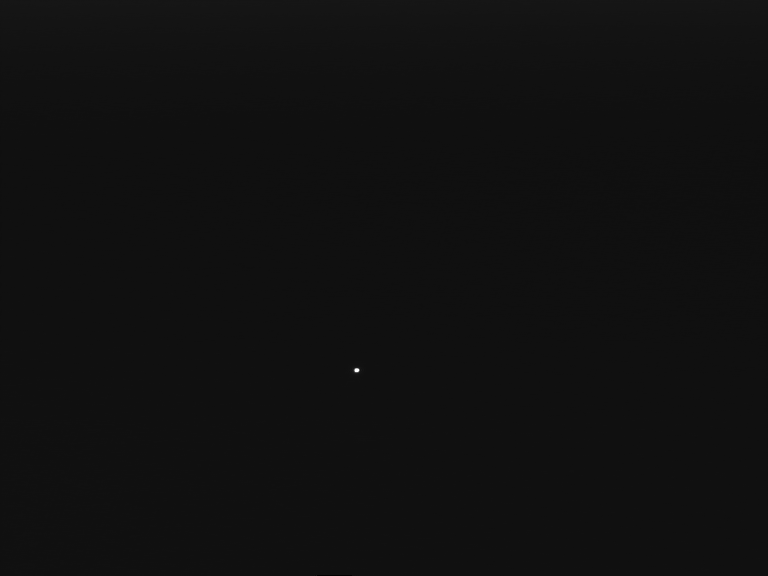

Supplement: S1 Dataset — This archive contains the captured data files used as the basis for the P4P solutions described in the manuscript. The data are provided in a directory hierarchy where each degree of freedom has a separate directory. And the calibration data is the captured data used in the camera calibration. (ZIP) [file pone.0134029.s001.zip › S1_Dataset/Pitch Angle/(41,0,0,3).tif]

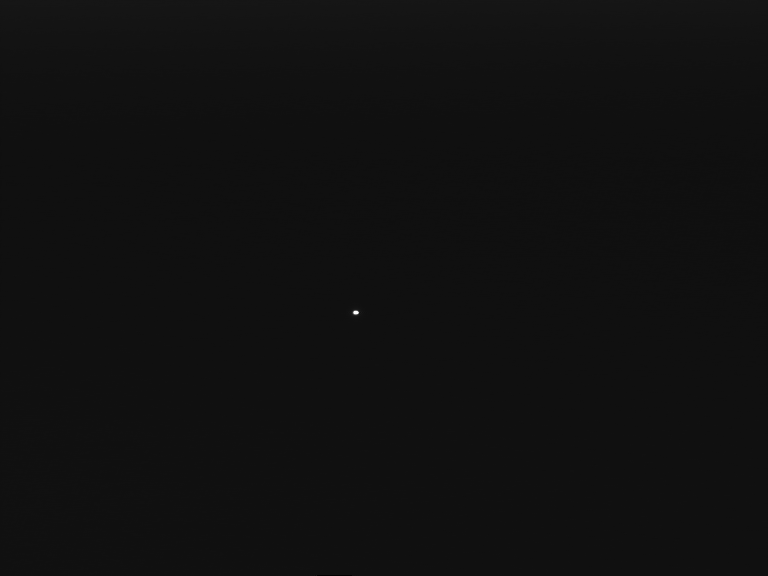

Supplement: S1 Dataset — This archive contains the captured data files used as the basis for the P4P solutions described in the manuscript. The data are provided in a directory hierarchy where each degree of freedom has a separate directory. And the calibration data is the captured data used in the camera calibration. (ZIP) [file pone.0134029.s001.zip › S1_Dataset/Pitch Angle/(42,0,0,0).tif]

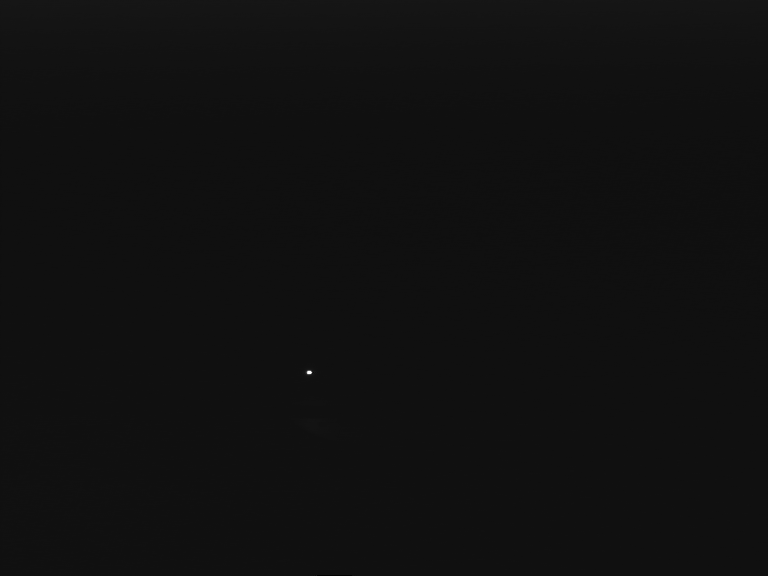

Supplement: S1 Dataset — This archive contains the captured data files used as the basis for the P4P solutions described in the manuscript. The data are provided in a directory hierarchy where each degree of freedom has a separate directory. And the calibration data is the captured data used in the camera calibration. (ZIP) [file pone.0134029.s001.zip › S1_Dataset/Pitch Angle/(42,0,0,1).tif]

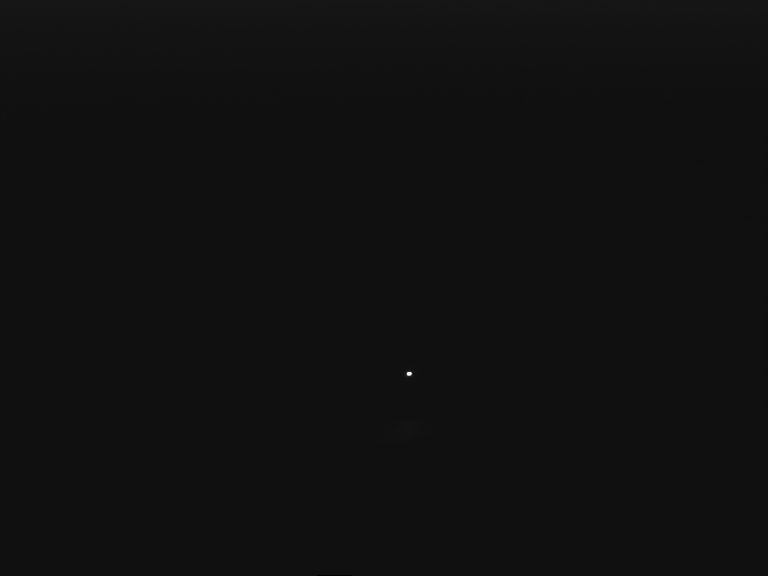

Supplement: S1 Dataset — This archive contains the captured data files used as the basis for the P4P solutions described in the manuscript. The data are provided in a directory hierarchy where each degree of freedom has a separate directory. And the calibration data is the captured data used in the camera calibration. (ZIP) [file pone.0134029.s001.zip › S1_Dataset/Pitch Angle/(42,0,0,2).tif]

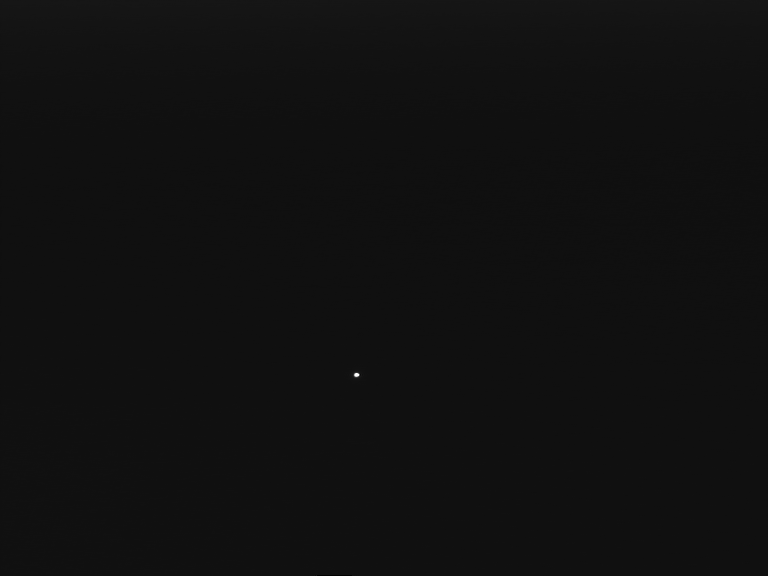

Supplement: S1 Dataset — This archive contains the captured data files used as the basis for the P4P solutions described in the manuscript. The data are provided in a directory hierarchy where each degree of freedom has a separate directory. And the calibration data is the captured data used in the camera calibration. (ZIP) [file pone.0134029.s001.zip › S1_Dataset/Pitch Angle/(42,0,0,3).tif]

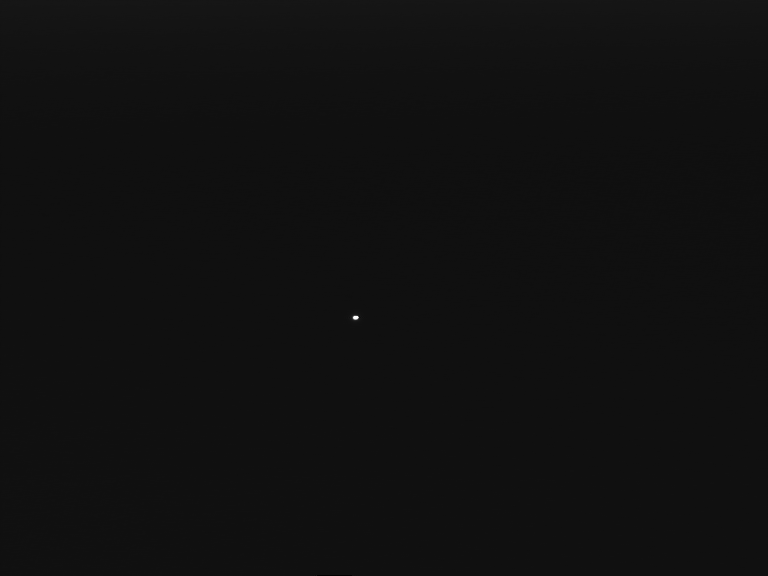

Supplement: S1 Dataset — This archive contains the captured data files used as the basis for the P4P solutions described in the manuscript. The data are provided in a directory hierarchy where each degree of freedom has a separate directory. And the calibration data is the captured data used in the camera calibration. (ZIP) [file pone.0134029.s001.zip › S1_Dataset/Pitch Angle/(43,0,0,0).tif]

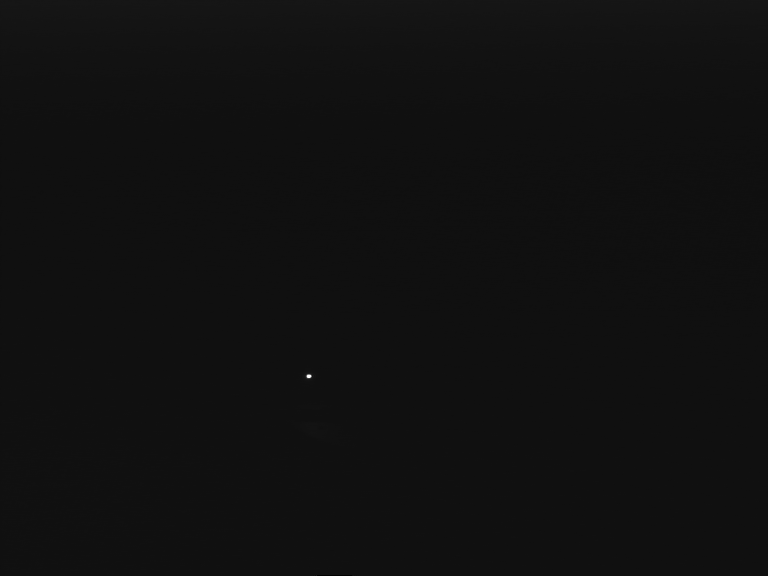

Supplement: S1 Dataset — This archive contains the captured data files used as the basis for the P4P solutions described in the manuscript. The data are provided in a directory hierarchy where each degree of freedom has a separate directory. And the calibration data is the captured data used in the camera calibration. (ZIP) [file pone.0134029.s001.zip › S1_Dataset/Pitch Angle/(43,0,0,1).tif]

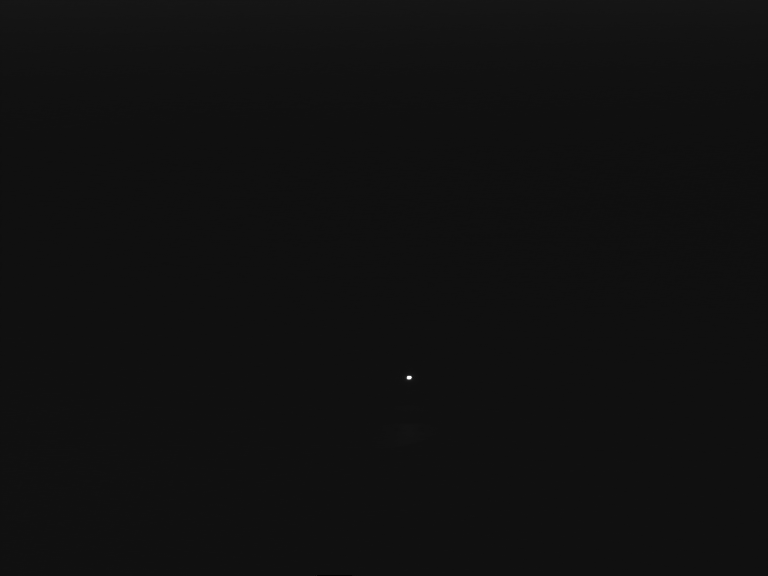

Supplement: S1 Dataset — This archive contains the captured data files used as the basis for the P4P solutions described in the manuscript. The data are provided in a directory hierarchy where each degree of freedom has a separate directory. And the calibration data is the captured data used in the camera calibration. (ZIP) [file pone.0134029.s001.zip › S1_Dataset/Pitch Angle/(43,0,0,2).tif]

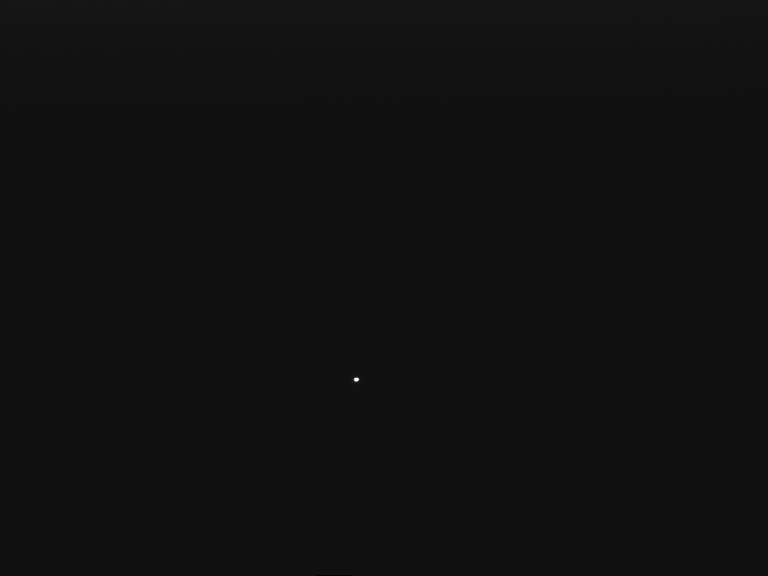

Supplement: S1 Dataset — This archive contains the captured data files used as the basis for the P4P solutions described in the manuscript. The data are provided in a directory hierarchy where each degree of freedom has a separate directory. And the calibration data is the captured data used in the camera calibration. (ZIP) [file pone.0134029.s001.zip › S1_Dataset/Pitch Angle/(43,0,0,3).tif]

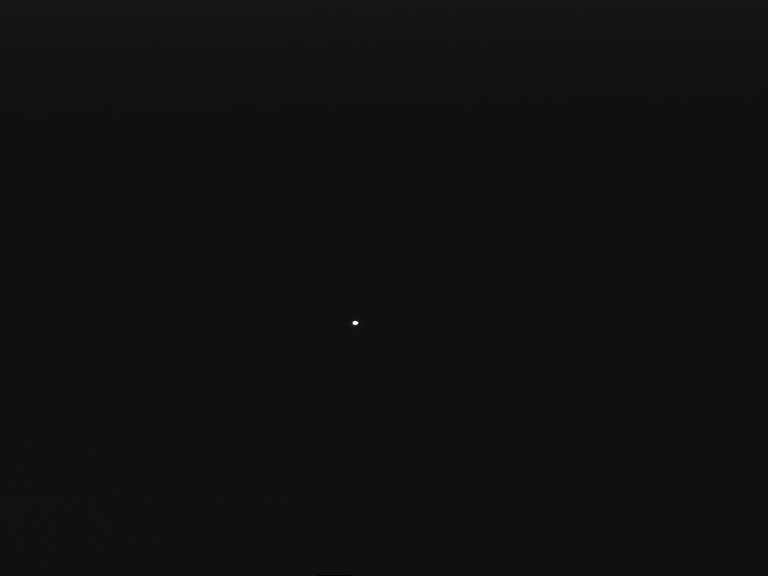

Supplement: S1 Dataset — This archive contains the captured data files used as the basis for the P4P solutions described in the manuscript. The data are provided in a directory hierarchy where each degree of freedom has a separate directory. And the calibration data is the captured data used in the camera calibration. (ZIP) [file pone.0134029.s001.zip › S1_Dataset/Pitch Angle/(44,0,0,0).tif]

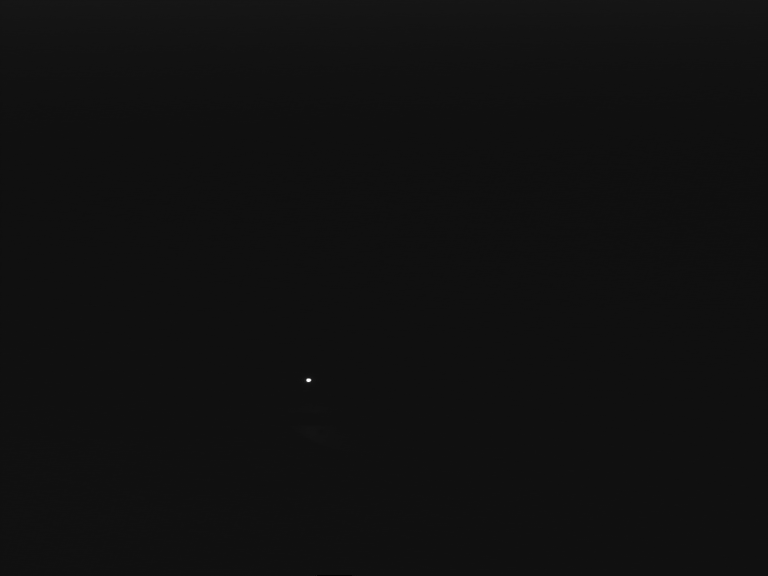

Supplement: S1 Dataset — This archive contains the captured data files used as the basis for the P4P solutions described in the manuscript. The data are provided in a directory hierarchy where each degree of freedom has a separate directory. And the calibration data is the captured data used in the camera calibration. (ZIP) [file pone.0134029.s001.zip › S1_Dataset/Pitch Angle/(44,0,0,1).tif]

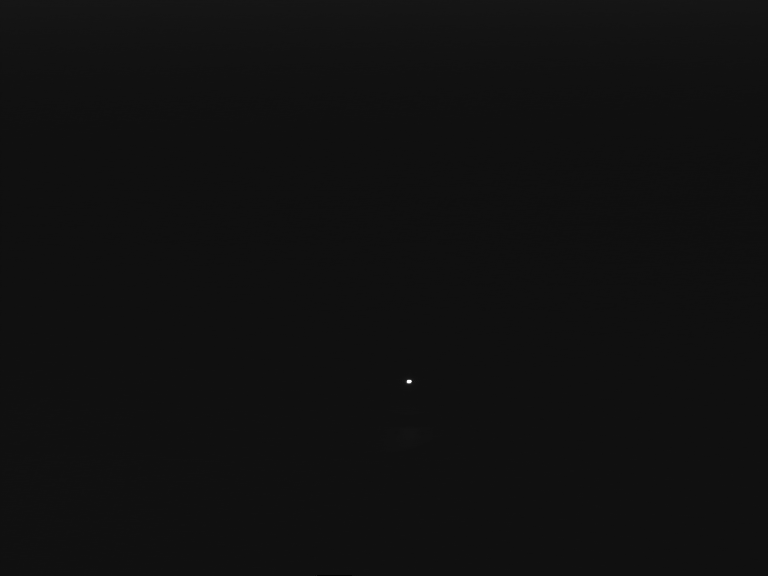

Supplement: S1 Dataset — This archive contains the captured data files used as the basis for the P4P solutions described in the manuscript. The data are provided in a directory hierarchy where each degree of freedom has a separate directory. And the calibration data is the captured data used in the camera calibration. (ZIP) [file pone.0134029.s001.zip › S1_Dataset/Pitch Angle/(44,0,0,2).tif]

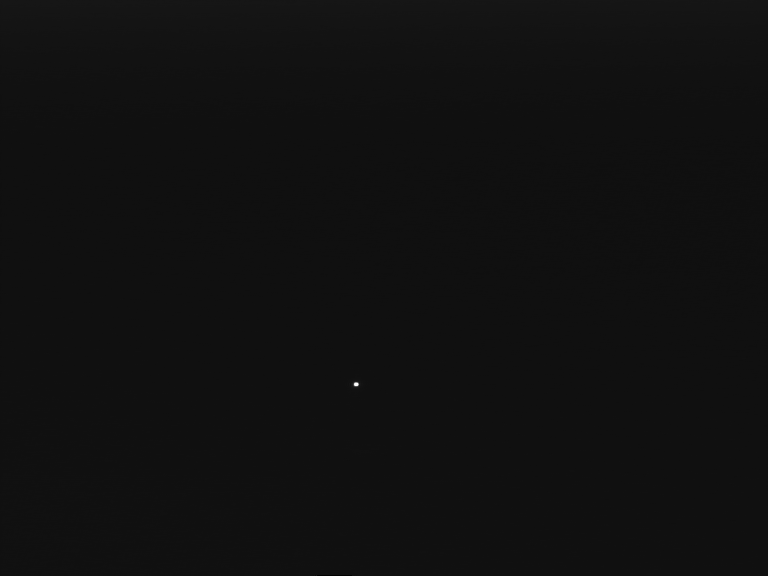

Supplement: S1 Dataset — This archive contains the captured data files used as the basis for the P4P solutions described in the manuscript. The data are provided in a directory hierarchy where each degree of freedom has a separate directory. And the calibration data is the captured data used in the camera calibration. (ZIP) [file pone.0134029.s001.zip › S1_Dataset/Pitch Angle/(44,0,0,3).tif]

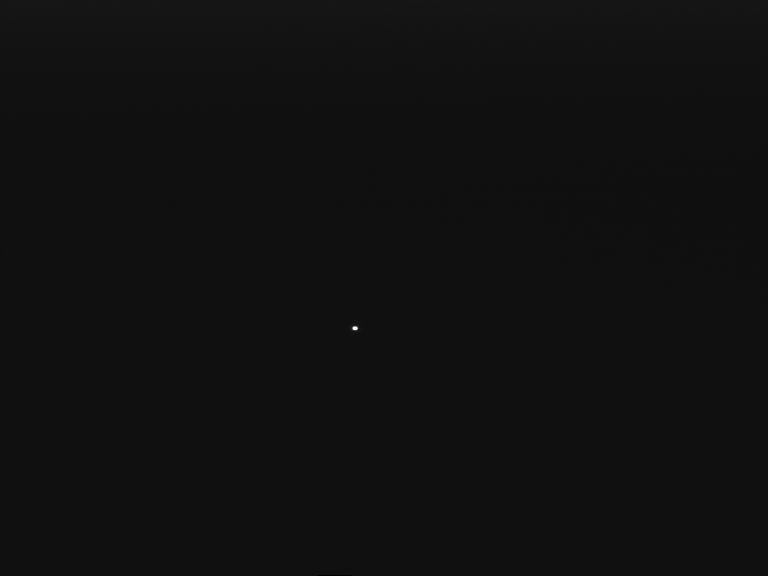

Supplement: S1 Dataset — This archive contains the captured data files used as the basis for the P4P solutions described in the manuscript. The data are provided in a directory hierarchy where each degree of freedom has a separate directory. And the calibration data is the captured data used in the camera calibration. (ZIP) [file pone.0134029.s001.zip › S1_Dataset/Pitch Angle/(45,0,0,0).tif]

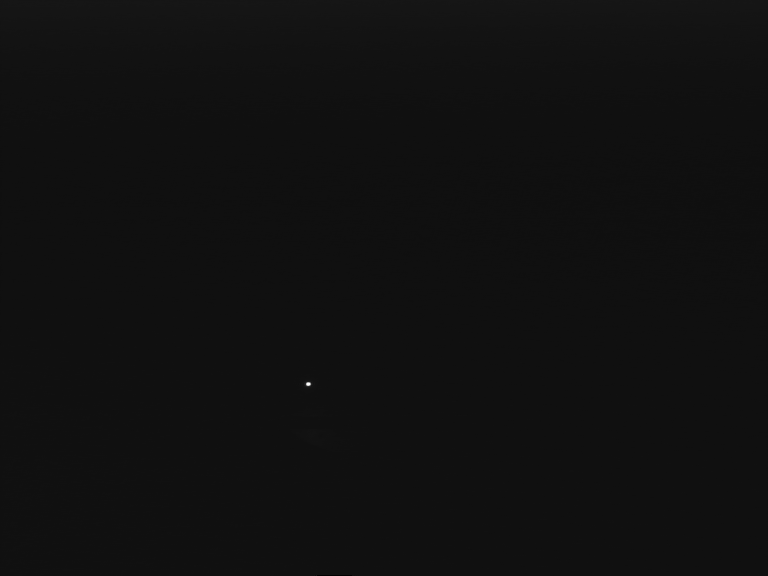

Supplement: S1 Dataset — This archive contains the captured data files used as the basis for the P4P solutions described in the manuscript. The data are provided in a directory hierarchy where each degree of freedom has a separate directory. And the calibration data is the captured data used in the camera calibration. (ZIP) [file pone.0134029.s001.zip › S1_Dataset/Pitch Angle/(45,0,0,1).tif]

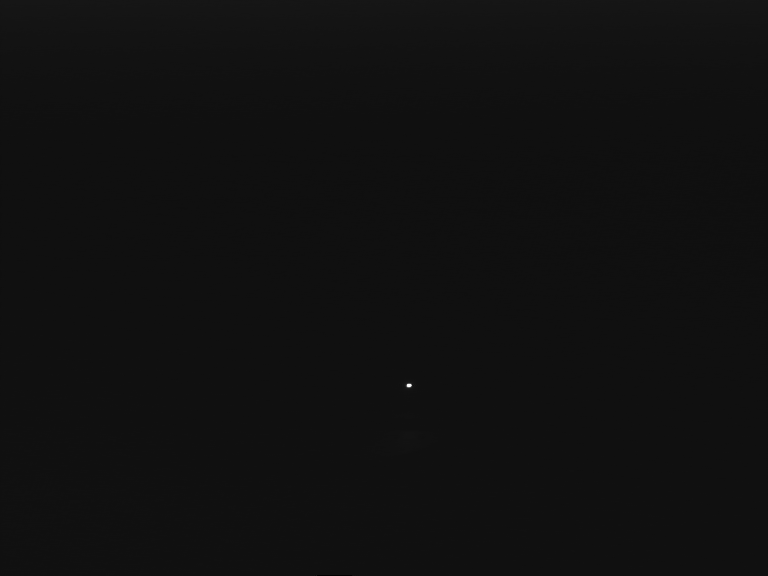

Supplement: S1 Dataset — This archive contains the captured data files used as the basis for the P4P solutions described in the manuscript. The data are provided in a directory hierarchy where each degree of freedom has a separate directory. And the calibration data is the captured data used in the camera calibration. (ZIP) [file pone.0134029.s001.zip › S1_Dataset/Pitch Angle/(45,0,0,2).tif]

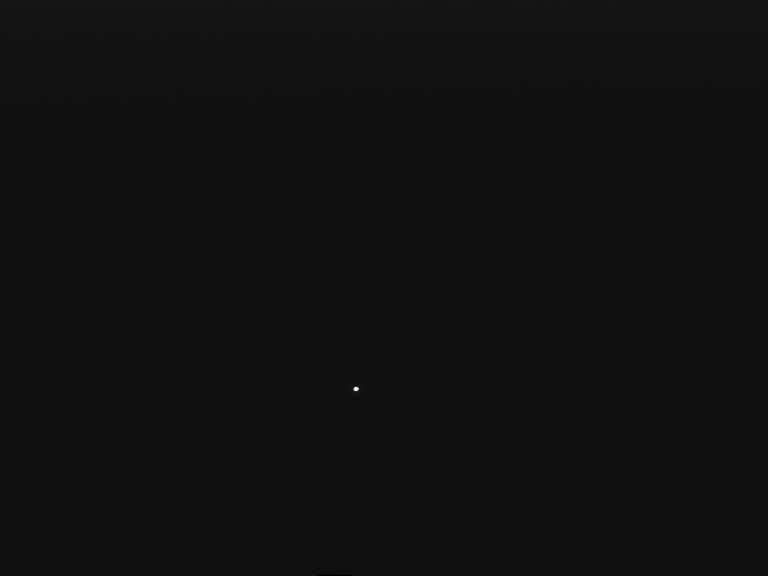

Supplement: S1 Dataset — This archive contains the captured data files used as the basis for the P4P solutions described in the manuscript. The data are provided in a directory hierarchy where each degree of freedom has a separate directory. And the calibration data is the captured data used in the camera calibration. (ZIP) [file pone.0134029.s001.zip › S1_Dataset/Pitch Angle/(45,0,0,3).tif]

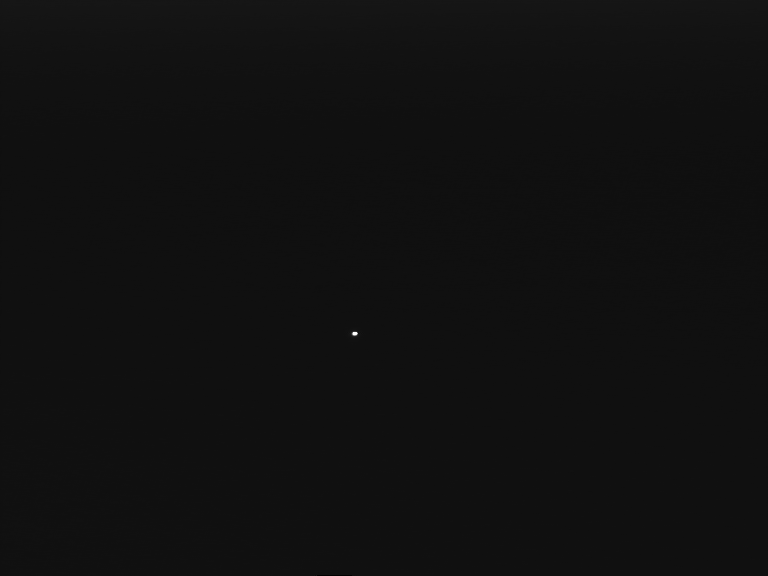

Supplement: S1 Dataset — This archive contains the captured data files used as the basis for the P4P solutions described in the manuscript. The data are provided in a directory hierarchy where each degree of freedom has a separate directory. And the calibration data is the captured data used in the camera calibration. (ZIP) [file pone.0134029.s001.zip › S1_Dataset/Pitch Angle/(46,0,0,0).tif]

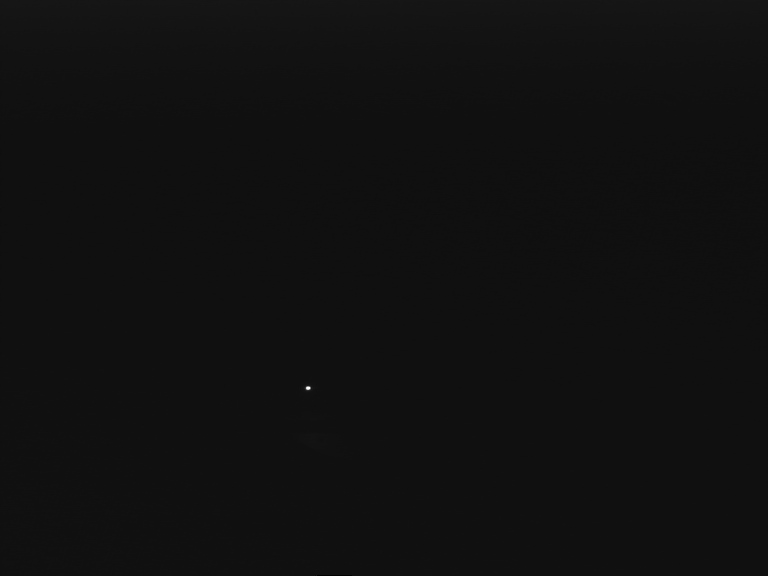

Supplement: S1 Dataset — This archive contains the captured data files used as the basis for the P4P solutions described in the manuscript. The data are provided in a directory hierarchy where each degree of freedom has a separate directory. And the calibration data is the captured data used in the camera calibration. (ZIP) [file pone.0134029.s001.zip › S1_Dataset/Pitch Angle/(46,0,0,1).tif]

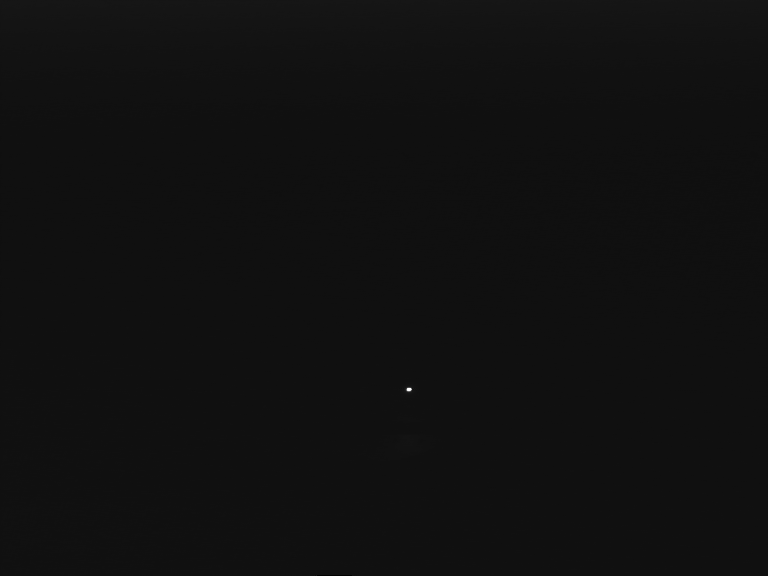

Supplement: S1 Dataset — This archive contains the captured data files used as the basis for the P4P solutions described in the manuscript. The data are provided in a directory hierarchy where each degree of freedom has a separate directory. And the calibration data is the captured data used in the camera calibration. (ZIP) [file pone.0134029.s001.zip › S1_Dataset/Pitch Angle/(46,0,0,2).tif]

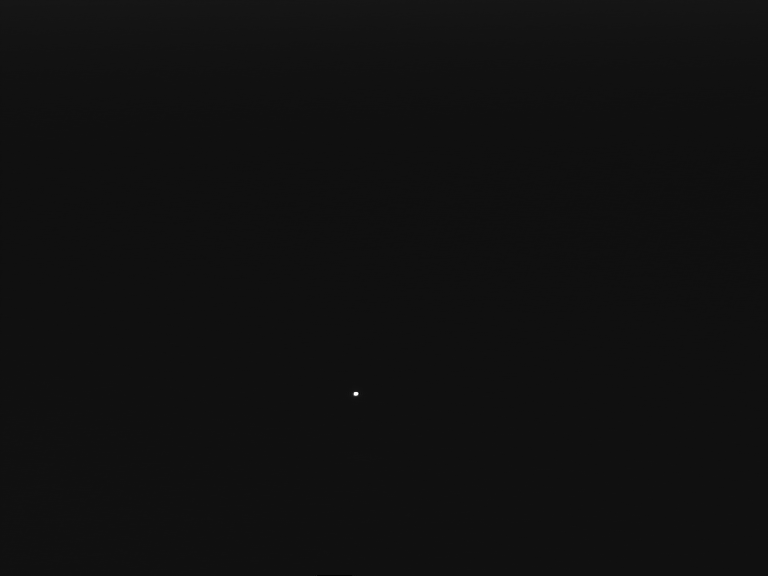

Supplement: S1 Dataset — This archive contains the captured data files used as the basis for the P4P solutions described in the manuscript. The data are provided in a directory hierarchy where each degree of freedom has a separate directory. And the calibration data is the captured data used in the camera calibration. (ZIP) [file pone.0134029.s001.zip › S1_Dataset/Pitch Angle/(46,0,0,3).tif]

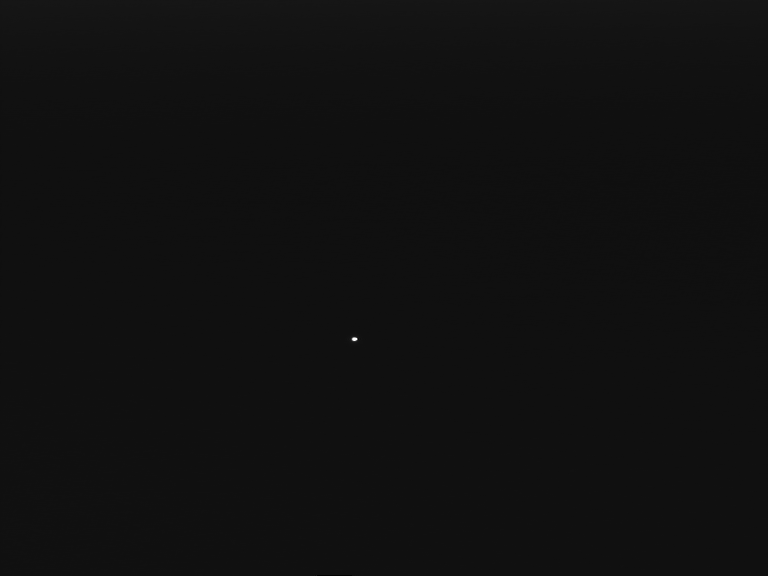

Supplement: S1 Dataset — This archive contains the captured data files used as the basis for the P4P solutions described in the manuscript. The data are provided in a directory hierarchy where each degree of freedom has a separate directory. And the calibration data is the captured data used in the camera calibration. (ZIP) [file pone.0134029.s001.zip › S1_Dataset/Pitch Angle/(47,0,0,0).tif]

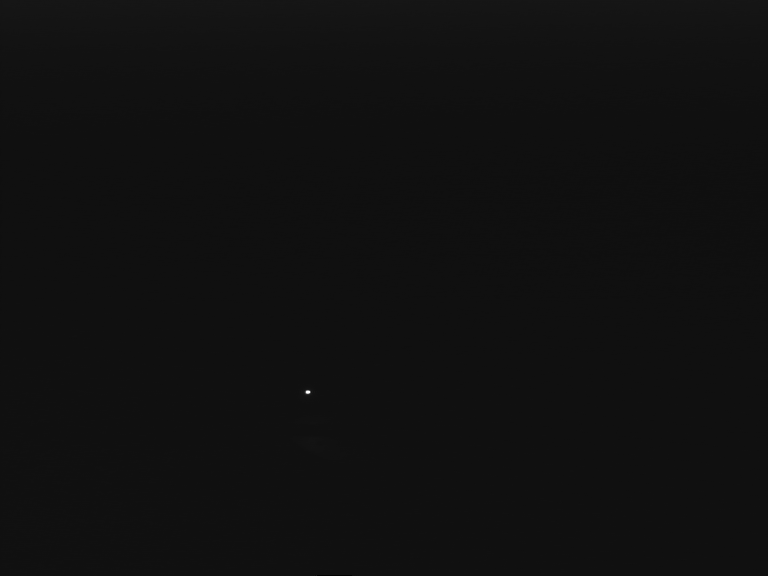

Supplement: S1 Dataset — This archive contains the captured data files used as the basis for the P4P solutions described in the manuscript. The data are provided in a directory hierarchy where each degree of freedom has a separate directory. And the calibration data is the captured data used in the camera calibration. (ZIP) [file pone.0134029.s001.zip › S1_Dataset/Pitch Angle/(47,0,0,1).tif]

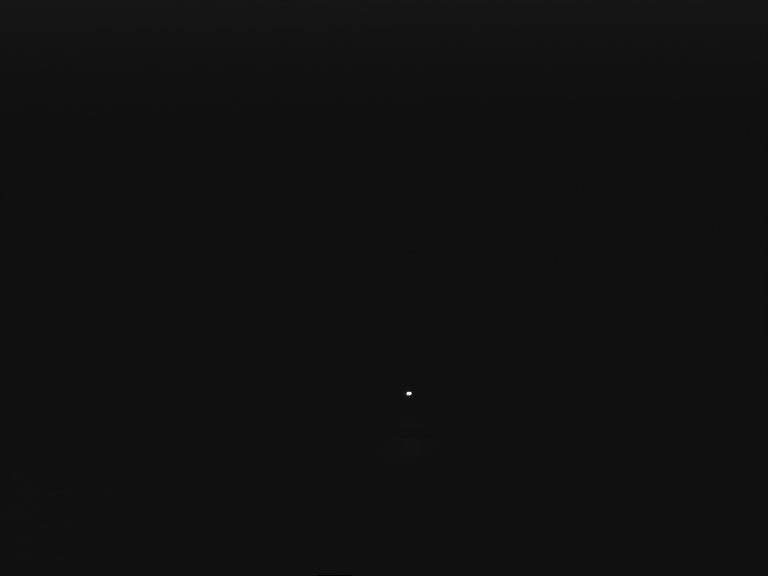

Supplement: S1 Dataset — This archive contains the captured data files used as the basis for the P4P solutions described in the manuscript. The data are provided in a directory hierarchy where each degree of freedom has a separate directory. And the calibration data is the captured data used in the camera calibration. (ZIP) [file pone.0134029.s001.zip › S1_Dataset/Pitch Angle/(47,0,0,2).tif]

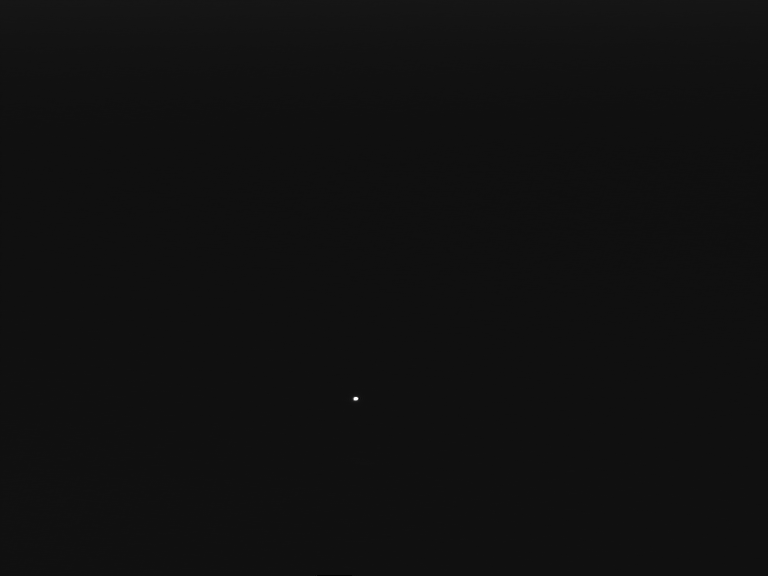

Supplement: S1 Dataset — This archive contains the captured data files used as the basis for the P4P solutions described in the manuscript. The data are provided in a directory hierarchy where each degree of freedom has a separate directory. And the calibration data is the captured data used in the camera calibration. (ZIP) [file pone.0134029.s001.zip › S1_Dataset/Pitch Angle/(47,0,0,3).tif]

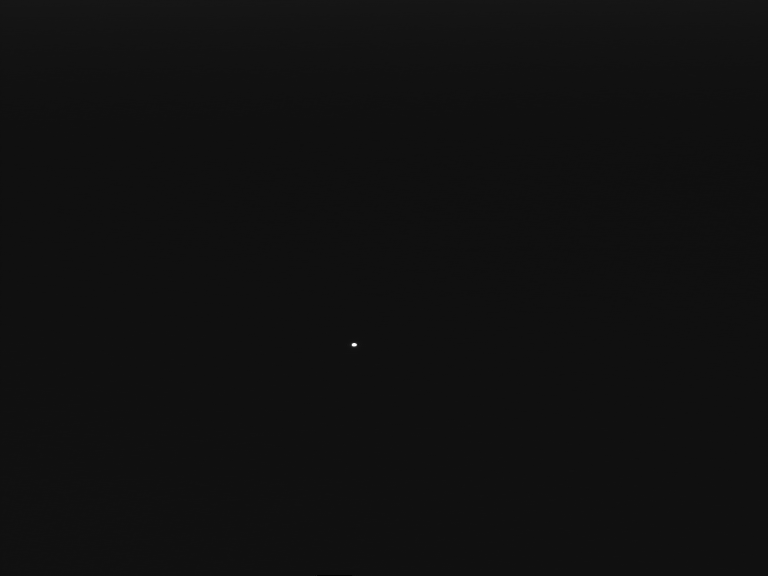

Supplement: S1 Dataset — This archive contains the captured data files used as the basis for the P4P solutions described in the manuscript. The data are provided in a directory hierarchy where each degree of freedom has a separate directory. And the calibration data is the captured data used in the camera calibration. (ZIP) [file pone.0134029.s001.zip › S1_Dataset/Pitch Angle/(48,0,0,0).tif]

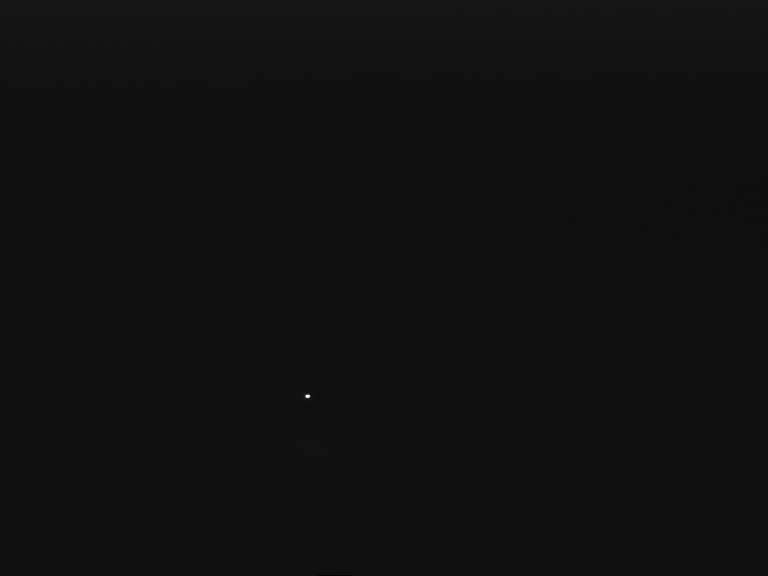

Supplement: S1 Dataset — This archive contains the captured data files used as the basis for the P4P solutions described in the manuscript. The data are provided in a directory hierarchy where each degree of freedom has a separate directory. And the calibration data is the captured data used in the camera calibration. (ZIP) [file pone.0134029.s001.zip › S1_Dataset/Pitch Angle/(48,0,0,1).tif]

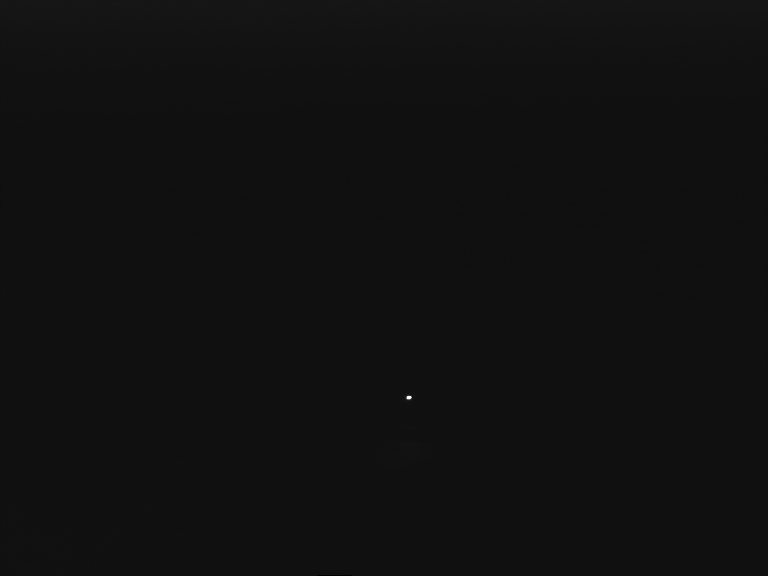

Supplement: S1 Dataset — This archive contains the captured data files used as the basis for the P4P solutions described in the manuscript. The data are provided in a directory hierarchy where each degree of freedom has a separate directory. And the calibration data is the captured data used in the camera calibration. (ZIP) [file pone.0134029.s001.zip › S1_Dataset/Pitch Angle/(48,0,0,2).tif]

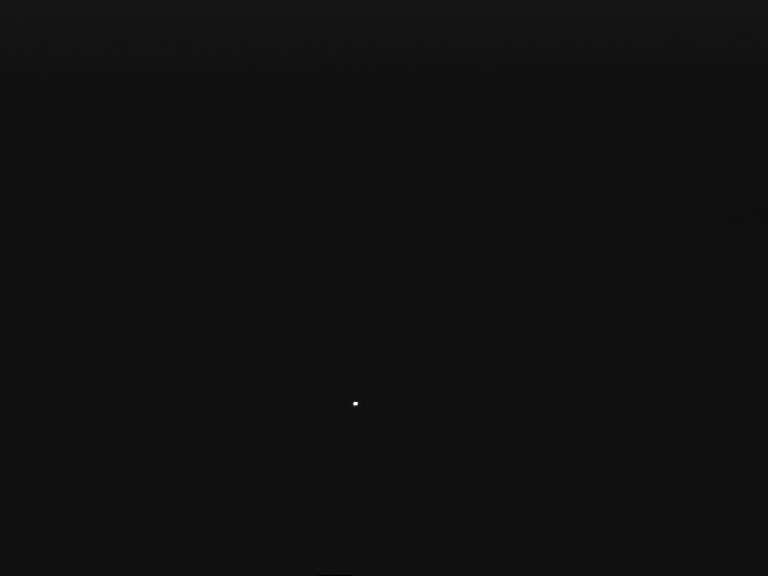

Supplement: S1 Dataset — This archive contains the captured data files used as the basis for the P4P solutions described in the manuscript. The data are provided in a directory hierarchy where each degree of freedom has a separate directory. And the calibration data is the captured data used in the camera calibration. (ZIP) [file pone.0134029.s001.zip › S1_Dataset/Pitch Angle/(48,0,0,3).tif]

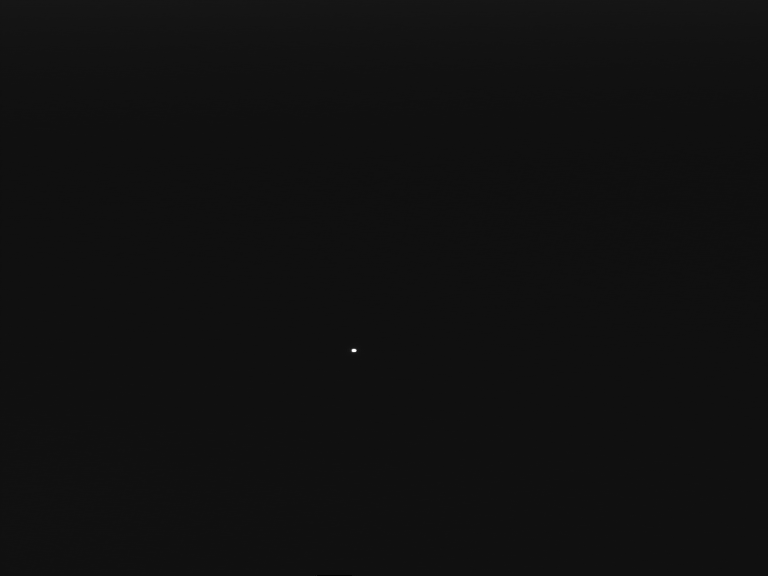

Supplement: S1 Dataset — This archive contains the captured data files used as the basis for the P4P solutions described in the manuscript. The data are provided in a directory hierarchy where each degree of freedom has a separate directory. And the calibration data is the captured data used in the camera calibration. (ZIP) [file pone.0134029.s001.zip › S1_Dataset/Pitch Angle/(49,0,0,0).tif]

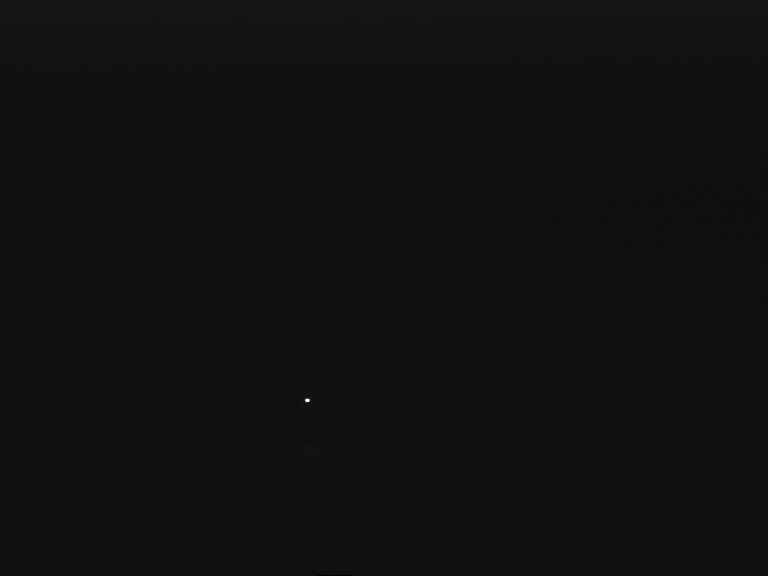

Supplement: S1 Dataset — This archive contains the captured data files used as the basis for the P4P solutions described in the manuscript. The data are provided in a directory hierarchy where each degree of freedom has a separate directory. And the calibration data is the captured data used in the camera calibration. (ZIP) [file pone.0134029.s001.zip › S1_Dataset/Pitch Angle/(49,0,0,1).tif]

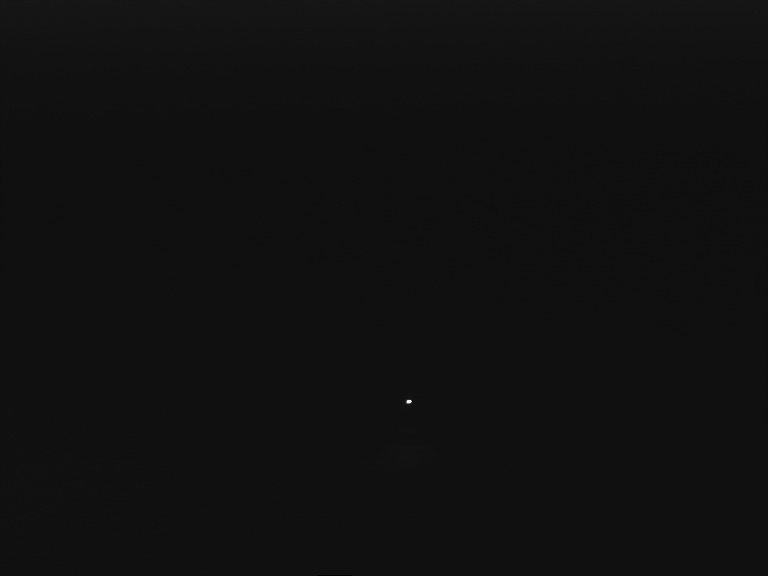

Supplement: S1 Dataset — This archive contains the captured data files used as the basis for the P4P solutions described in the manuscript. The data are provided in a directory hierarchy where each degree of freedom has a separate directory. And the calibration data is the captured data used in the camera calibration. (ZIP) [file pone.0134029.s001.zip › S1_Dataset/Pitch Angle/(49,0,0,2).tif]

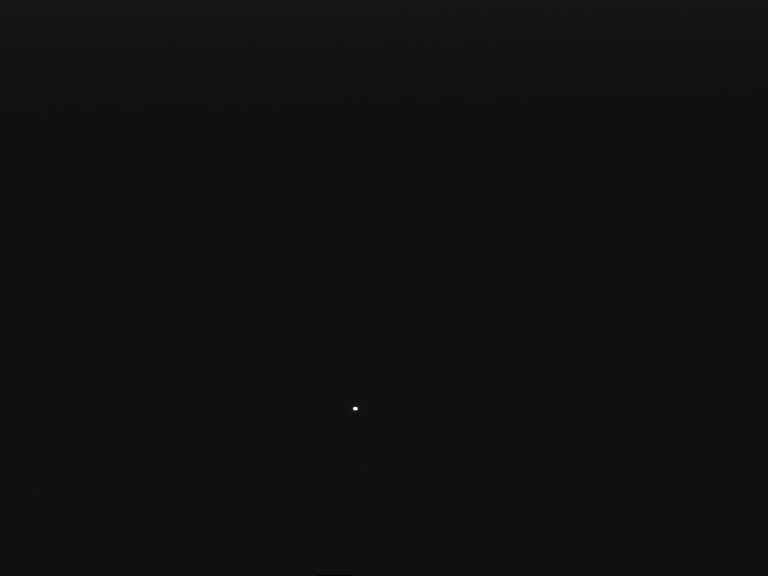

Supplement: S1 Dataset — This archive contains the captured data files used as the basis for the P4P solutions described in the manuscript. The data are provided in a directory hierarchy where each degree of freedom has a separate directory. And the calibration data is the captured data used in the camera calibration. (ZIP) [file pone.0134029.s001.zip › S1_Dataset/Pitch Angle/(49,0,0,3).tif]

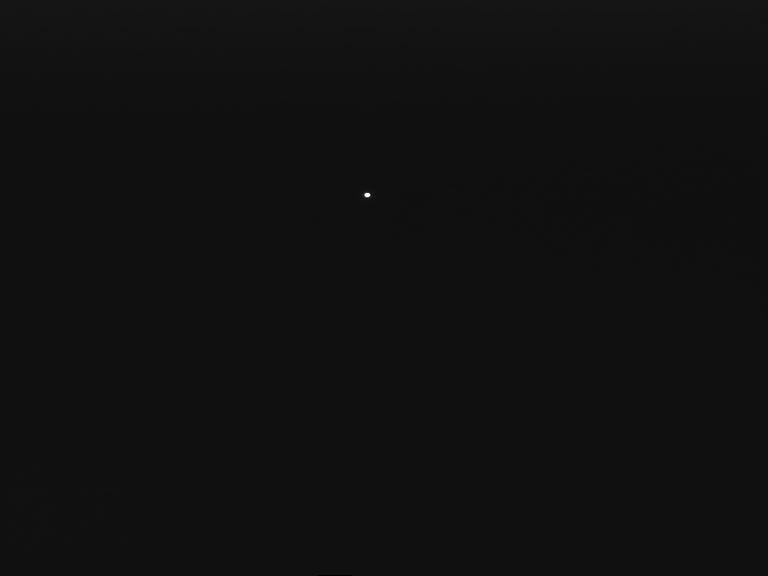

Supplement: S1 Dataset — This archive contains the captured data files used as the basis for the P4P solutions described in the manuscript. The data are provided in a directory hierarchy where each degree of freedom has a separate directory. And the calibration data is the captured data used in the camera calibration. (ZIP) [file pone.0134029.s001.zip › S1_Dataset/Pitch Angle/(5,0,0,0).tif]

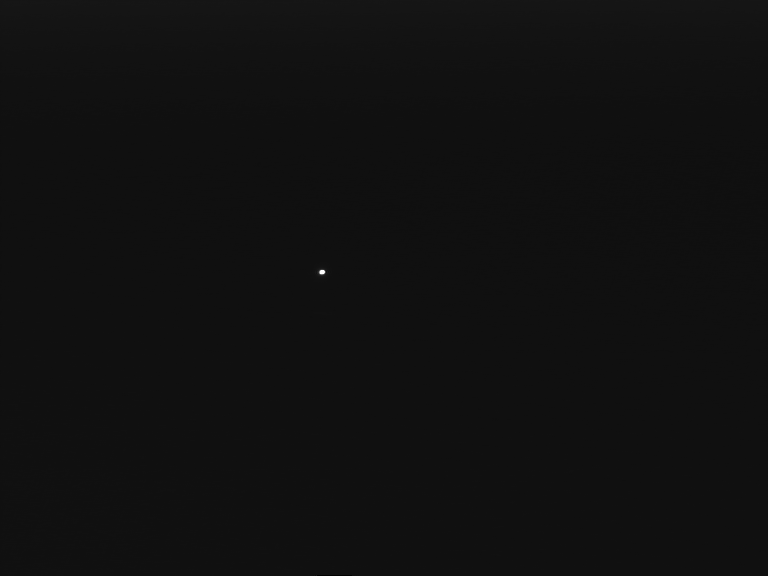

Supplement: S1 Dataset — This archive contains the captured data files used as the basis for the P4P solutions described in the manuscript. The data are provided in a directory hierarchy where each degree of freedom has a separate directory. And the calibration data is the captured data used in the camera calibration. (ZIP) [file pone.0134029.s001.zip › S1_Dataset/Pitch Angle/(5,0,0,1).tif]

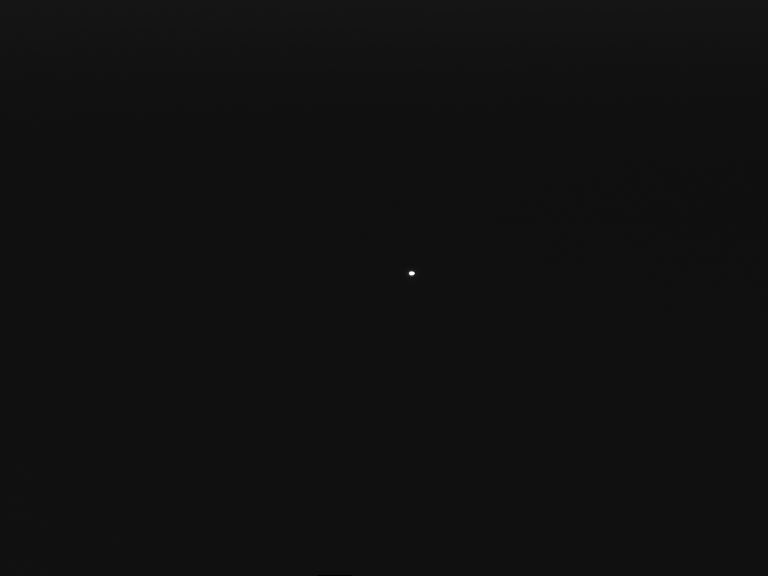

Supplement: S1 Dataset — This archive contains the captured data files used as the basis for the P4P solutions described in the manuscript. The data are provided in a directory hierarchy where each degree of freedom has a separate directory. And the calibration data is the captured data used in the camera calibration. (ZIP) [file pone.0134029.s001.zip › S1_Dataset/Pitch Angle/(5,0,0,2).tif]

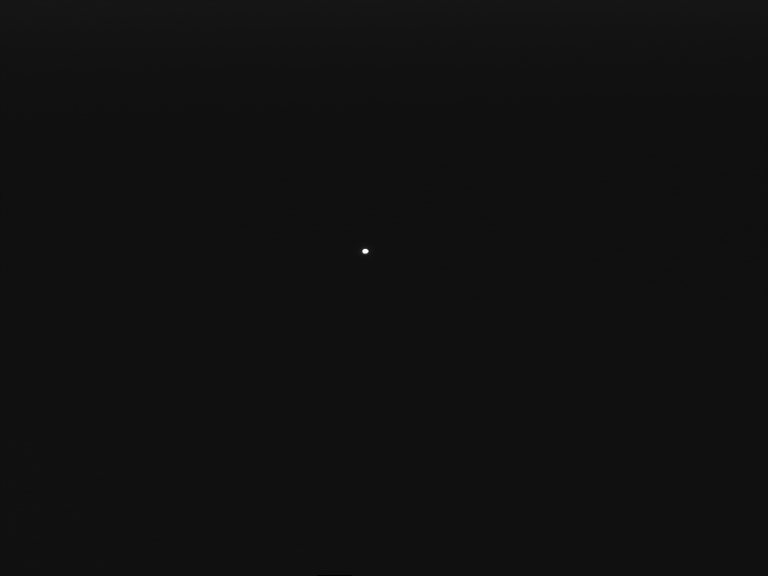

Supplement: S1 Dataset — This archive contains the captured data files used as the basis for the P4P solutions described in the manuscript. The data are provided in a directory hierarchy where each degree of freedom has a separate directory. And the calibration data is the captured data used in the camera calibration. (ZIP) [file pone.0134029.s001.zip › S1_Dataset/Pitch Angle/(5,0,0,3).tif]

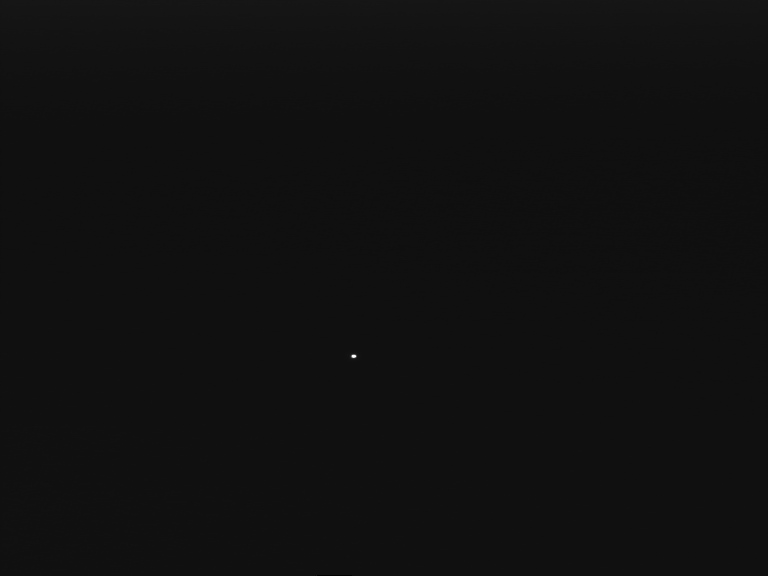

Supplement: S1 Dataset — This archive contains the captured data files used as the basis for the P4P solutions described in the manuscript. The data are provided in a directory hierarchy where each degree of freedom has a separate directory. And the calibration data is the captured data used in the camera calibration. (ZIP) [file pone.0134029.s001.zip › S1_Dataset/Pitch Angle/(50,0,0,0).tif]

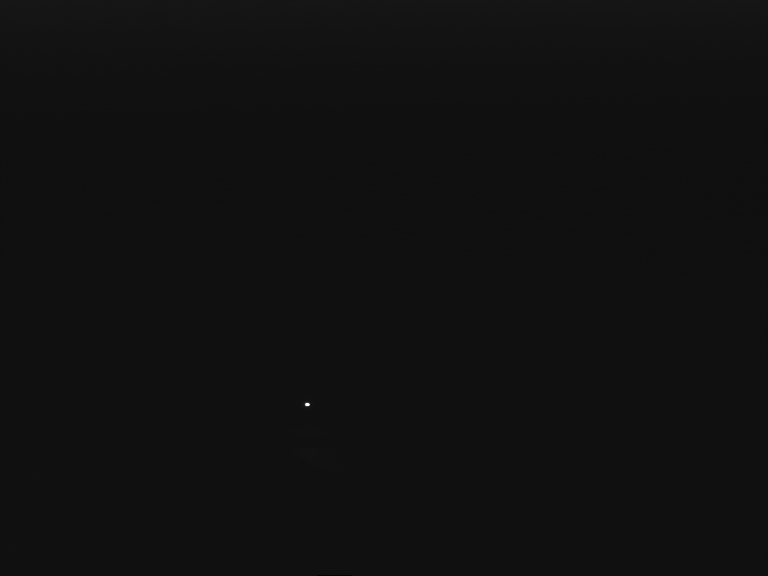

Supplement: S1 Dataset — This archive contains the captured data files used as the basis for the P4P solutions described in the manuscript. The data are provided in a directory hierarchy where each degree of freedom has a separate directory. And the calibration data is the captured data used in the camera calibration. (ZIP) [file pone.0134029.s001.zip › S1_Dataset/Pitch Angle/(50,0,0,1).tif]

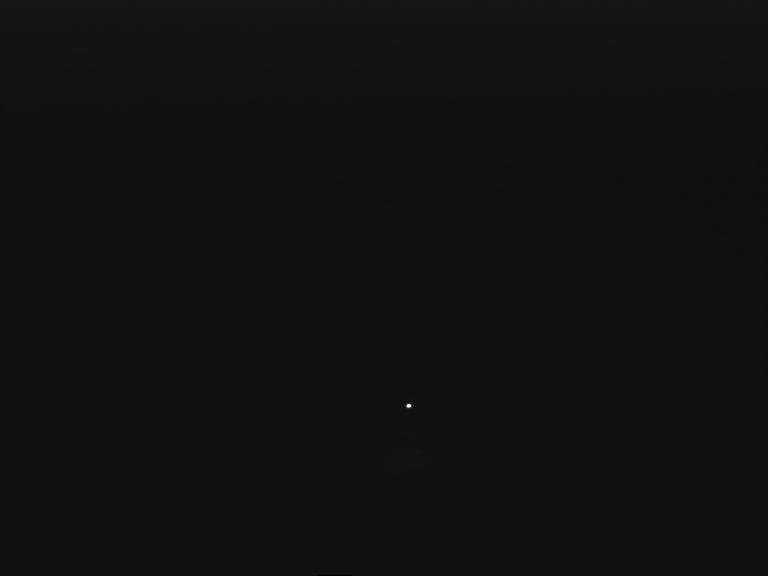

Supplement: S1 Dataset — This archive contains the captured data files used as the basis for the P4P solutions described in the manuscript. The data are provided in a directory hierarchy where each degree of freedom has a separate directory. And the calibration data is the captured data used in the camera calibration. (ZIP) [file pone.0134029.s001.zip › S1_Dataset/Pitch Angle/(50,0,0,2).tif]

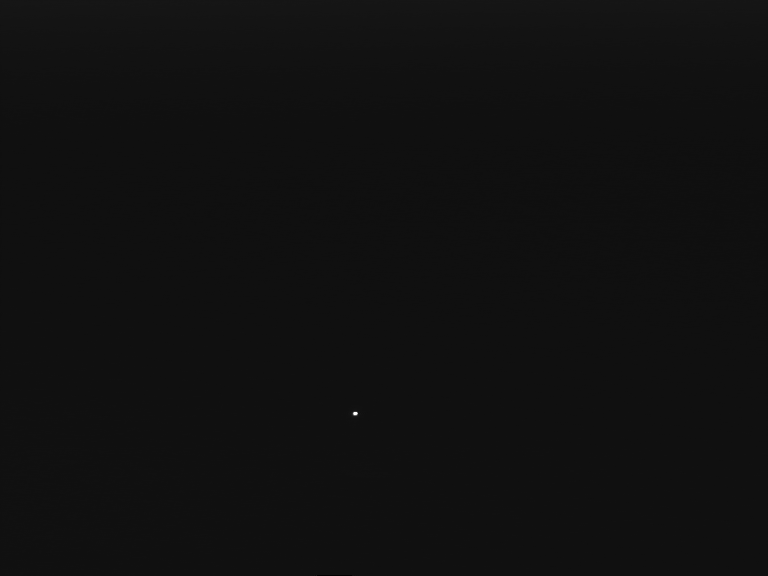

Supplement: S1 Dataset — This archive contains the captured data files used as the basis for the P4P solutions described in the manuscript. The data are provided in a directory hierarchy where each degree of freedom has a separate directory. And the calibration data is the captured data used in the camera calibration. (ZIP) [file pone.0134029.s001.zip › S1_Dataset/Pitch Angle/(50,0,0,3).tif]

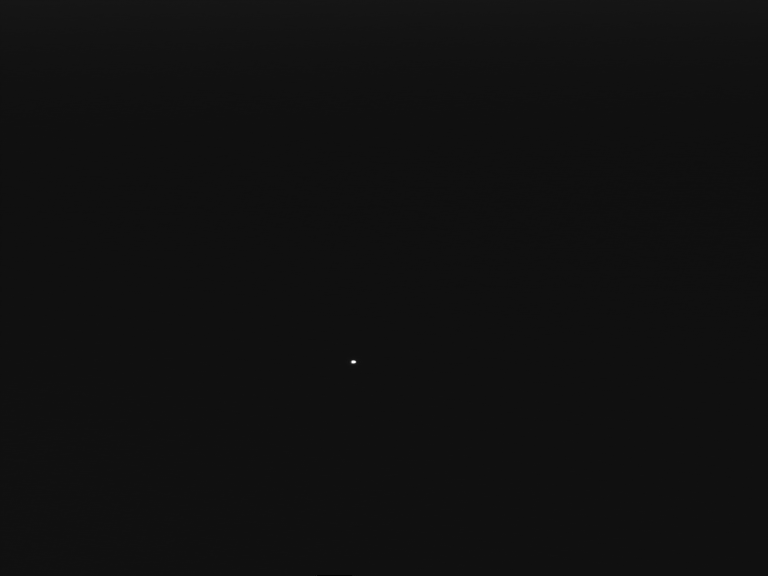

Supplement: S1 Dataset — This archive contains the captured data files used as the basis for the P4P solutions described in the manuscript. The data are provided in a directory hierarchy where each degree of freedom has a separate directory. And the calibration data is the captured data used in the camera calibration. (ZIP) [file pone.0134029.s001.zip › S1_Dataset/Pitch Angle/(51,0,0,0).tif]

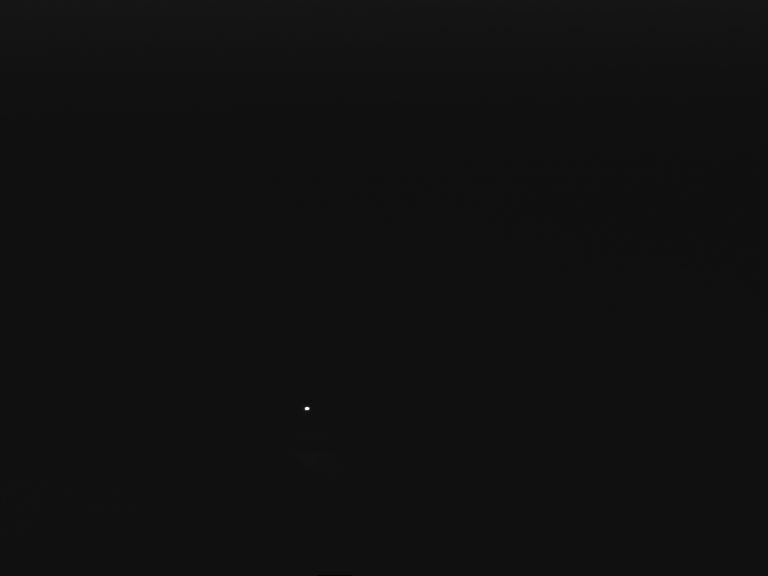

Supplement: S1 Dataset — This archive contains the captured data files used as the basis for the P4P solutions described in the manuscript. The data are provided in a directory hierarchy where each degree of freedom has a separate directory. And the calibration data is the captured data used in the camera calibration. (ZIP) [file pone.0134029.s001.zip › S1_Dataset/Pitch Angle/(51,0,0,1).tif]

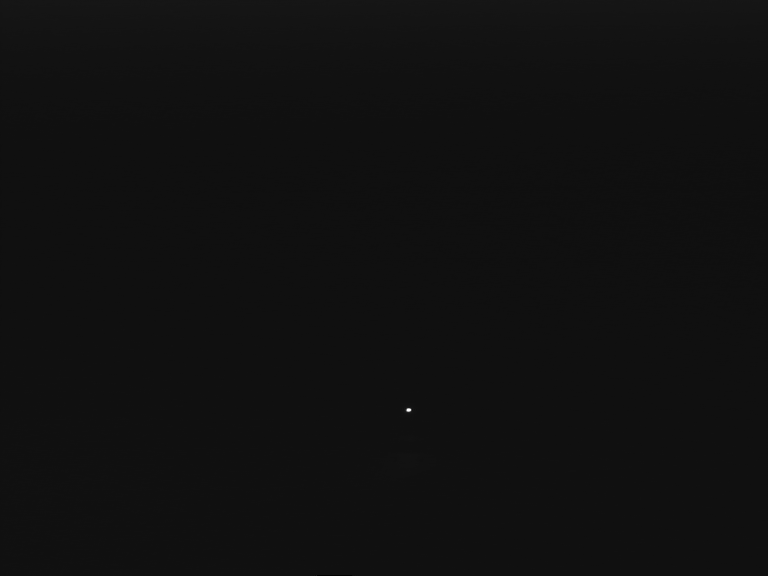

Supplement: S1 Dataset — This archive contains the captured data files used as the basis for the P4P solutions described in the manuscript. The data are provided in a directory hierarchy where each degree of freedom has a separate directory. And the calibration data is the captured data used in the camera calibration. (ZIP) [file pone.0134029.s001.zip › S1_Dataset/Pitch Angle/(51,0,0,2).tif]

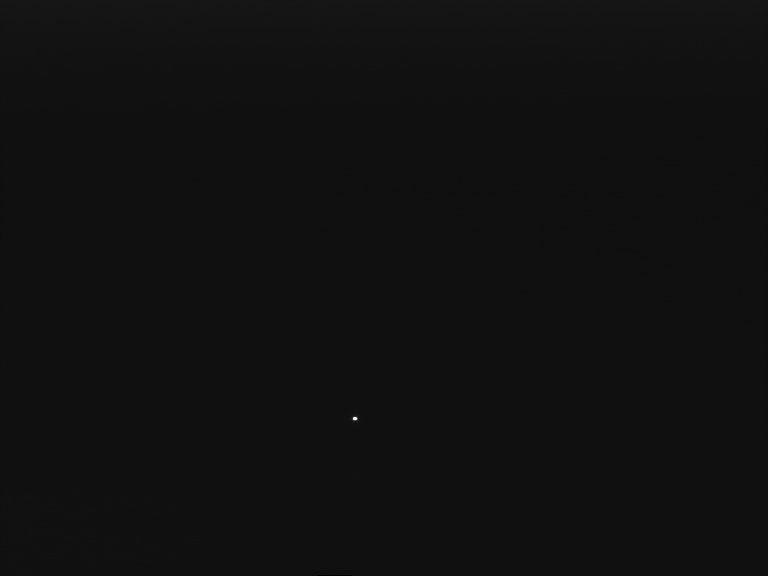

Supplement: S1 Dataset — This archive contains the captured data files used as the basis for the P4P solutions described in the manuscript. The data are provided in a directory hierarchy where each degree of freedom has a separate directory. And the calibration data is the captured data used in the camera calibration. (ZIP) [file pone.0134029.s001.zip › S1_Dataset/Pitch Angle/(51,0,0,3).tif]

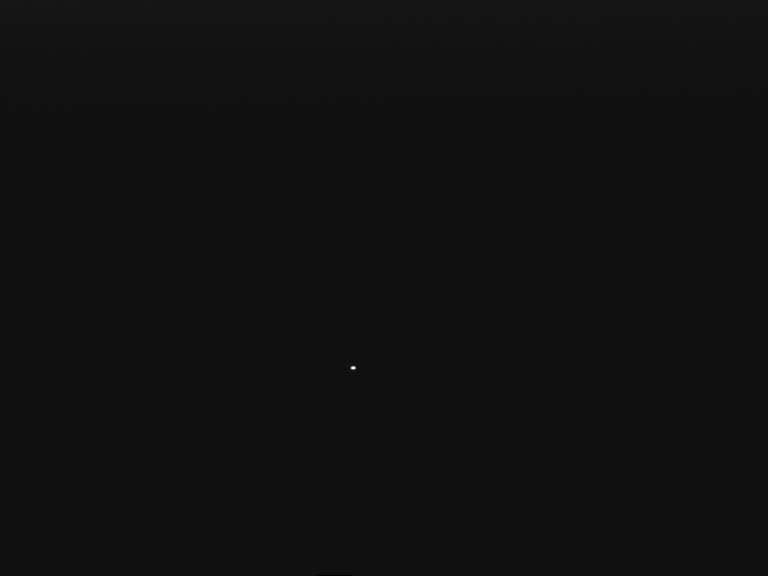

Supplement: S1 Dataset — This archive contains the captured data files used as the basis for the P4P solutions described in the manuscript. The data are provided in a directory hierarchy where each degree of freedom has a separate directory. And the calibration data is the captured data used in the camera calibration. (ZIP) [file pone.0134029.s001.zip › S1_Dataset/Pitch Angle/(52,0,0,0).tif]

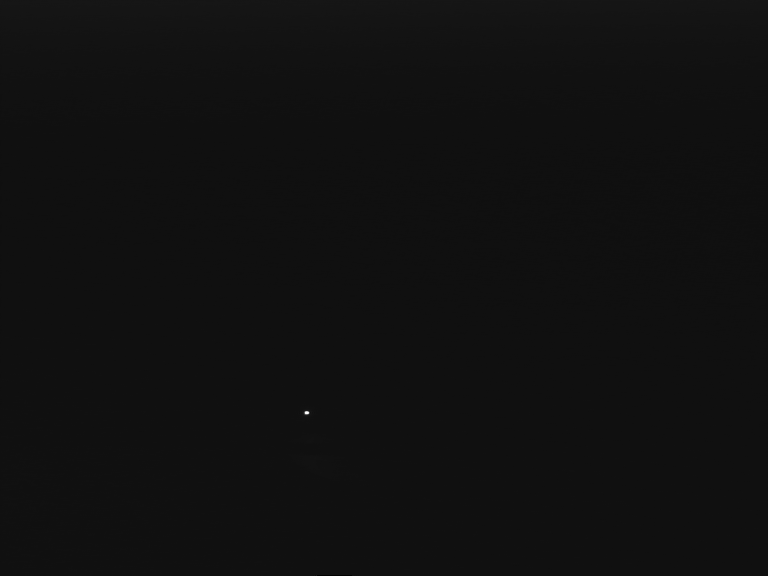

Supplement: S1 Dataset — This archive contains the captured data files used as the basis for the P4P solutions described in the manuscript. The data are provided in a directory hierarchy where each degree of freedom has a separate directory. And the calibration data is the captured data used in the camera calibration. (ZIP) [file pone.0134029.s001.zip › S1_Dataset/Pitch Angle/(52,0,0,1).tif]
