# Supplementary figures and images for: A New Pose Estimation Algorithm Using a Perspective-Ray-Based Scaled Orthographic Projection with Iteration (part 1 of 2)
Source: PLoS One. 2015 Jul 21;10(7):e0134029. doi: 10.1371/journal.pone.0134029 (PMC4509906; doi:10.1371/journal.pone.0134029)

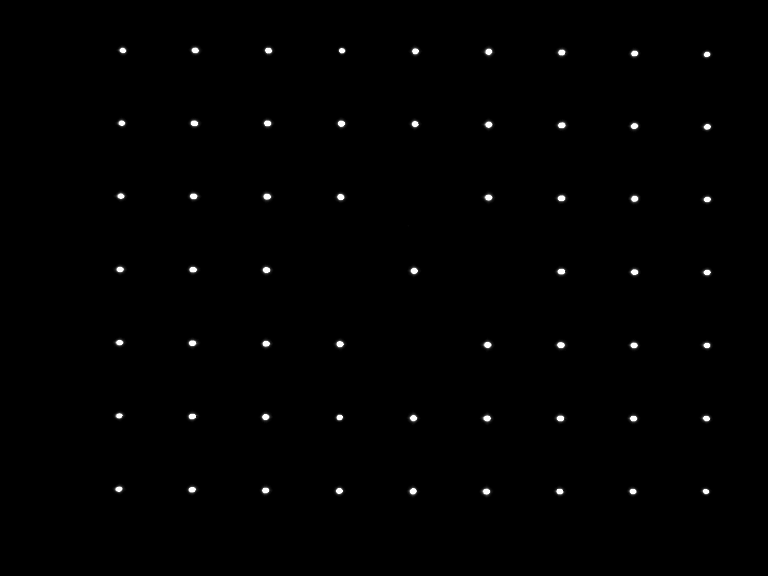

Supplement: S1 Dataset — This archive contains the captured data files used as the basis for the P4P solutions described in the manuscript. The data are provided in a directory hierarchy where each degree of freedom has a separate directory. And the calibration data is the captured data used in the camera calibration. (ZIP) [file pone.0134029.s001.zip › S1_Dataset/Calibration Data/0.tif]

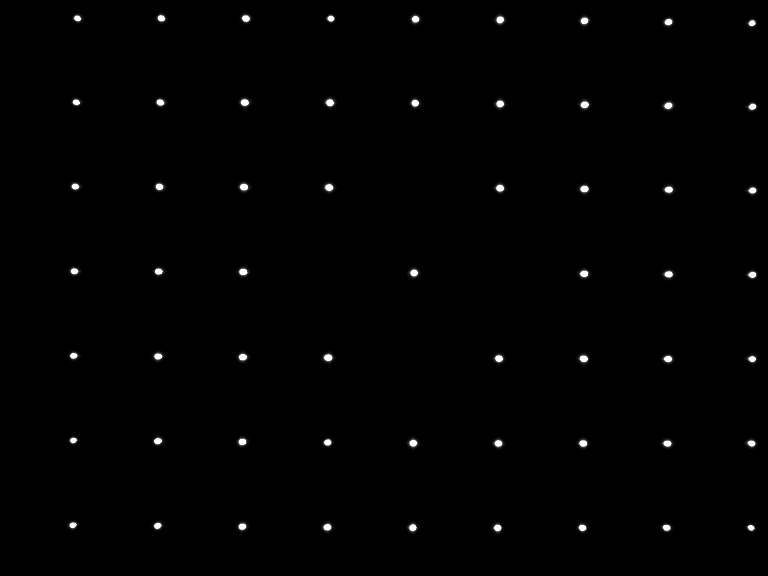

Supplement: S1 Dataset — This archive contains the captured data files used as the basis for the P4P solutions described in the manuscript. The data are provided in a directory hierarchy where each degree of freedom has a separate directory. And the calibration data is the captured data used in the camera calibration. (ZIP) [file pone.0134029.s001.zip › S1_Dataset/Calibration Data/120.tif]

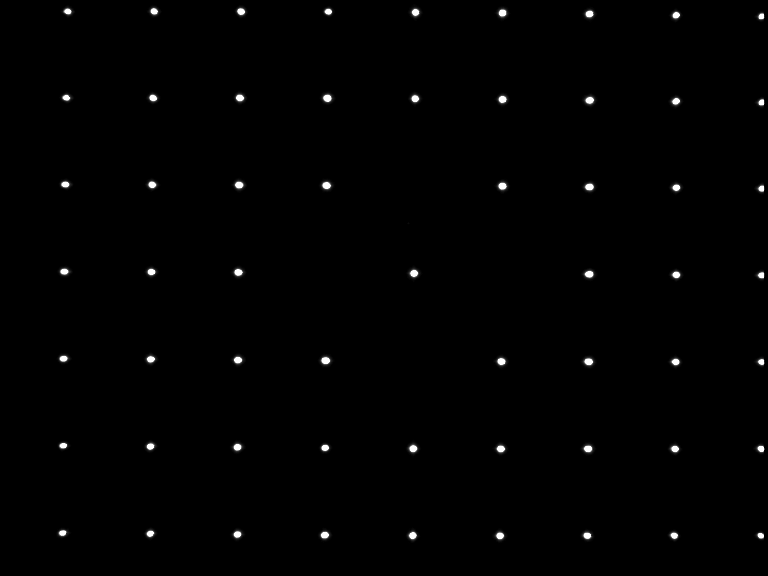

Supplement: S1 Dataset — This archive contains the captured data files used as the basis for the P4P solutions described in the manuscript. The data are provided in a directory hierarchy where each degree of freedom has a separate directory. And the calibration data is the captured data used in the camera calibration. (ZIP) [file pone.0134029.s001.zip › S1_Dataset/Calibration Data/150.tif]

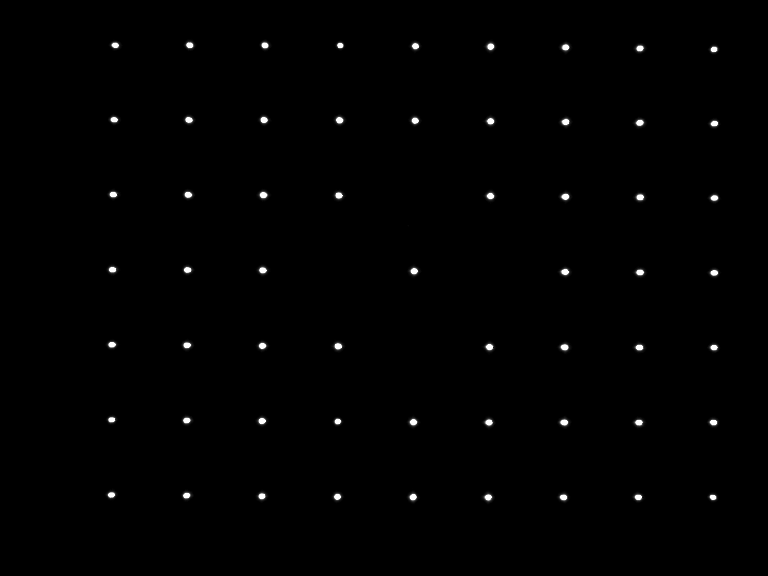

Supplement: S1 Dataset — This archive contains the captured data files used as the basis for the P4P solutions described in the manuscript. The data are provided in a directory hierarchy where each degree of freedom has a separate directory. And the calibration data is the captured data used in the camera calibration. (ZIP) [file pone.0134029.s001.zip › S1_Dataset/Calibration Data/30.tif]

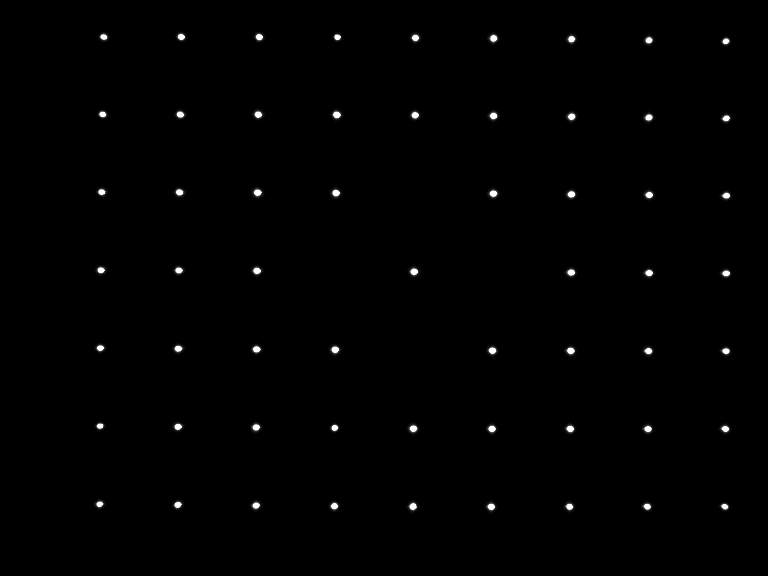

Supplement: S1 Dataset — This archive contains the captured data files used as the basis for the P4P solutions described in the manuscript. The data are provided in a directory hierarchy where each degree of freedom has a separate directory. And the calibration data is the captured data used in the camera calibration. (ZIP) [file pone.0134029.s001.zip › S1_Dataset/Calibration Data/60.tif]

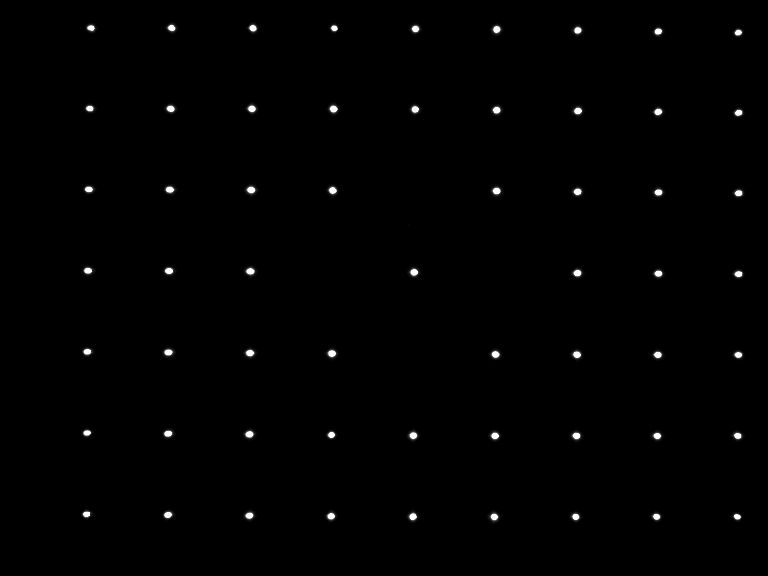

Supplement: S1 Dataset — This archive contains the captured data files used as the basis for the P4P solutions described in the manuscript. The data are provided in a directory hierarchy where each degree of freedom has a separate directory. And the calibration data is the captured data used in the camera calibration. (ZIP) [file pone.0134029.s001.zip › S1_Dataset/Calibration Data/90.tif]

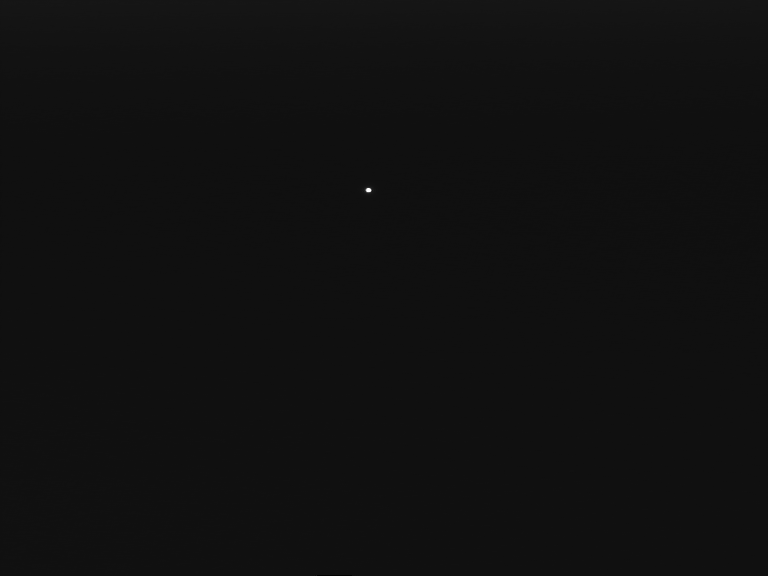

Supplement: S1 Dataset — This archive contains the captured data files used as the basis for the P4P solutions described in the manuscript. The data are provided in a directory hierarchy where each degree of freedom has a separate directory. And the calibration data is the captured data used in the camera calibration. (ZIP) [file pone.0134029.s001.zip › S1_Dataset/Pitch Angle/(0,0,0,0).tif]

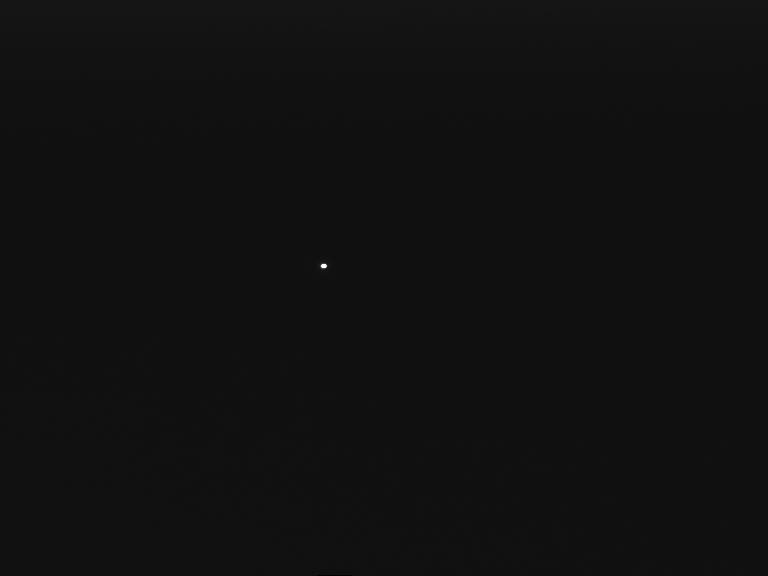

Supplement: S1 Dataset — This archive contains the captured data files used as the basis for the P4P solutions described in the manuscript. The data are provided in a directory hierarchy where each degree of freedom has a separate directory. And the calibration data is the captured data used in the camera calibration. (ZIP) [file pone.0134029.s001.zip › S1_Dataset/Pitch Angle/(0,0,0,1).tif]

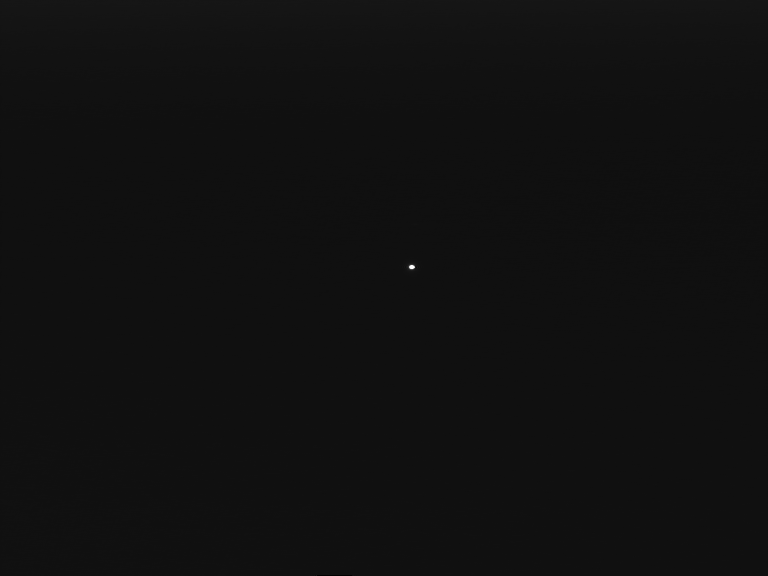

Supplement: S1 Dataset — This archive contains the captured data files used as the basis for the P4P solutions described in the manuscript. The data are provided in a directory hierarchy where each degree of freedom has a separate directory. And the calibration data is the captured data used in the camera calibration. (ZIP) [file pone.0134029.s001.zip › S1_Dataset/Pitch Angle/(0,0,0,2).tif]

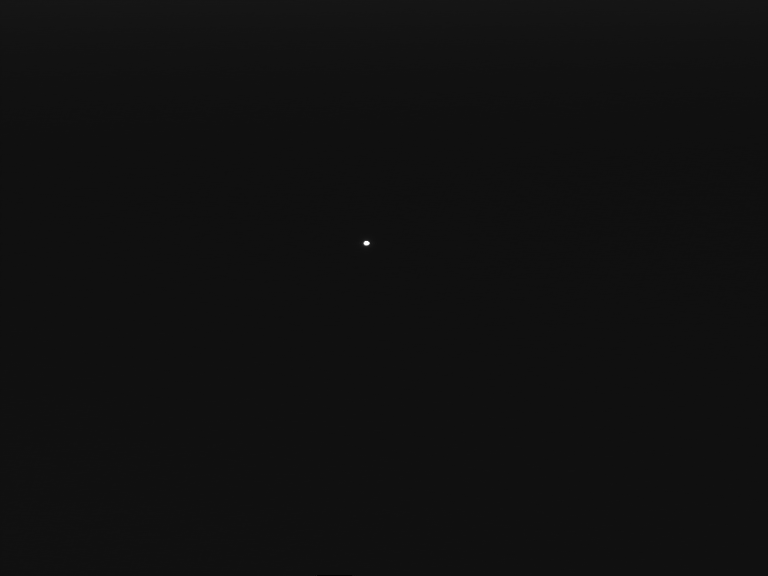

Supplement: S1 Dataset — This archive contains the captured data files used as the basis for the P4P solutions described in the manuscript. The data are provided in a directory hierarchy where each degree of freedom has a separate directory. And the calibration data is the captured data used in the camera calibration. (ZIP) [file pone.0134029.s001.zip › S1_Dataset/Pitch Angle/(0,0,0,3).tif]

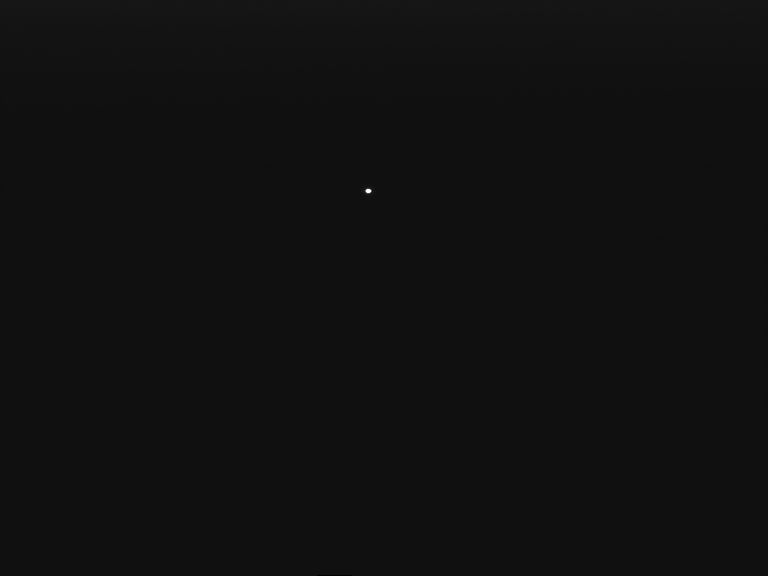

Supplement: S1 Dataset — This archive contains the captured data files used as the basis for the P4P solutions described in the manuscript. The data are provided in a directory hierarchy where each degree of freedom has a separate directory. And the calibration data is the captured data used in the camera calibration. (ZIP) [file pone.0134029.s001.zip › S1_Dataset/Pitch Angle/(1,0,0,0).tif]

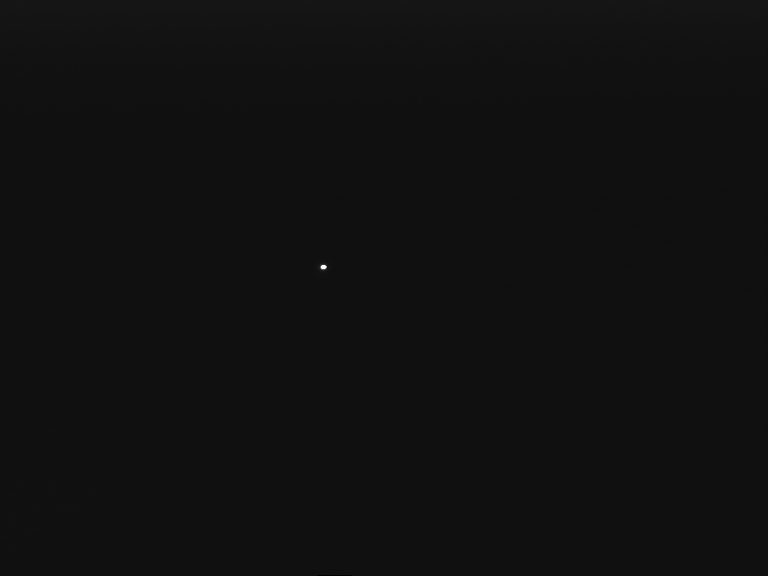

Supplement: S1 Dataset — This archive contains the captured data files used as the basis for the P4P solutions described in the manuscript. The data are provided in a directory hierarchy where each degree of freedom has a separate directory. And the calibration data is the captured data used in the camera calibration. (ZIP) [file pone.0134029.s001.zip › S1_Dataset/Pitch Angle/(1,0,0,1).tif]

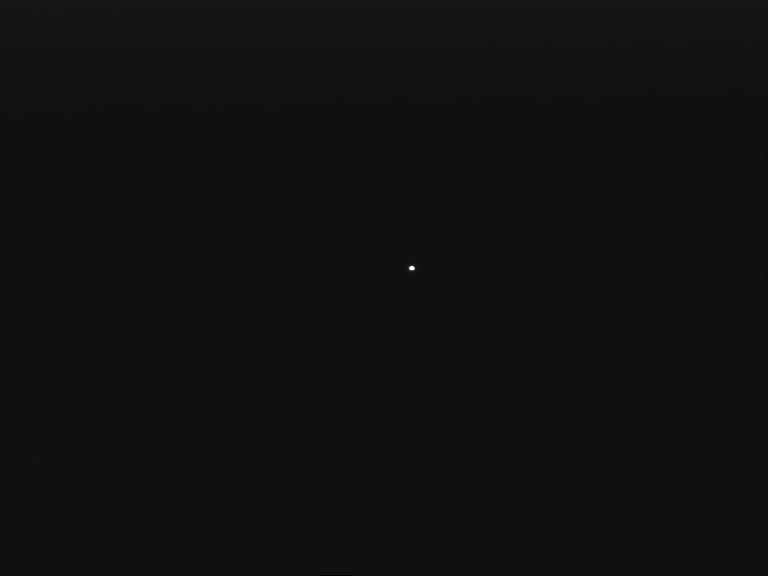

Supplement: S1 Dataset — This archive contains the captured data files used as the basis for the P4P solutions described in the manuscript. The data are provided in a directory hierarchy where each degree of freedom has a separate directory. And the calibration data is the captured data used in the camera calibration. (ZIP) [file pone.0134029.s001.zip › S1_Dataset/Pitch Angle/(1,0,0,2).tif]

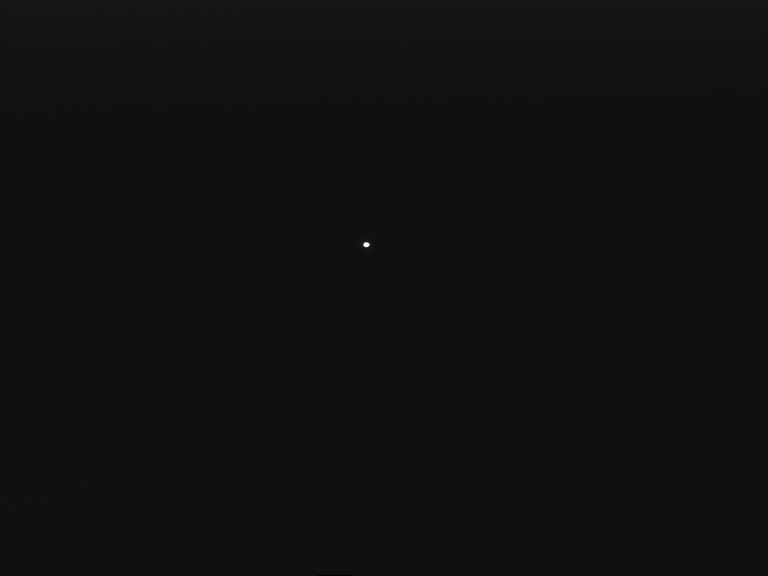

Supplement: S1 Dataset — This archive contains the captured data files used as the basis for the P4P solutions described in the manuscript. The data are provided in a directory hierarchy where each degree of freedom has a separate directory. And the calibration data is the captured data used in the camera calibration. (ZIP) [file pone.0134029.s001.zip › S1_Dataset/Pitch Angle/(1,0,0,3).tif]

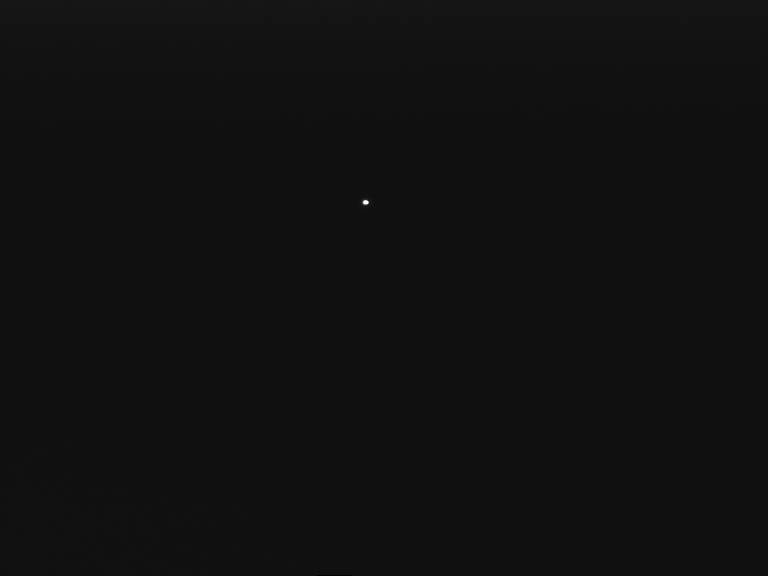

Supplement: S1 Dataset — This archive contains the captured data files used as the basis for the P4P solutions described in the manuscript. The data are provided in a directory hierarchy where each degree of freedom has a separate directory. And the calibration data is the captured data used in the camera calibration. (ZIP) [file pone.0134029.s001.zip › S1_Dataset/Pitch Angle/(10,0,0,0).tif]

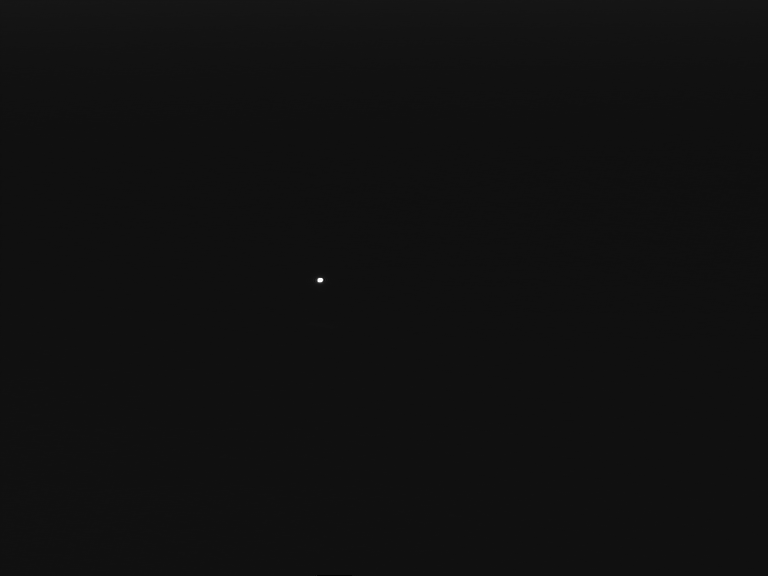

Supplement: S1 Dataset — This archive contains the captured data files used as the basis for the P4P solutions described in the manuscript. The data are provided in a directory hierarchy where each degree of freedom has a separate directory. And the calibration data is the captured data used in the camera calibration. (ZIP) [file pone.0134029.s001.zip › S1_Dataset/Pitch Angle/(10,0,0,1).tif]

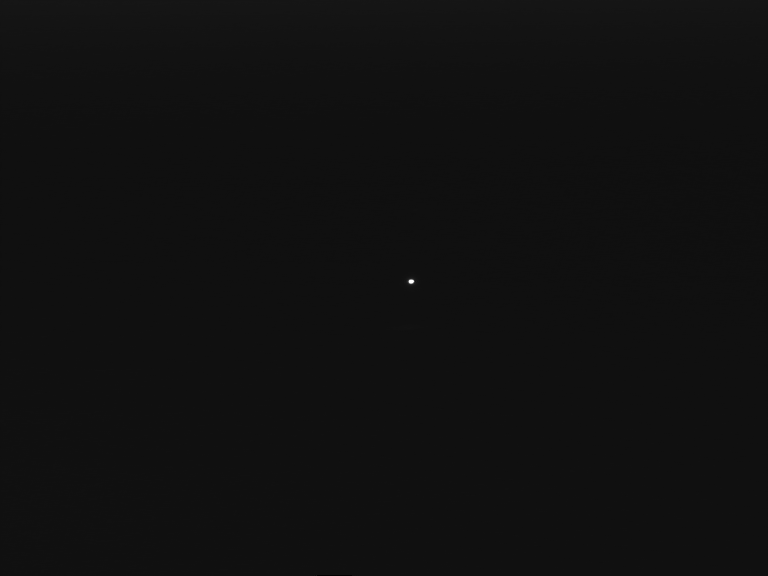

Supplement: S1 Dataset — This archive contains the captured data files used as the basis for the P4P solutions described in the manuscript. The data are provided in a directory hierarchy where each degree of freedom has a separate directory. And the calibration data is the captured data used in the camera calibration. (ZIP) [file pone.0134029.s001.zip › S1_Dataset/Pitch Angle/(10,0,0,2).tif]

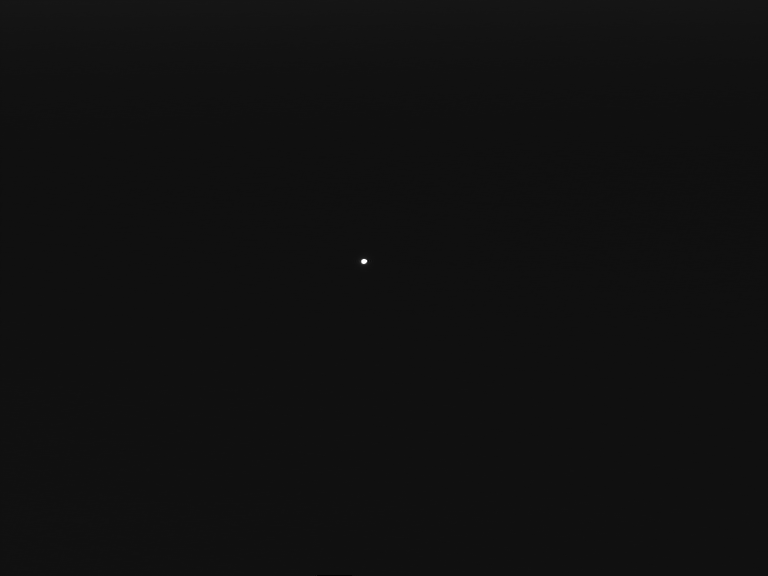

Supplement: S1 Dataset — This archive contains the captured data files used as the basis for the P4P solutions described in the manuscript. The data are provided in a directory hierarchy where each degree of freedom has a separate directory. And the calibration data is the captured data used in the camera calibration. (ZIP) [file pone.0134029.s001.zip › S1_Dataset/Pitch Angle/(10,0,0,3).tif]

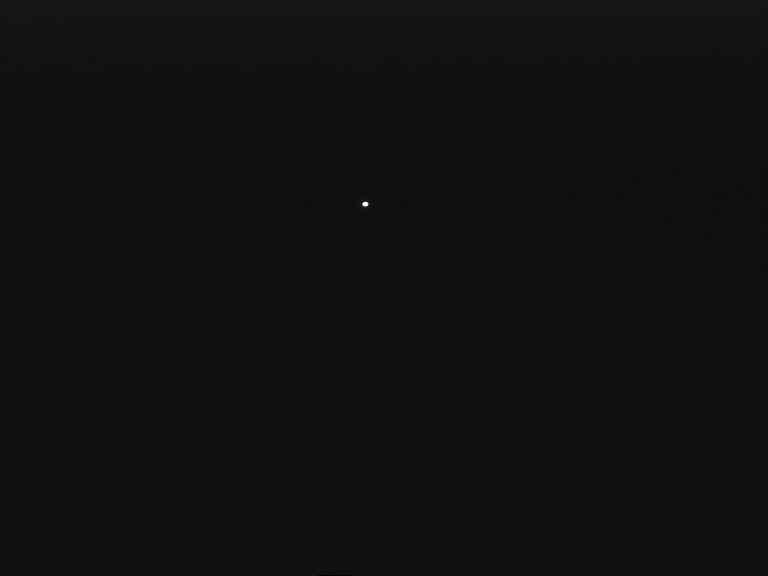

Supplement: S1 Dataset — This archive contains the captured data files used as the basis for the P4P solutions described in the manuscript. The data are provided in a directory hierarchy where each degree of freedom has a separate directory. And the calibration data is the captured data used in the camera calibration. (ZIP) [file pone.0134029.s001.zip › S1_Dataset/Pitch Angle/(11,0,0,0).tif]

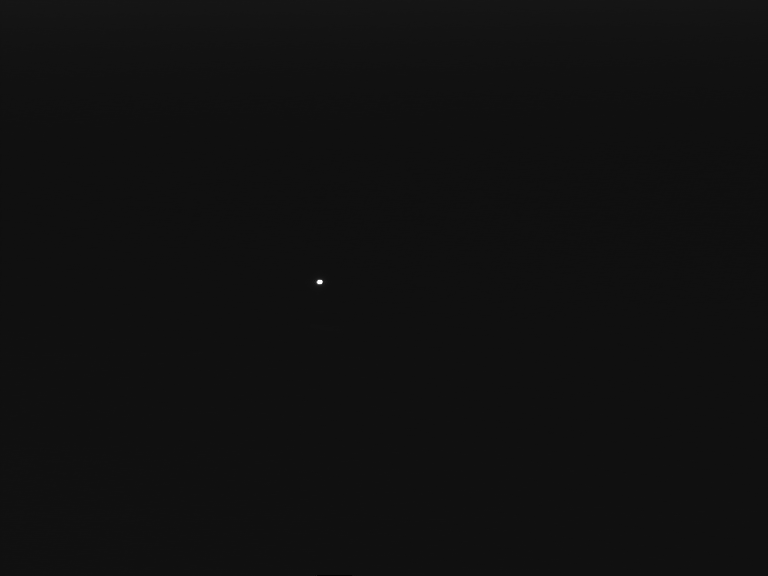

Supplement: S1 Dataset — This archive contains the captured data files used as the basis for the P4P solutions described in the manuscript. The data are provided in a directory hierarchy where each degree of freedom has a separate directory. And the calibration data is the captured data used in the camera calibration. (ZIP) [file pone.0134029.s001.zip › S1_Dataset/Pitch Angle/(11,0,0,1).tif]

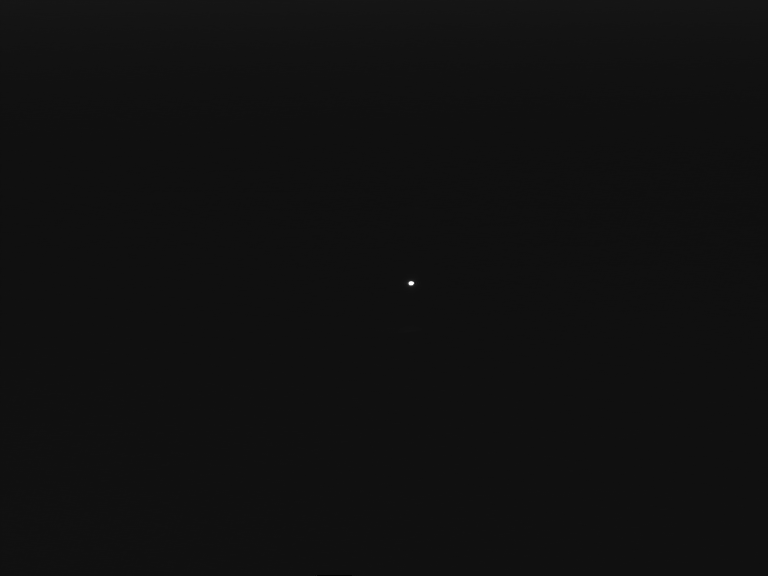

Supplement: S1 Dataset — This archive contains the captured data files used as the basis for the P4P solutions described in the manuscript. The data are provided in a directory hierarchy where each degree of freedom has a separate directory. And the calibration data is the captured data used in the camera calibration. (ZIP) [file pone.0134029.s001.zip › S1_Dataset/Pitch Angle/(11,0,0,2).tif]

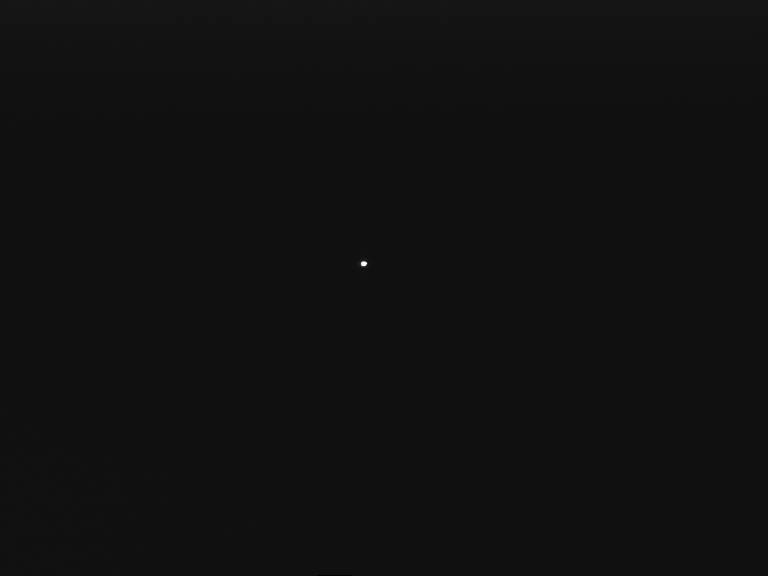

Supplement: S1 Dataset — This archive contains the captured data files used as the basis for the P4P solutions described in the manuscript. The data are provided in a directory hierarchy where each degree of freedom has a separate directory. And the calibration data is the captured data used in the camera calibration. (ZIP) [file pone.0134029.s001.zip › S1_Dataset/Pitch Angle/(11,0,0,3).tif]

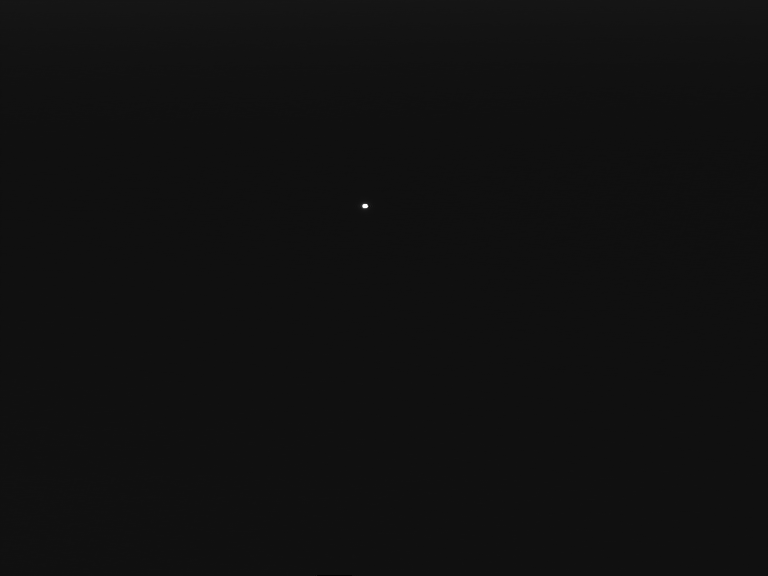

Supplement: S1 Dataset — This archive contains the captured data files used as the basis for the P4P solutions described in the manuscript. The data are provided in a directory hierarchy where each degree of freedom has a separate directory. And the calibration data is the captured data used in the camera calibration. (ZIP) [file pone.0134029.s001.zip › S1_Dataset/Pitch Angle/(12,0,0,0).tif]

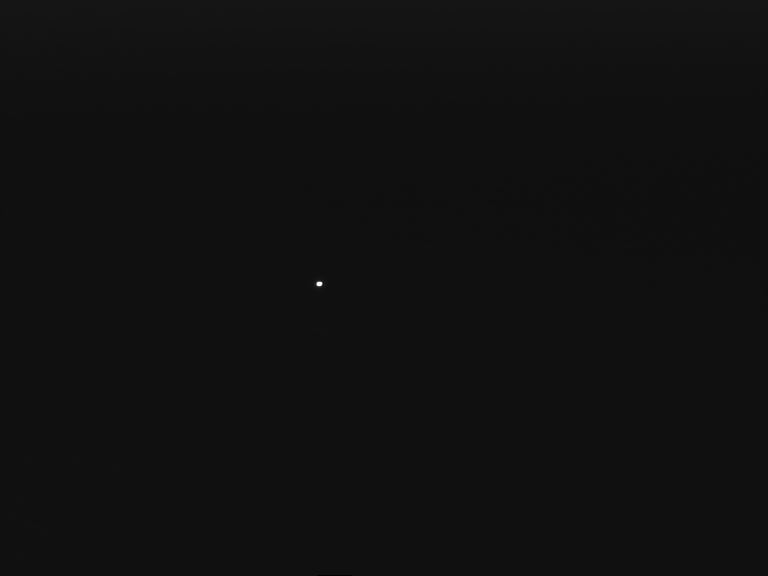

Supplement: S1 Dataset — This archive contains the captured data files used as the basis for the P4P solutions described in the manuscript. The data are provided in a directory hierarchy where each degree of freedom has a separate directory. And the calibration data is the captured data used in the camera calibration. (ZIP) [file pone.0134029.s001.zip › S1_Dataset/Pitch Angle/(12,0,0,1).tif]

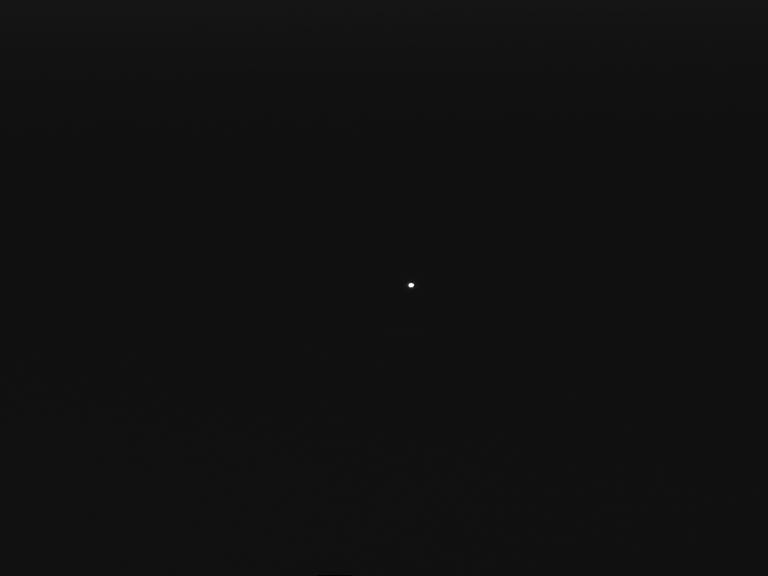

Supplement: S1 Dataset — This archive contains the captured data files used as the basis for the P4P solutions described in the manuscript. The data are provided in a directory hierarchy where each degree of freedom has a separate directory. And the calibration data is the captured data used in the camera calibration. (ZIP) [file pone.0134029.s001.zip › S1_Dataset/Pitch Angle/(12,0,0,2).tif]

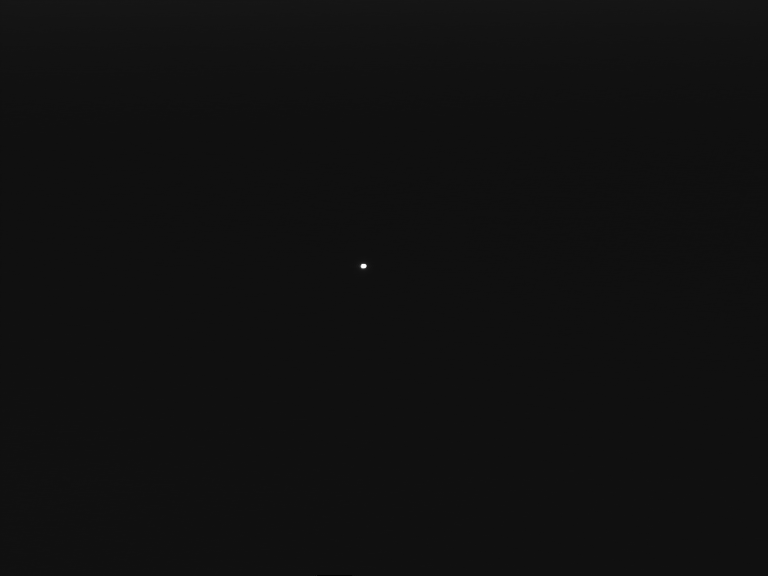

Supplement: S1 Dataset — This archive contains the captured data files used as the basis for the P4P solutions described in the manuscript. The data are provided in a directory hierarchy where each degree of freedom has a separate directory. And the calibration data is the captured data used in the camera calibration. (ZIP) [file pone.0134029.s001.zip › S1_Dataset/Pitch Angle/(12,0,0,3).tif]

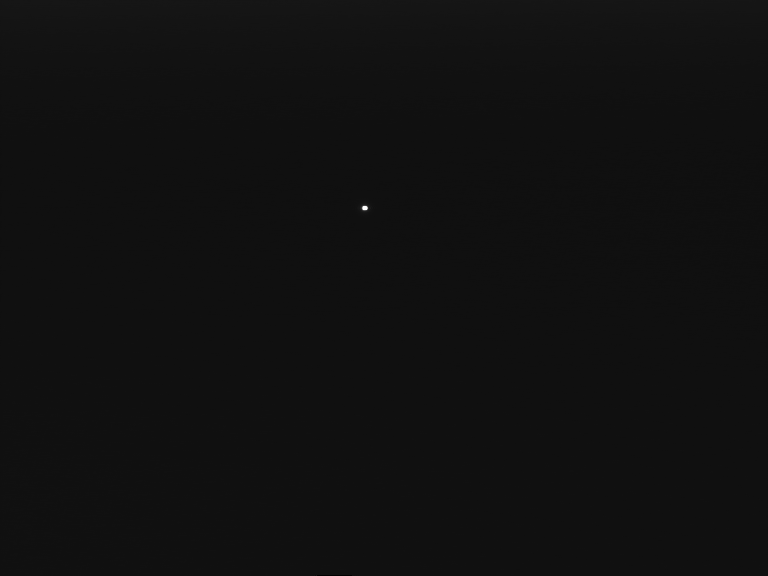

Supplement: S1 Dataset — This archive contains the captured data files used as the basis for the P4P solutions described in the manuscript. The data are provided in a directory hierarchy where each degree of freedom has a separate directory. And the calibration data is the captured data used in the camera calibration. (ZIP) [file pone.0134029.s001.zip › S1_Dataset/Pitch Angle/(13,0,0,0).tif]

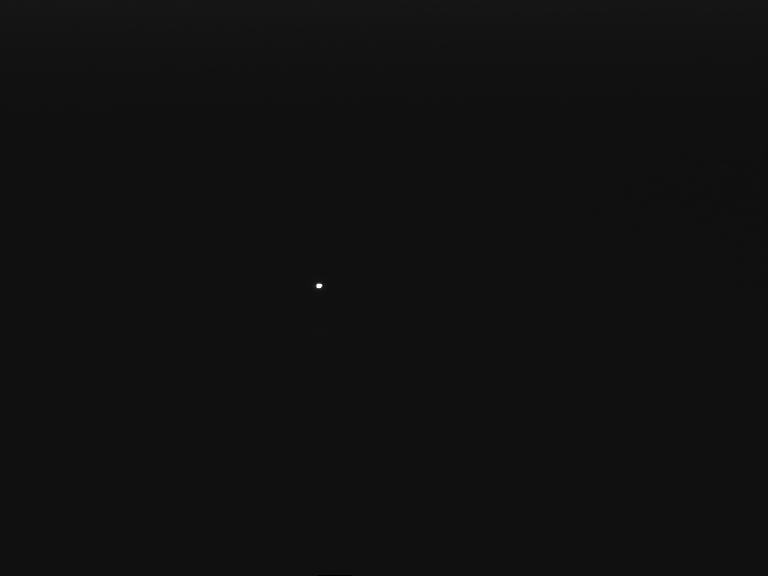

Supplement: S1 Dataset — This archive contains the captured data files used as the basis for the P4P solutions described in the manuscript. The data are provided in a directory hierarchy where each degree of freedom has a separate directory. And the calibration data is the captured data used in the camera calibration. (ZIP) [file pone.0134029.s001.zip › S1_Dataset/Pitch Angle/(13,0,0,1).tif]

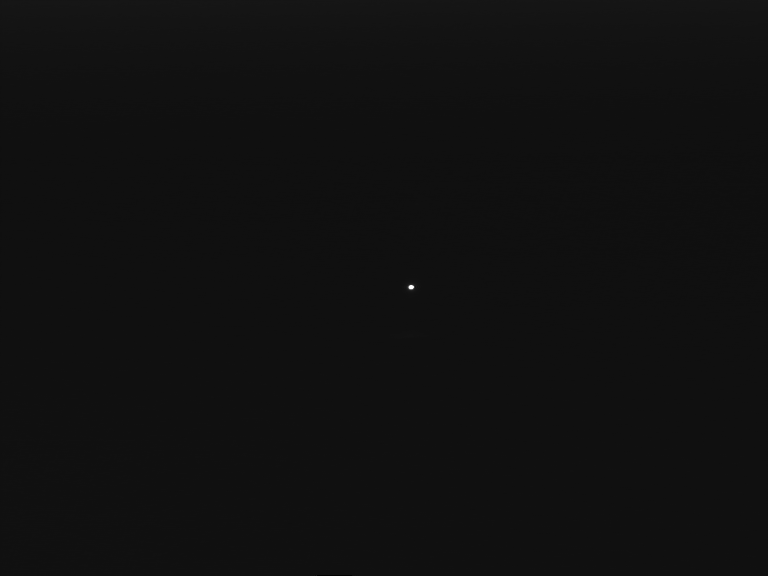

Supplement: S1 Dataset — This archive contains the captured data files used as the basis for the P4P solutions described in the manuscript. The data are provided in a directory hierarchy where each degree of freedom has a separate directory. And the calibration data is the captured data used in the camera calibration. (ZIP) [file pone.0134029.s001.zip › S1_Dataset/Pitch Angle/(13,0,0,2).tif]

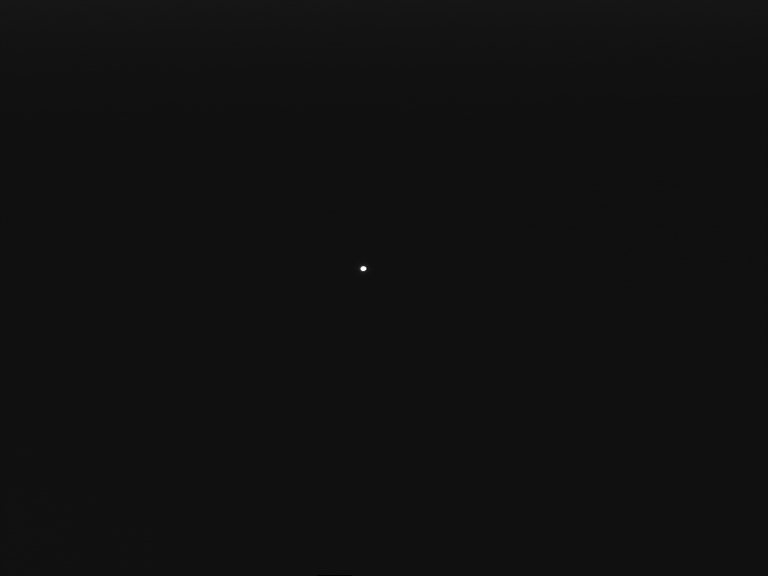

Supplement: S1 Dataset — This archive contains the captured data files used as the basis for the P4P solutions described in the manuscript. The data are provided in a directory hierarchy where each degree of freedom has a separate directory. And the calibration data is the captured data used in the camera calibration. (ZIP) [file pone.0134029.s001.zip › S1_Dataset/Pitch Angle/(13,0,0,3).tif]

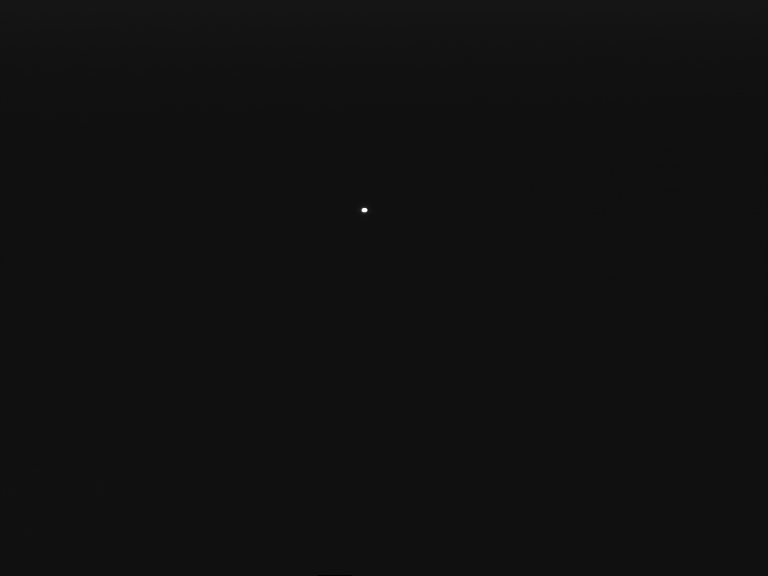

Supplement: S1 Dataset — This archive contains the captured data files used as the basis for the P4P solutions described in the manuscript. The data are provided in a directory hierarchy where each degree of freedom has a separate directory. And the calibration data is the captured data used in the camera calibration. (ZIP) [file pone.0134029.s001.zip › S1_Dataset/Pitch Angle/(14,0,0,0).tif]

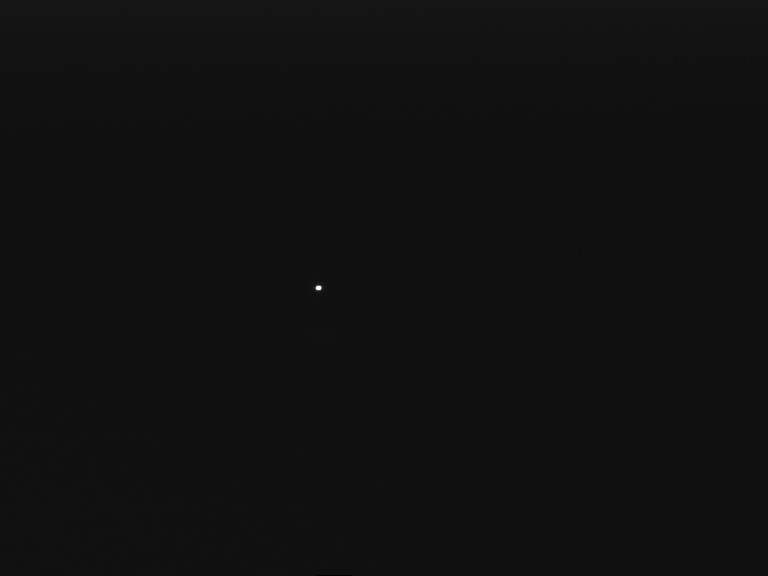

Supplement: S1 Dataset — This archive contains the captured data files used as the basis for the P4P solutions described in the manuscript. The data are provided in a directory hierarchy where each degree of freedom has a separate directory. And the calibration data is the captured data used in the camera calibration. (ZIP) [file pone.0134029.s001.zip › S1_Dataset/Pitch Angle/(14,0,0,1).tif]

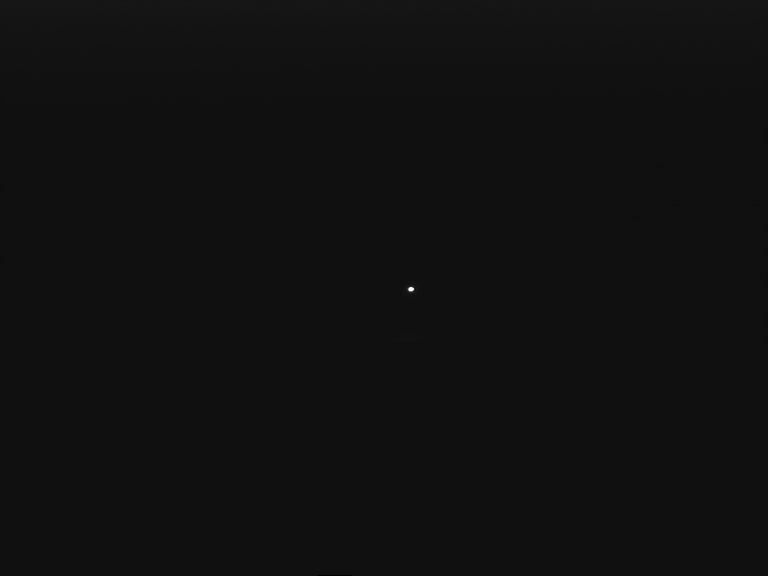

Supplement: S1 Dataset — This archive contains the captured data files used as the basis for the P4P solutions described in the manuscript. The data are provided in a directory hierarchy where each degree of freedom has a separate directory. And the calibration data is the captured data used in the camera calibration. (ZIP) [file pone.0134029.s001.zip › S1_Dataset/Pitch Angle/(14,0,0,2).tif]

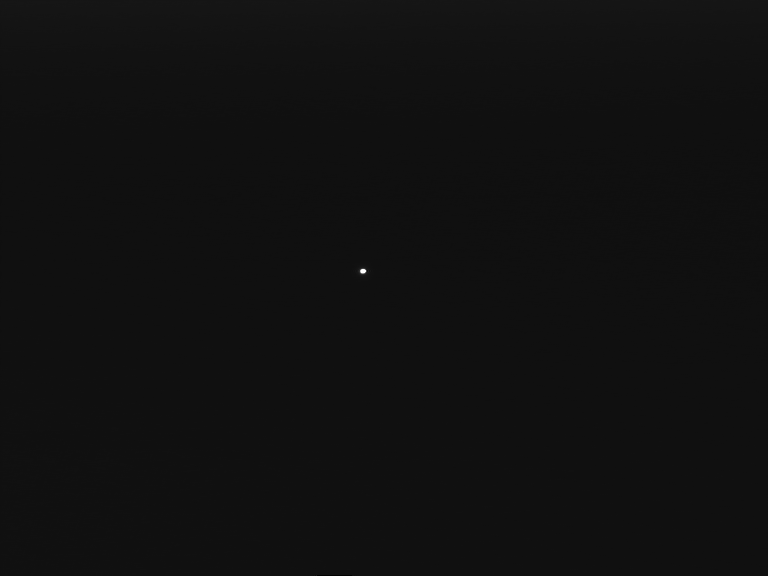

Supplement: S1 Dataset — This archive contains the captured data files used as the basis for the P4P solutions described in the manuscript. The data are provided in a directory hierarchy where each degree of freedom has a separate directory. And the calibration data is the captured data used in the camera calibration. (ZIP) [file pone.0134029.s001.zip › S1_Dataset/Pitch Angle/(14,0,0,3).tif]

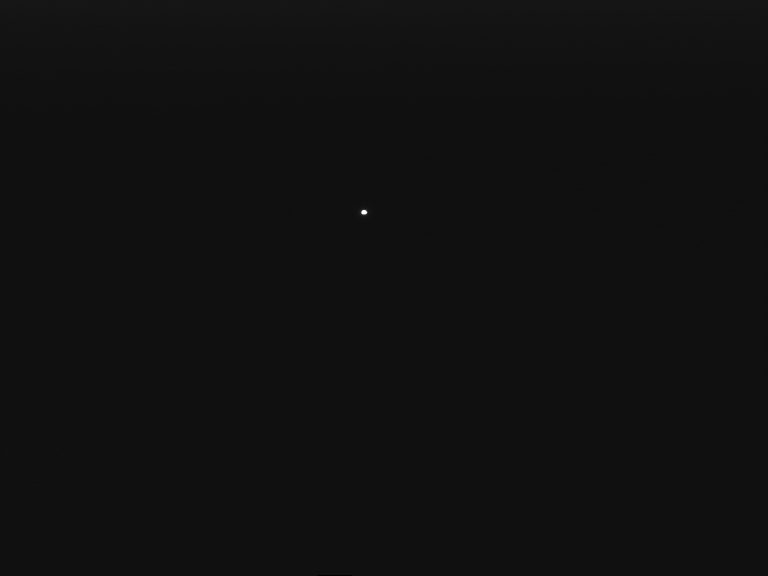

Supplement: S1 Dataset — This archive contains the captured data files used as the basis for the P4P solutions described in the manuscript. The data are provided in a directory hierarchy where each degree of freedom has a separate directory. And the calibration data is the captured data used in the camera calibration. (ZIP) [file pone.0134029.s001.zip › S1_Dataset/Pitch Angle/(15,0,0,0).tif]

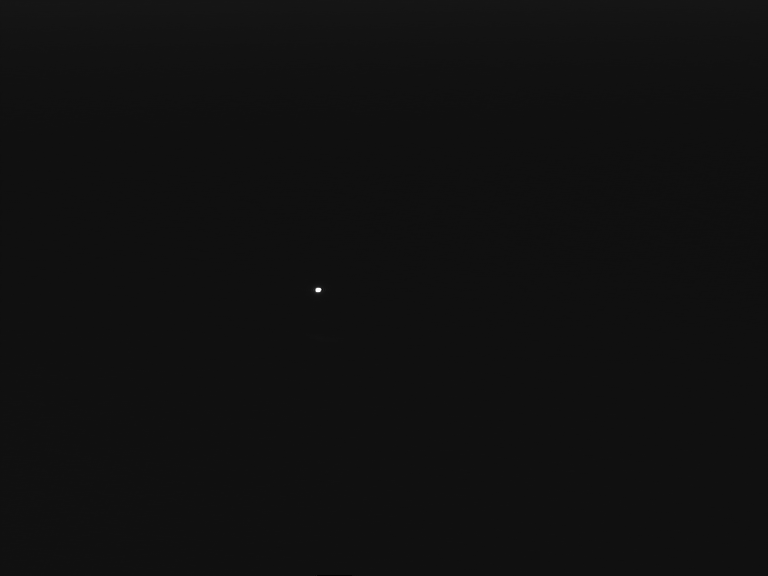

Supplement: S1 Dataset — This archive contains the captured data files used as the basis for the P4P solutions described in the manuscript. The data are provided in a directory hierarchy where each degree of freedom has a separate directory. And the calibration data is the captured data used in the camera calibration. (ZIP) [file pone.0134029.s001.zip › S1_Dataset/Pitch Angle/(15,0,0,1).tif]

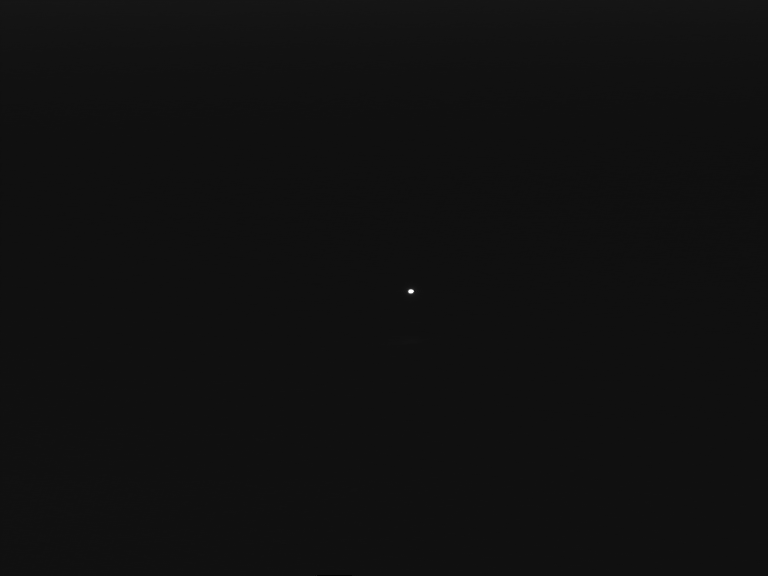

Supplement: S1 Dataset — This archive contains the captured data files used as the basis for the P4P solutions described in the manuscript. The data are provided in a directory hierarchy where each degree of freedom has a separate directory. And the calibration data is the captured data used in the camera calibration. (ZIP) [file pone.0134029.s001.zip › S1_Dataset/Pitch Angle/(15,0,0,2).tif]

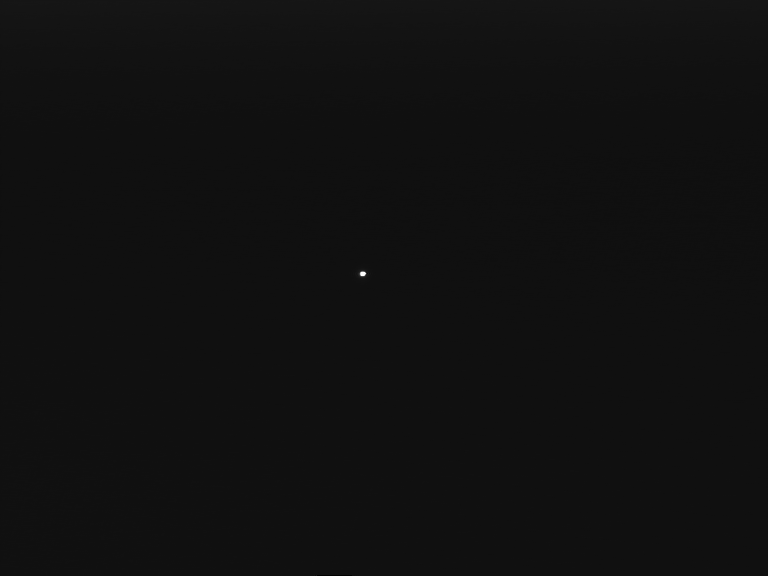

Supplement: S1 Dataset — This archive contains the captured data files used as the basis for the P4P solutions described in the manuscript. The data are provided in a directory hierarchy where each degree of freedom has a separate directory. And the calibration data is the captured data used in the camera calibration. (ZIP) [file pone.0134029.s001.zip › S1_Dataset/Pitch Angle/(15,0,0,3).tif]

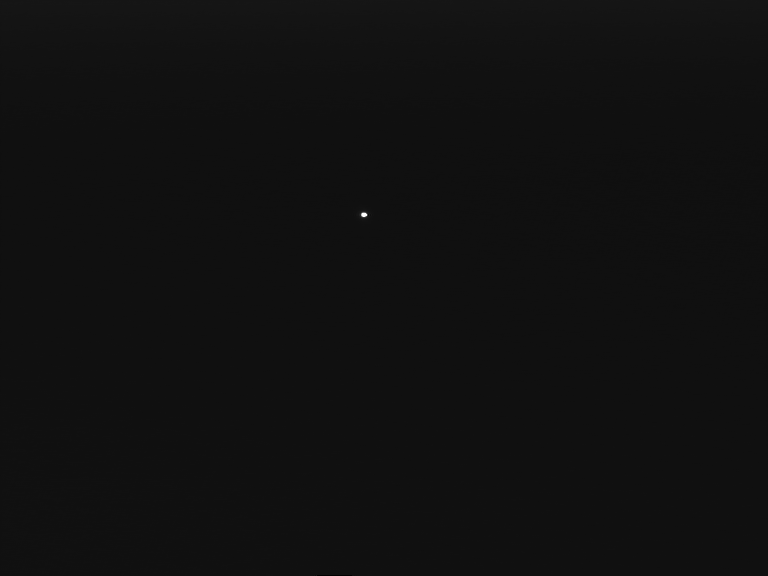

Supplement: S1 Dataset — This archive contains the captured data files used as the basis for the P4P solutions described in the manuscript. The data are provided in a directory hierarchy where each degree of freedom has a separate directory. And the calibration data is the captured data used in the camera calibration. (ZIP) [file pone.0134029.s001.zip › S1_Dataset/Pitch Angle/(16,0,0,0).tif]

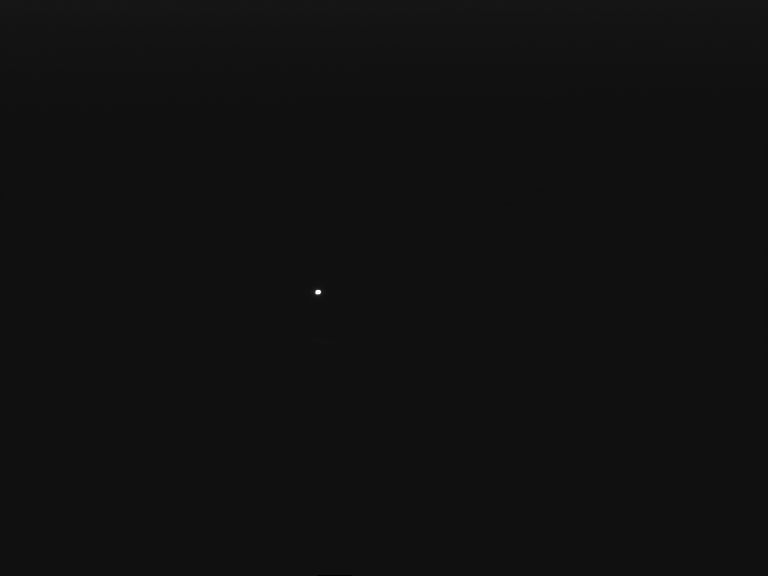

Supplement: S1 Dataset — This archive contains the captured data files used as the basis for the P4P solutions described in the manuscript. The data are provided in a directory hierarchy where each degree of freedom has a separate directory. And the calibration data is the captured data used in the camera calibration. (ZIP) [file pone.0134029.s001.zip › S1_Dataset/Pitch Angle/(16,0,0,1).tif]

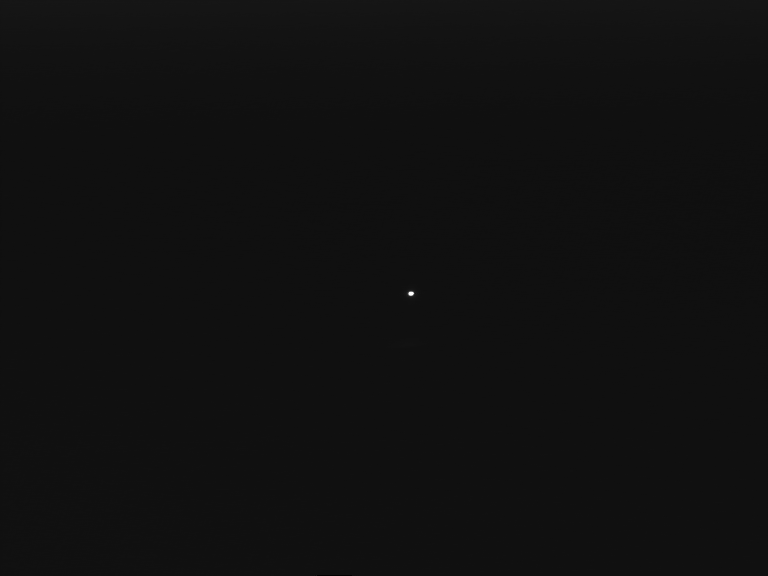

Supplement: S1 Dataset — This archive contains the captured data files used as the basis for the P4P solutions described in the manuscript. The data are provided in a directory hierarchy where each degree of freedom has a separate directory. And the calibration data is the captured data used in the camera calibration. (ZIP) [file pone.0134029.s001.zip › S1_Dataset/Pitch Angle/(16,0,0,2).tif]

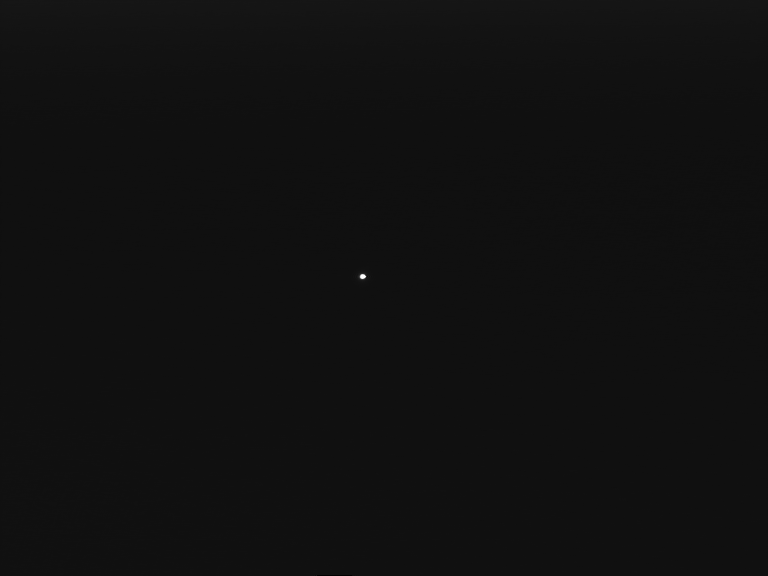

Supplement: S1 Dataset — This archive contains the captured data files used as the basis for the P4P solutions described in the manuscript. The data are provided in a directory hierarchy where each degree of freedom has a separate directory. And the calibration data is the captured data used in the camera calibration. (ZIP) [file pone.0134029.s001.zip › S1_Dataset/Pitch Angle/(16,0,0,3).tif]

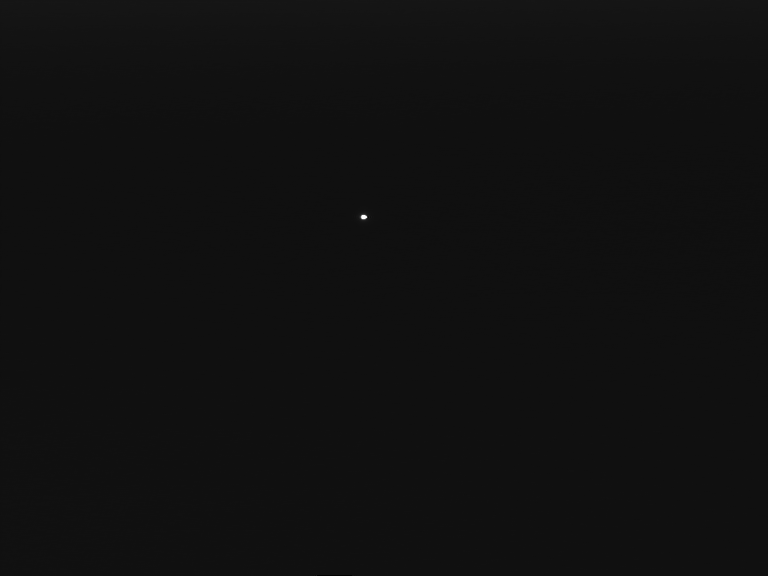

Supplement: S1 Dataset — This archive contains the captured data files used as the basis for the P4P solutions described in the manuscript. The data are provided in a directory hierarchy where each degree of freedom has a separate directory. And the calibration data is the captured data used in the camera calibration. (ZIP) [file pone.0134029.s001.zip › S1_Dataset/Pitch Angle/(17,0,0,0).tif]

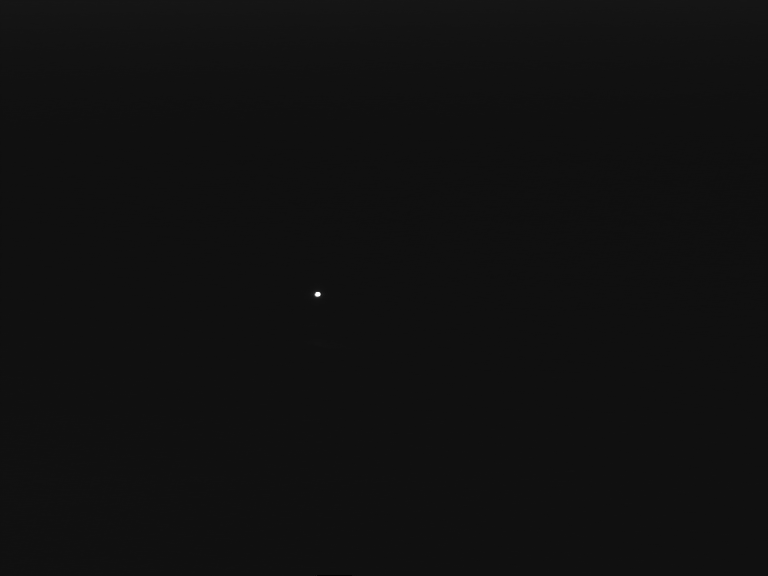

Supplement: S1 Dataset — This archive contains the captured data files used as the basis for the P4P solutions described in the manuscript. The data are provided in a directory hierarchy where each degree of freedom has a separate directory. And the calibration data is the captured data used in the camera calibration. (ZIP) [file pone.0134029.s001.zip › S1_Dataset/Pitch Angle/(17,0,0,1).tif]

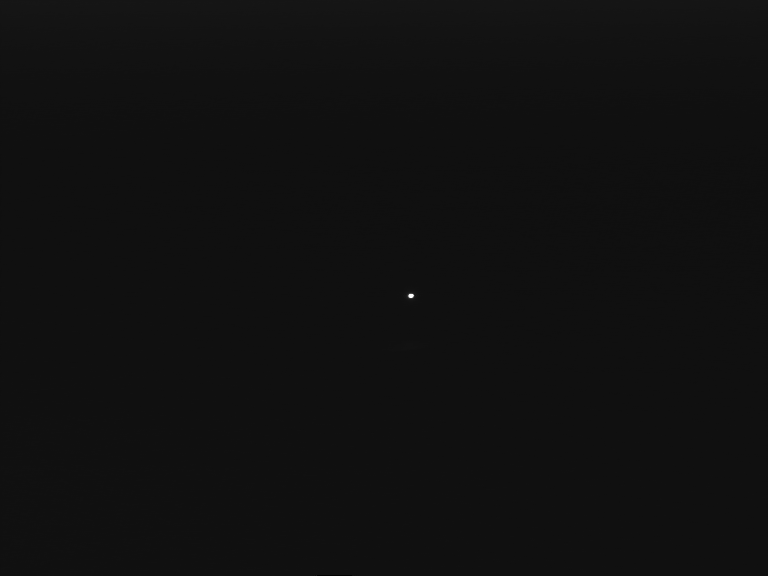

Supplement: S1 Dataset — This archive contains the captured data files used as the basis for the P4P solutions described in the manuscript. The data are provided in a directory hierarchy where each degree of freedom has a separate directory. And the calibration data is the captured data used in the camera calibration. (ZIP) [file pone.0134029.s001.zip › S1_Dataset/Pitch Angle/(17,0,0,2).tif]

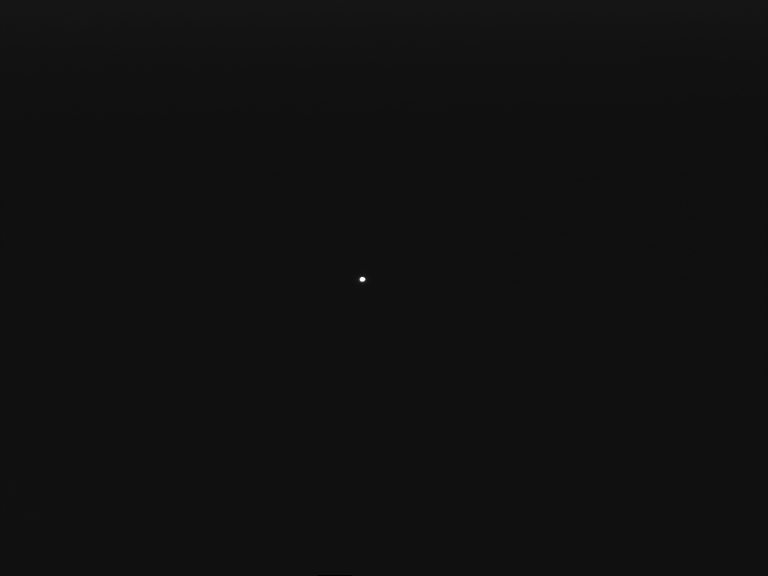

Supplement: S1 Dataset — This archive contains the captured data files used as the basis for the P4P solutions described in the manuscript. The data are provided in a directory hierarchy where each degree of freedom has a separate directory. And the calibration data is the captured data used in the camera calibration. (ZIP) [file pone.0134029.s001.zip › S1_Dataset/Pitch Angle/(17,0,0,3).tif]

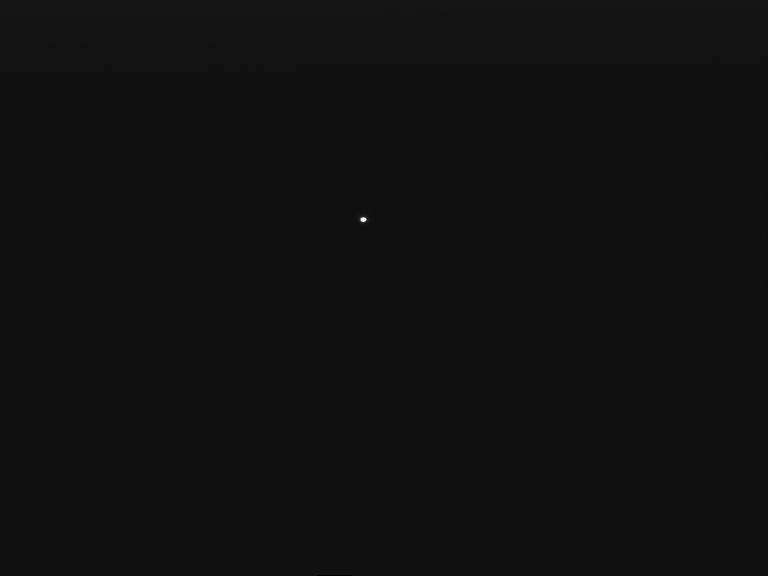

Supplement: S1 Dataset — This archive contains the captured data files used as the basis for the P4P solutions described in the manuscript. The data are provided in a directory hierarchy where each degree of freedom has a separate directory. And the calibration data is the captured data used in the camera calibration. (ZIP) [file pone.0134029.s001.zip › S1_Dataset/Pitch Angle/(18,0,0,0).tif]

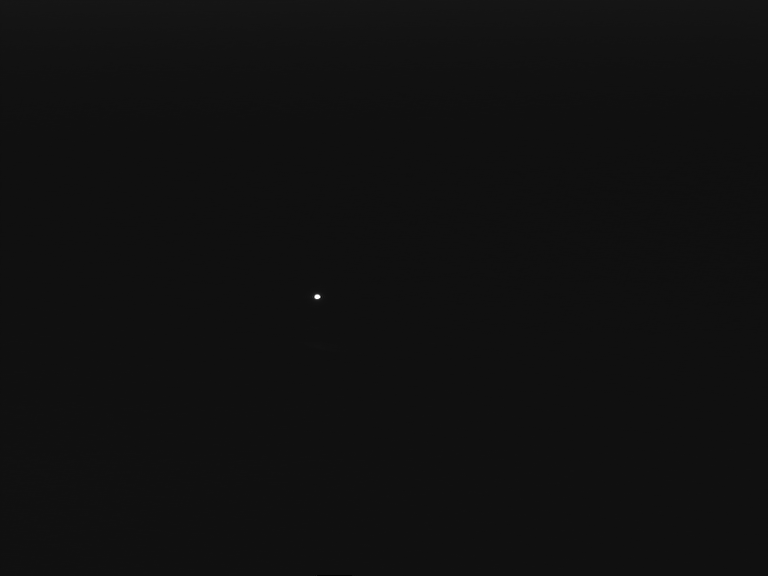

Supplement: S1 Dataset — This archive contains the captured data files used as the basis for the P4P solutions described in the manuscript. The data are provided in a directory hierarchy where each degree of freedom has a separate directory. And the calibration data is the captured data used in the camera calibration. (ZIP) [file pone.0134029.s001.zip › S1_Dataset/Pitch Angle/(18,0,0,1).tif]

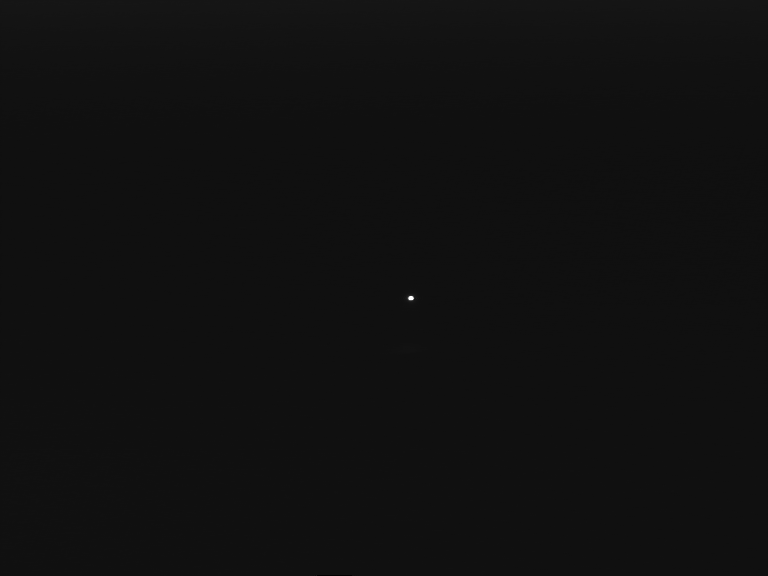

Supplement: S1 Dataset — This archive contains the captured data files used as the basis for the P4P solutions described in the manuscript. The data are provided in a directory hierarchy where each degree of freedom has a separate directory. And the calibration data is the captured data used in the camera calibration. (ZIP) [file pone.0134029.s001.zip › S1_Dataset/Pitch Angle/(18,0,0,2).tif]

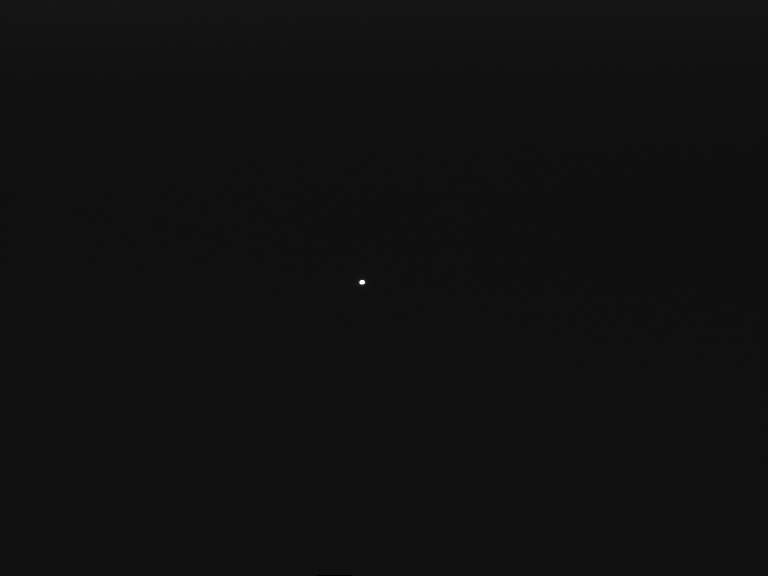

Supplement: S1 Dataset — This archive contains the captured data files used as the basis for the P4P solutions described in the manuscript. The data are provided in a directory hierarchy where each degree of freedom has a separate directory. And the calibration data is the captured data used in the camera calibration. (ZIP) [file pone.0134029.s001.zip › S1_Dataset/Pitch Angle/(18,0,0,3).tif]

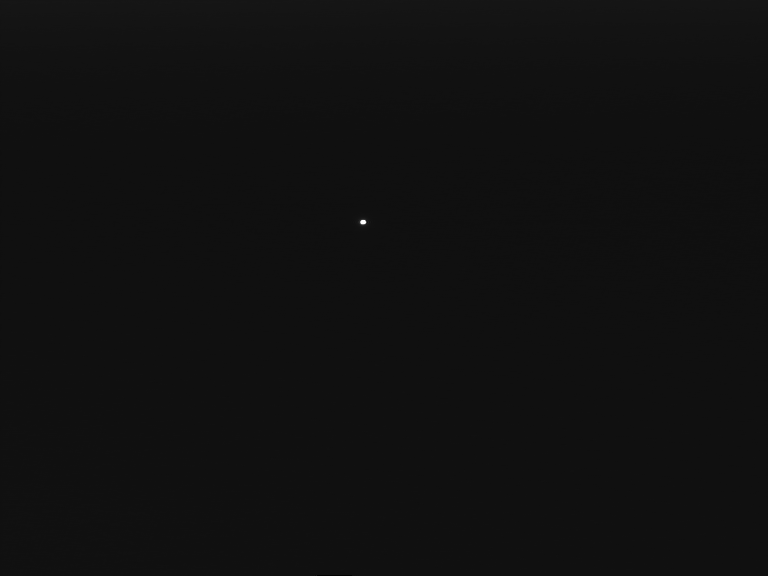

Supplement: S1 Dataset — This archive contains the captured data files used as the basis for the P4P solutions described in the manuscript. The data are provided in a directory hierarchy where each degree of freedom has a separate directory. And the calibration data is the captured data used in the camera calibration. (ZIP) [file pone.0134029.s001.zip › S1_Dataset/Pitch Angle/(19,0,0,0).tif]

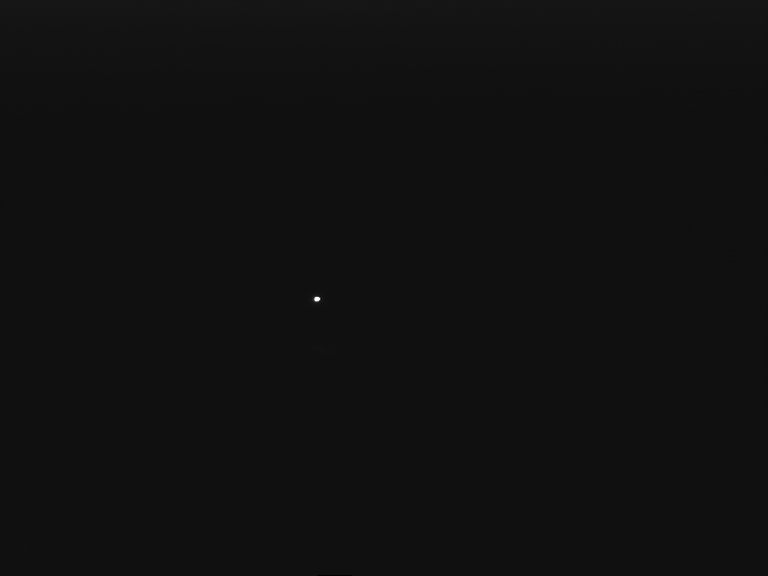

Supplement: S1 Dataset — This archive contains the captured data files used as the basis for the P4P solutions described in the manuscript. The data are provided in a directory hierarchy where each degree of freedom has a separate directory. And the calibration data is the captured data used in the camera calibration. (ZIP) [file pone.0134029.s001.zip › S1_Dataset/Pitch Angle/(19,0,0,1).tif]

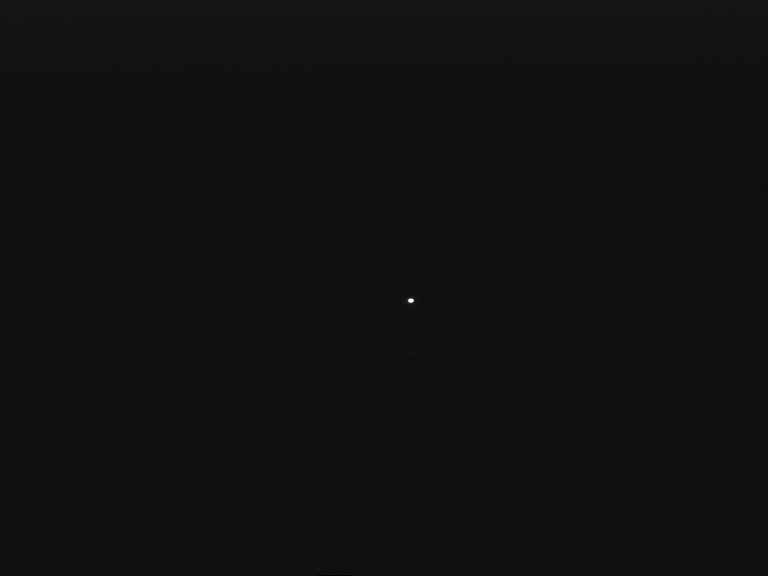

Supplement: S1 Dataset — This archive contains the captured data files used as the basis for the P4P solutions described in the manuscript. The data are provided in a directory hierarchy where each degree of freedom has a separate directory. And the calibration data is the captured data used in the camera calibration. (ZIP) [file pone.0134029.s001.zip › S1_Dataset/Pitch Angle/(19,0,0,2).tif]

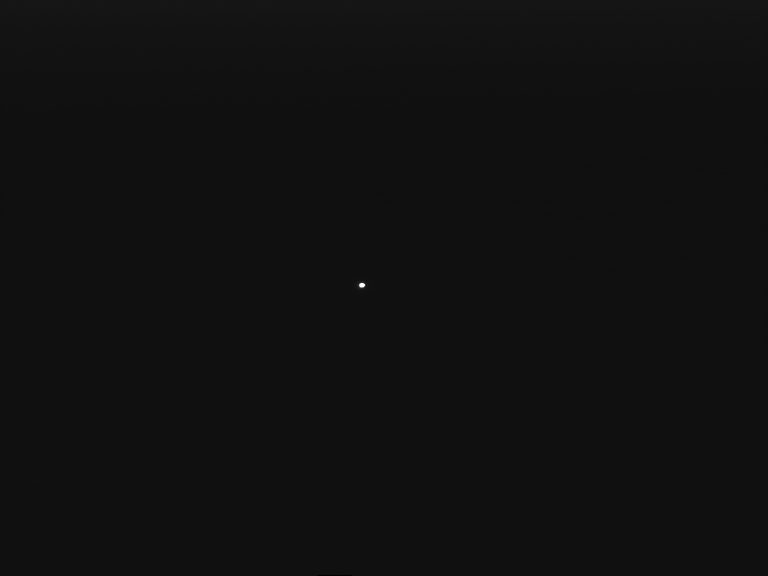

Supplement: S1 Dataset — This archive contains the captured data files used as the basis for the P4P solutions described in the manuscript. The data are provided in a directory hierarchy where each degree of freedom has a separate directory. And the calibration data is the captured data used in the camera calibration. (ZIP) [file pone.0134029.s001.zip › S1_Dataset/Pitch Angle/(19,0,0,3).tif]

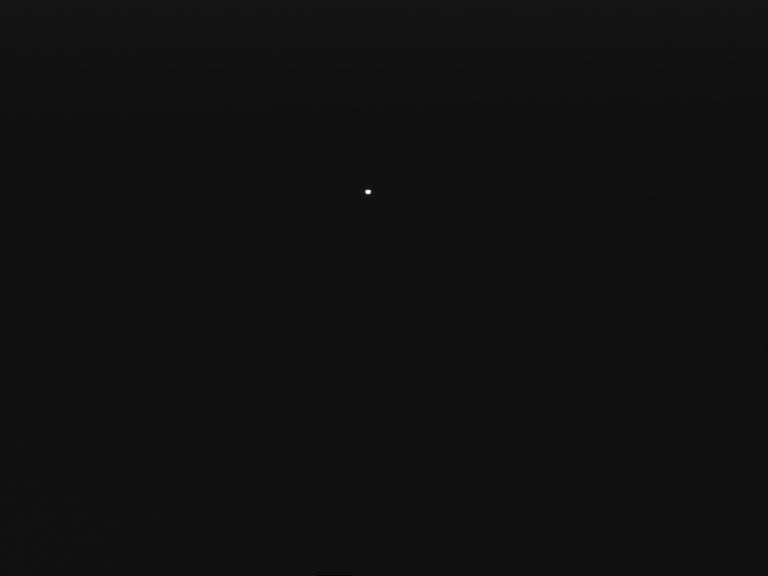

Supplement: S1 Dataset — This archive contains the captured data files used as the basis for the P4P solutions described in the manuscript. The data are provided in a directory hierarchy where each degree of freedom has a separate directory. And the calibration data is the captured data used in the camera calibration. (ZIP) [file pone.0134029.s001.zip › S1_Dataset/Pitch Angle/(2,0,0,0).tif]

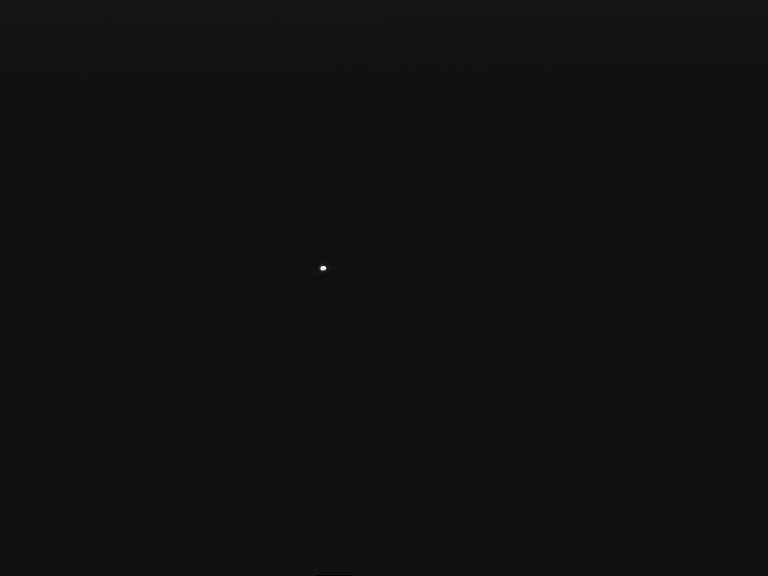

Supplement: S1 Dataset — This archive contains the captured data files used as the basis for the P4P solutions described in the manuscript. The data are provided in a directory hierarchy where each degree of freedom has a separate directory. And the calibration data is the captured data used in the camera calibration. (ZIP) [file pone.0134029.s001.zip › S1_Dataset/Pitch Angle/(2,0,0,1).tif]

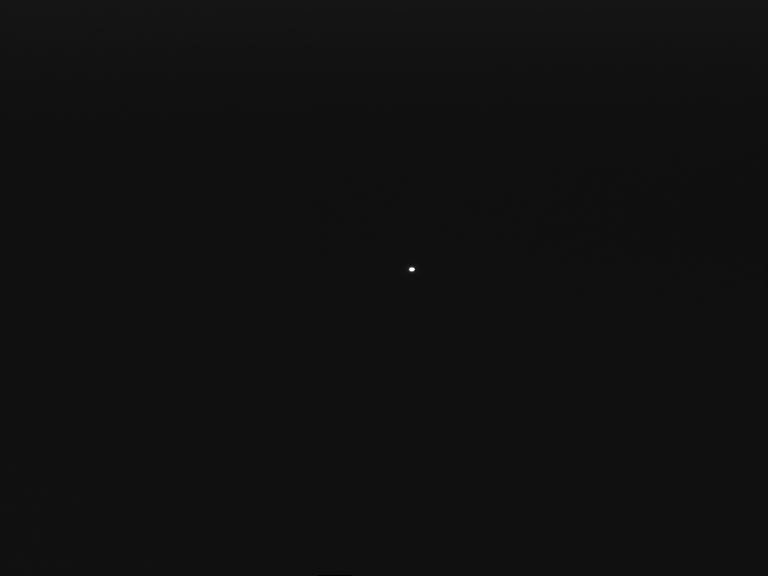

Supplement: S1 Dataset — This archive contains the captured data files used as the basis for the P4P solutions described in the manuscript. The data are provided in a directory hierarchy where each degree of freedom has a separate directory. And the calibration data is the captured data used in the camera calibration. (ZIP) [file pone.0134029.s001.zip › S1_Dataset/Pitch Angle/(2,0,0,2).tif]

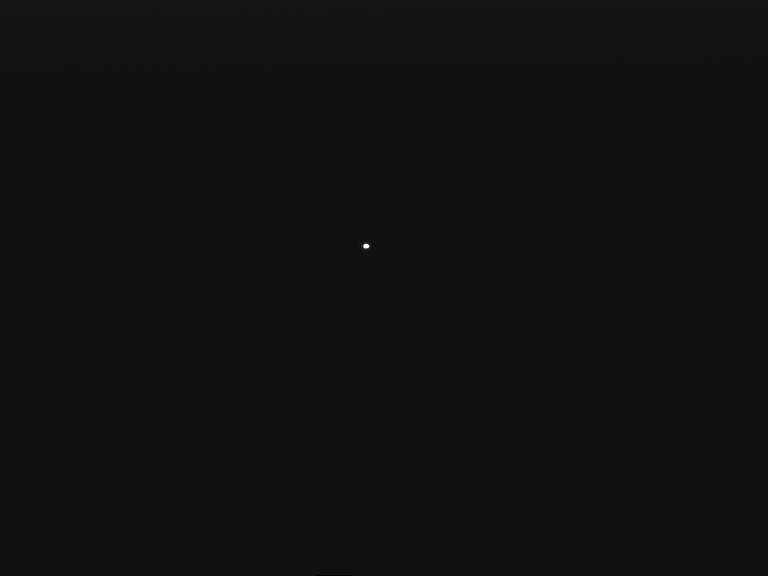

Supplement: S1 Dataset — This archive contains the captured data files used as the basis for the P4P solutions described in the manuscript. The data are provided in a directory hierarchy where each degree of freedom has a separate directory. And the calibration data is the captured data used in the camera calibration. (ZIP) [file pone.0134029.s001.zip › S1_Dataset/Pitch Angle/(2,0,0,3).tif]

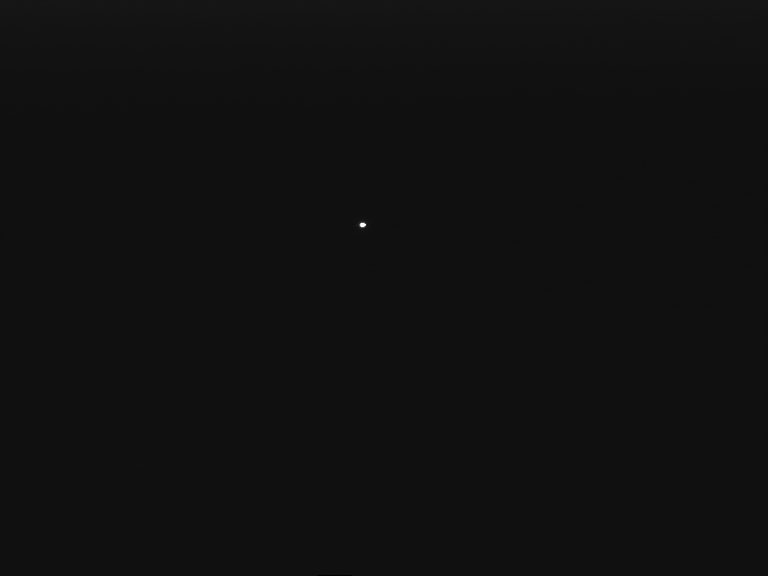

Supplement: S1 Dataset — This archive contains the captured data files used as the basis for the P4P solutions described in the manuscript. The data are provided in a directory hierarchy where each degree of freedom has a separate directory. And the calibration data is the captured data used in the camera calibration. (ZIP) [file pone.0134029.s001.zip › S1_Dataset/Pitch Angle/(20,0,0,0).tif]

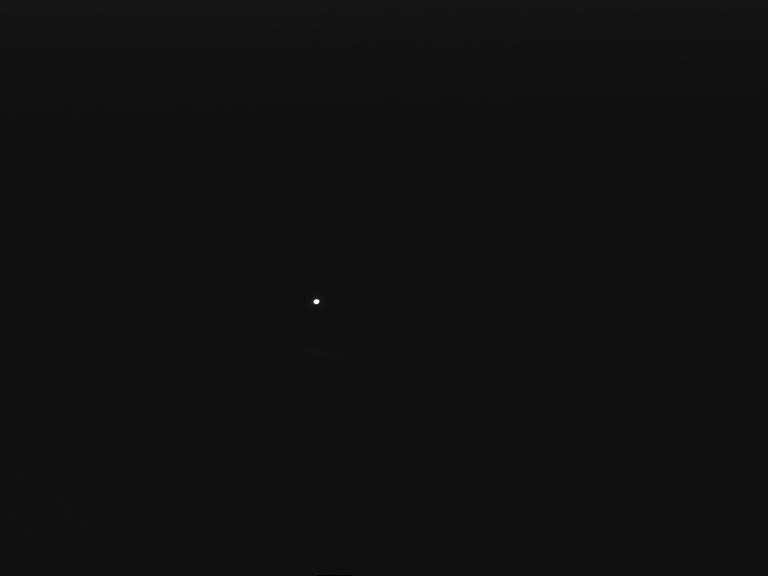

Supplement: S1 Dataset — This archive contains the captured data files used as the basis for the P4P solutions described in the manuscript. The data are provided in a directory hierarchy where each degree of freedom has a separate directory. And the calibration data is the captured data used in the camera calibration. (ZIP) [file pone.0134029.s001.zip › S1_Dataset/Pitch Angle/(20,0,0,1).tif]

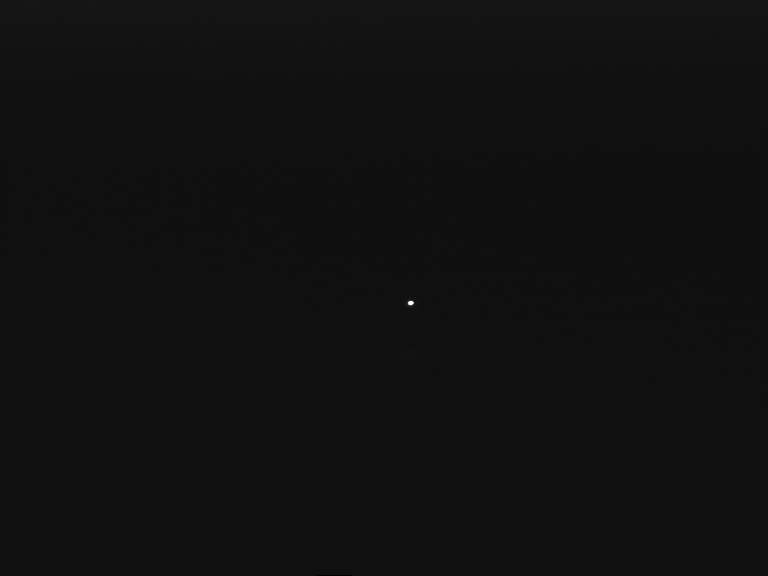

Supplement: S1 Dataset — This archive contains the captured data files used as the basis for the P4P solutions described in the manuscript. The data are provided in a directory hierarchy where each degree of freedom has a separate directory. And the calibration data is the captured data used in the camera calibration. (ZIP) [file pone.0134029.s001.zip › S1_Dataset/Pitch Angle/(20,0,0,2).tif]

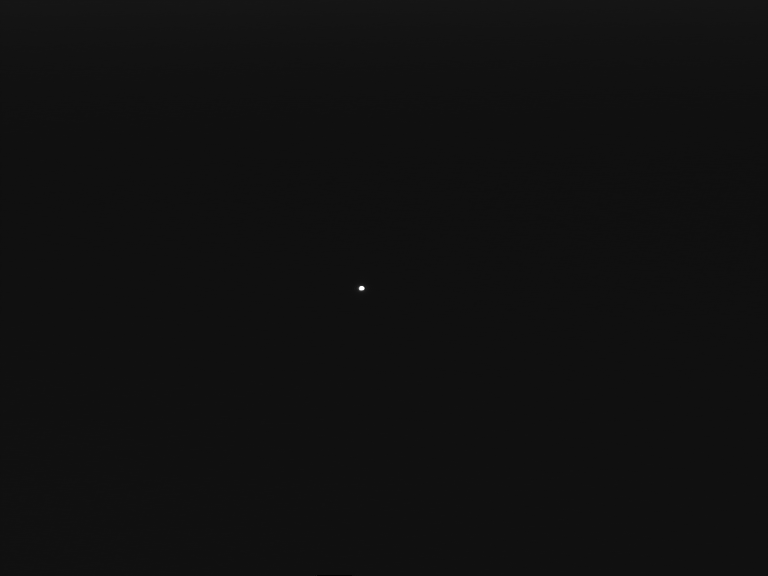

Supplement: S1 Dataset — This archive contains the captured data files used as the basis for the P4P solutions described in the manuscript. The data are provided in a directory hierarchy where each degree of freedom has a separate directory. And the calibration data is the captured data used in the camera calibration. (ZIP) [file pone.0134029.s001.zip › S1_Dataset/Pitch Angle/(20,0,0,3).tif]

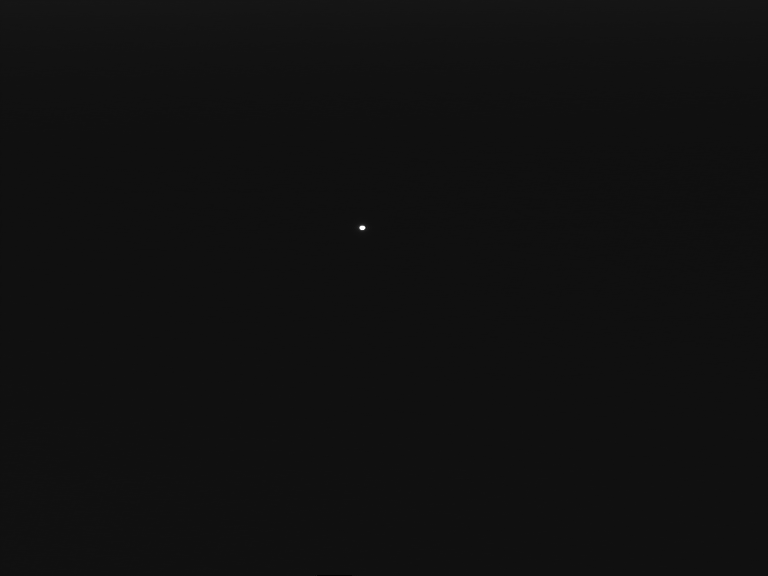

Supplement: S1 Dataset — This archive contains the captured data files used as the basis for the P4P solutions described in the manuscript. The data are provided in a directory hierarchy where each degree of freedom has a separate directory. And the calibration data is the captured data used in the camera calibration. (ZIP) [file pone.0134029.s001.zip › S1_Dataset/Pitch Angle/(21,0,0,0).tif]

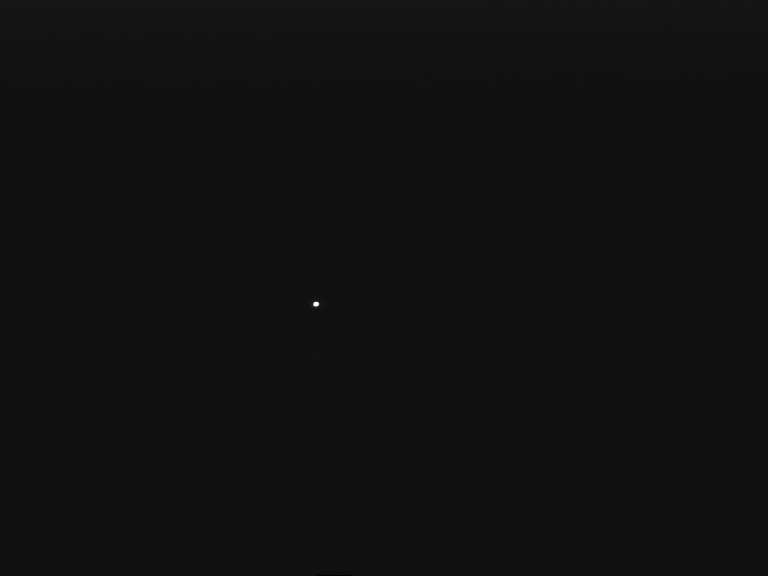

Supplement: S1 Dataset — This archive contains the captured data files used as the basis for the P4P solutions described in the manuscript. The data are provided in a directory hierarchy where each degree of freedom has a separate directory. And the calibration data is the captured data used in the camera calibration. (ZIP) [file pone.0134029.s001.zip › S1_Dataset/Pitch Angle/(21,0,0,1).tif]

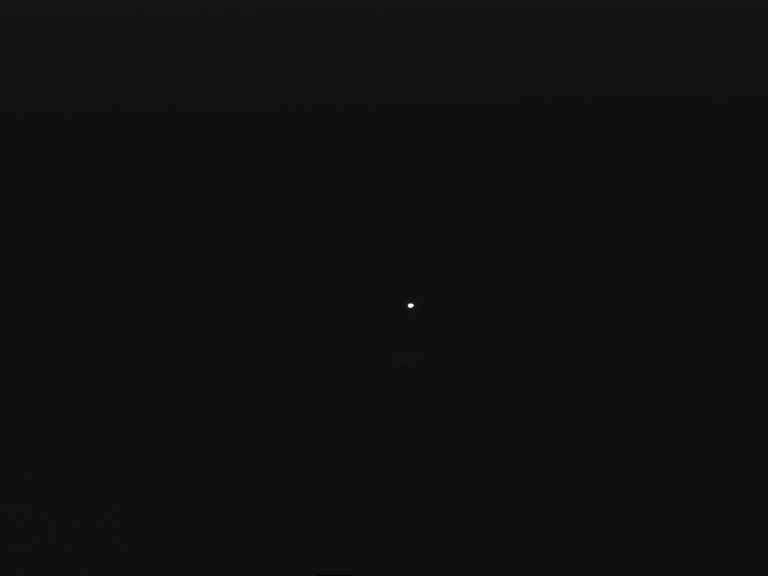

Supplement: S1 Dataset — This archive contains the captured data files used as the basis for the P4P solutions described in the manuscript. The data are provided in a directory hierarchy where each degree of freedom has a separate directory. And the calibration data is the captured data used in the camera calibration. (ZIP) [file pone.0134029.s001.zip › S1_Dataset/Pitch Angle/(21,0,0,2).tif]

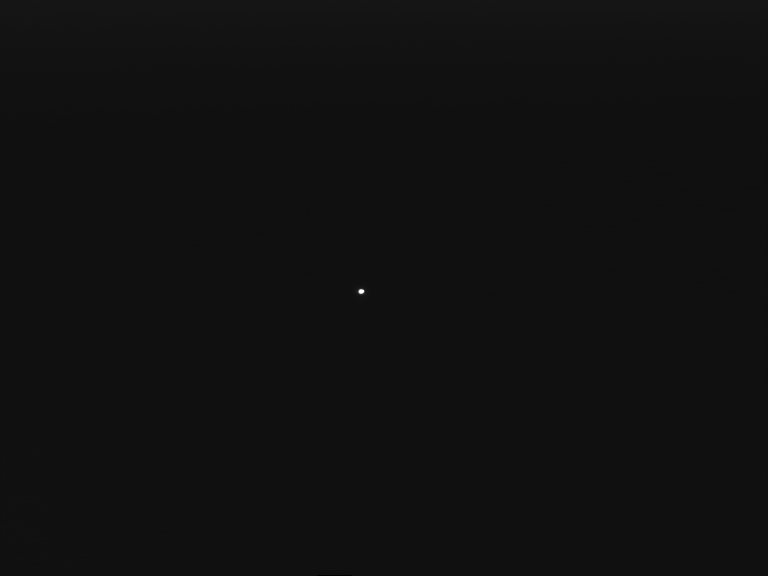

Supplement: S1 Dataset — This archive contains the captured data files used as the basis for the P4P solutions described in the manuscript. The data are provided in a directory hierarchy where each degree of freedom has a separate directory. And the calibration data is the captured data used in the camera calibration. (ZIP) [file pone.0134029.s001.zip › S1_Dataset/Pitch Angle/(21,0,0,3).tif]

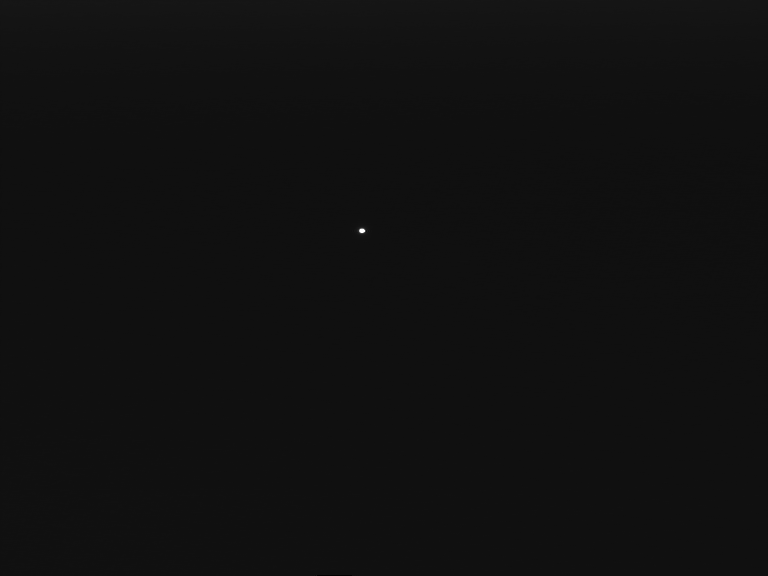

Supplement: S1 Dataset — This archive contains the captured data files used as the basis for the P4P solutions described in the manuscript. The data are provided in a directory hierarchy where each degree of freedom has a separate directory. And the calibration data is the captured data used in the camera calibration. (ZIP) [file pone.0134029.s001.zip › S1_Dataset/Pitch Angle/(22,0,0,0).tif]

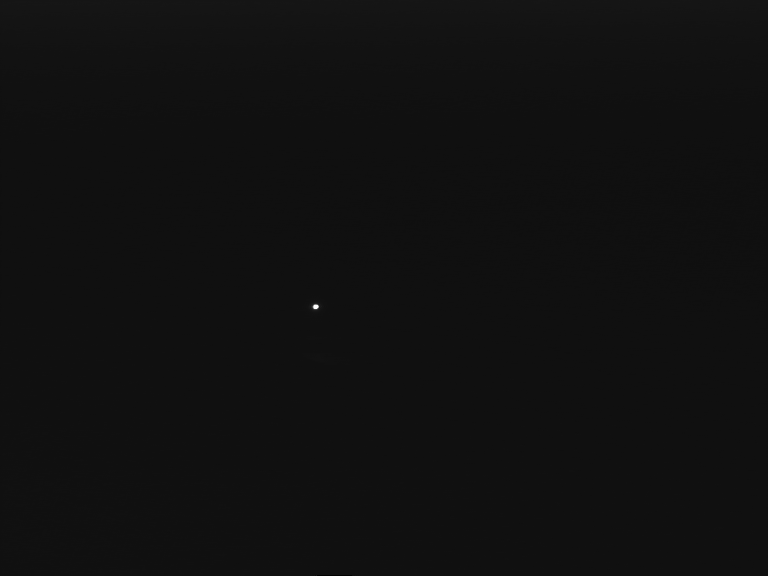

Supplement: S1 Dataset — This archive contains the captured data files used as the basis for the P4P solutions described in the manuscript. The data are provided in a directory hierarchy where each degree of freedom has a separate directory. And the calibration data is the captured data used in the camera calibration. (ZIP) [file pone.0134029.s001.zip › S1_Dataset/Pitch Angle/(22,0,0,1).tif]

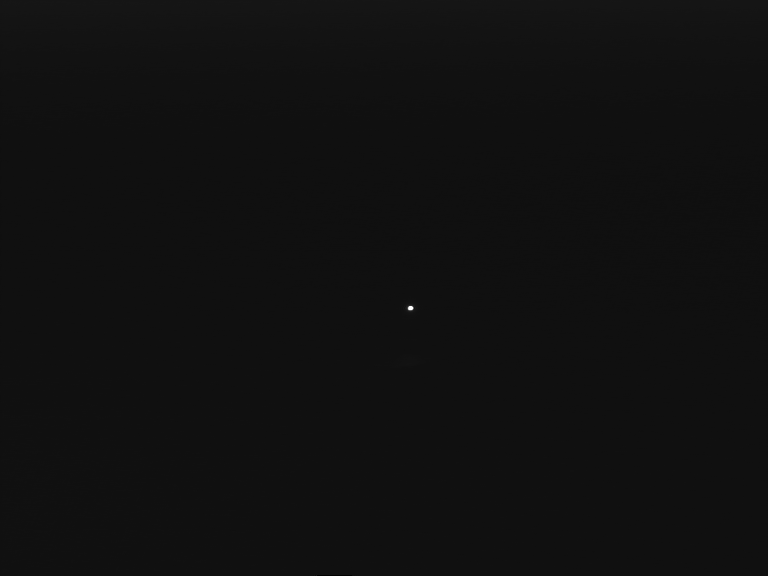

Supplement: S1 Dataset — This archive contains the captured data files used as the basis for the P4P solutions described in the manuscript. The data are provided in a directory hierarchy where each degree of freedom has a separate directory. And the calibration data is the captured data used in the camera calibration. (ZIP) [file pone.0134029.s001.zip › S1_Dataset/Pitch Angle/(22,0,0,2).tif]

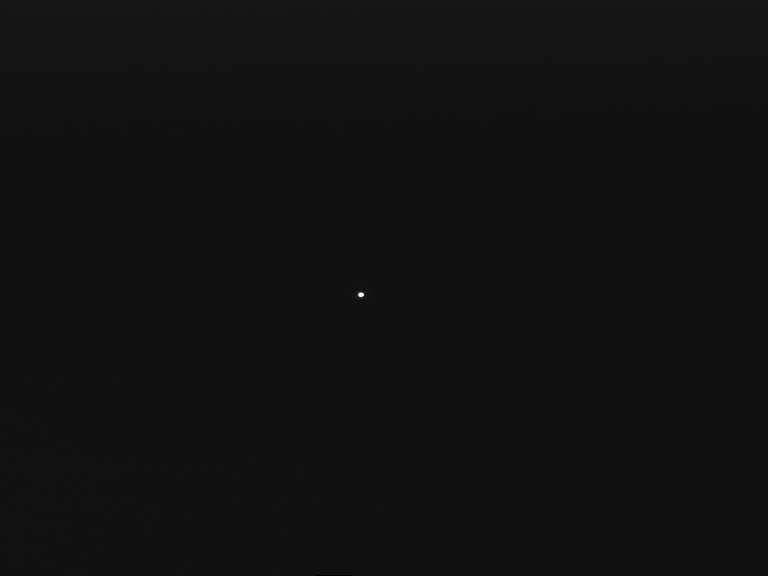

Supplement: S1 Dataset — This archive contains the captured data files used as the basis for the P4P solutions described in the manuscript. The data are provided in a directory hierarchy where each degree of freedom has a separate directory. And the calibration data is the captured data used in the camera calibration. (ZIP) [file pone.0134029.s001.zip › S1_Dataset/Pitch Angle/(22,0,0,3).tif]

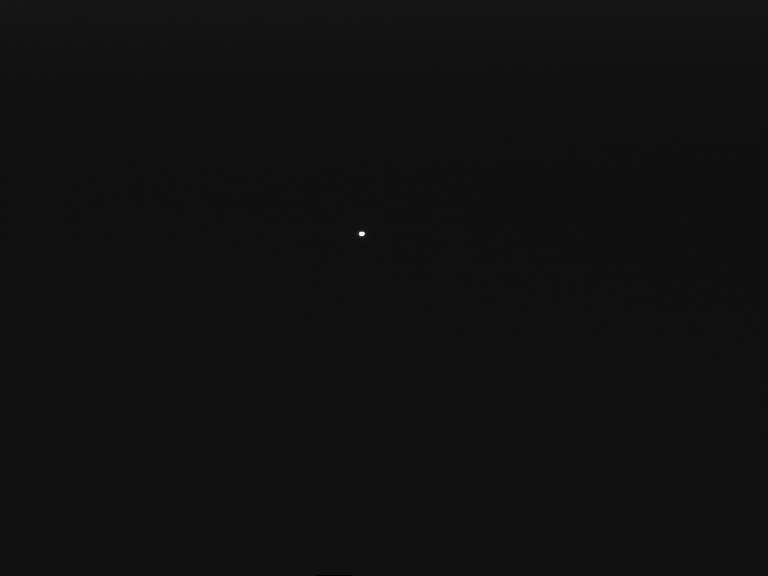

Supplement: S1 Dataset — This archive contains the captured data files used as the basis for the P4P solutions described in the manuscript. The data are provided in a directory hierarchy where each degree of freedom has a separate directory. And the calibration data is the captured data used in the camera calibration. (ZIP) [file pone.0134029.s001.zip › S1_Dataset/Pitch Angle/(23,0,0,0).tif]

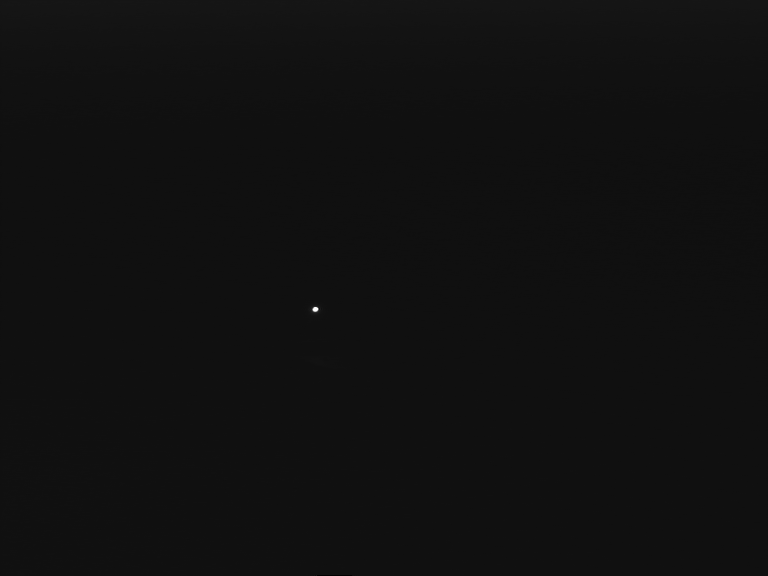

Supplement: S1 Dataset — This archive contains the captured data files used as the basis for the P4P solutions described in the manuscript. The data are provided in a directory hierarchy where each degree of freedom has a separate directory. And the calibration data is the captured data used in the camera calibration. (ZIP) [file pone.0134029.s001.zip › S1_Dataset/Pitch Angle/(23,0,0,1).tif]

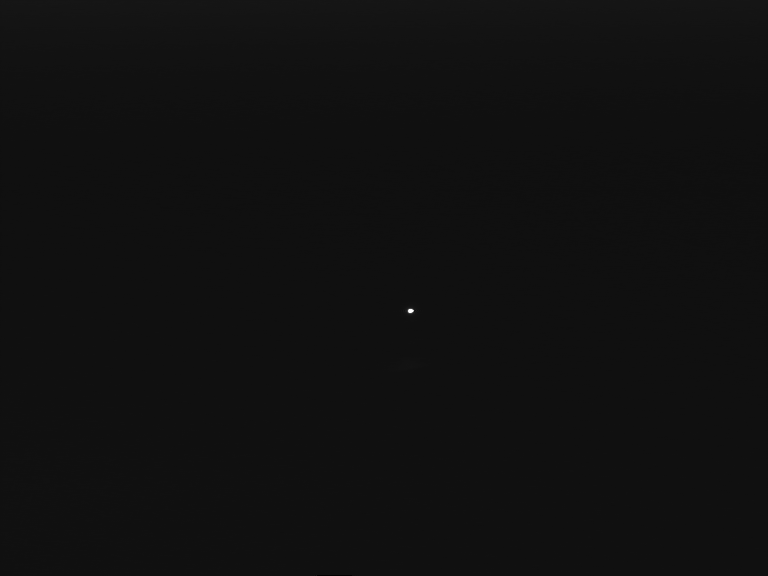

Supplement: S1 Dataset — This archive contains the captured data files used as the basis for the P4P solutions described in the manuscript. The data are provided in a directory hierarchy where each degree of freedom has a separate directory. And the calibration data is the captured data used in the camera calibration. (ZIP) [file pone.0134029.s001.zip › S1_Dataset/Pitch Angle/(23,0,0,2).tif]

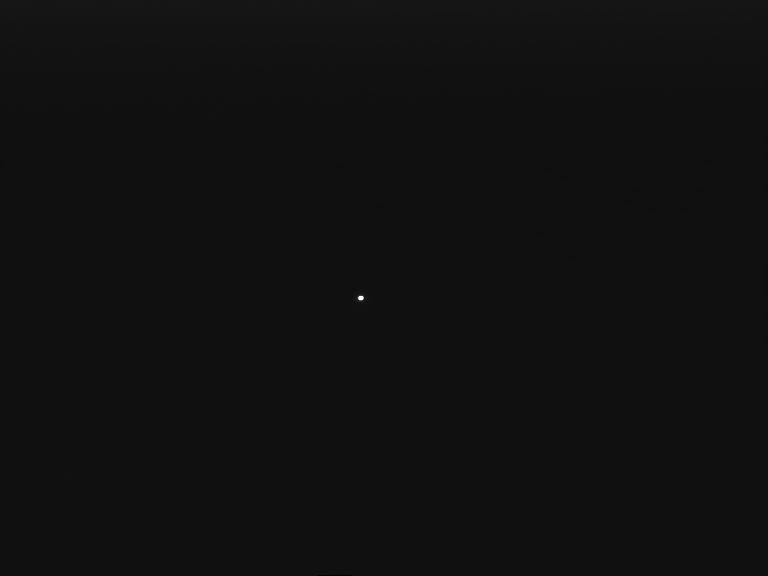

Supplement: S1 Dataset — This archive contains the captured data files used as the basis for the P4P solutions described in the manuscript. The data are provided in a directory hierarchy where each degree of freedom has a separate directory. And the calibration data is the captured data used in the camera calibration. (ZIP) [file pone.0134029.s001.zip › S1_Dataset/Pitch Angle/(23,0,0,3).tif]

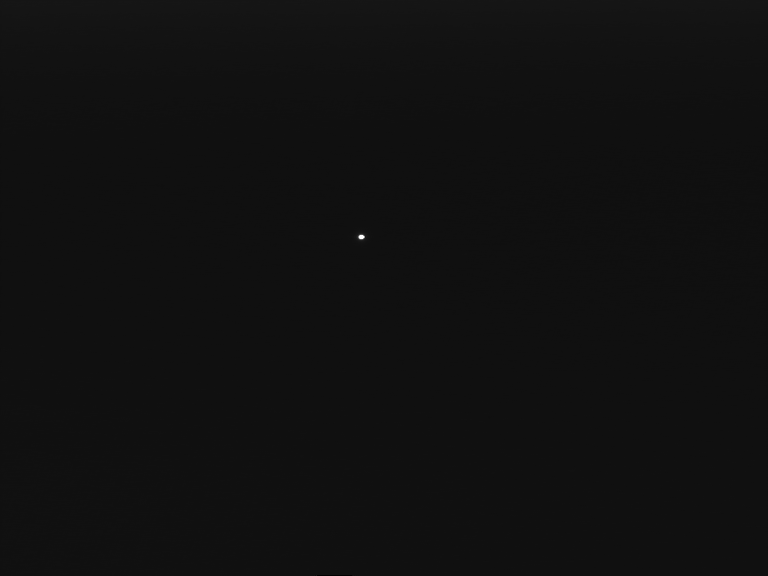

Supplement: S1 Dataset — This archive contains the captured data files used as the basis for the P4P solutions described in the manuscript. The data are provided in a directory hierarchy where each degree of freedom has a separate directory. And the calibration data is the captured data used in the camera calibration. (ZIP) [file pone.0134029.s001.zip › S1_Dataset/Pitch Angle/(24,0,0,0).tif]

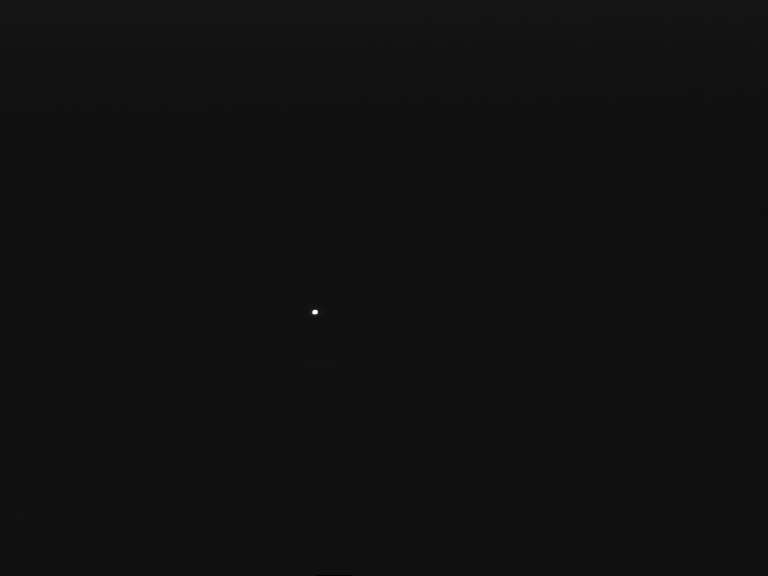

Supplement: S1 Dataset — This archive contains the captured data files used as the basis for the P4P solutions described in the manuscript. The data are provided in a directory hierarchy where each degree of freedom has a separate directory. And the calibration data is the captured data used in the camera calibration. (ZIP) [file pone.0134029.s001.zip › S1_Dataset/Pitch Angle/(24,0,0,1).tif]

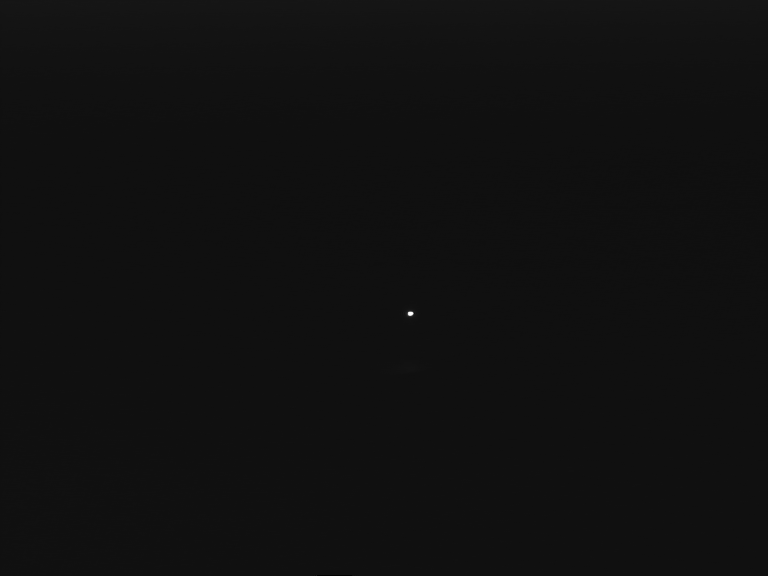

Supplement: S1 Dataset — This archive contains the captured data files used as the basis for the P4P solutions described in the manuscript. The data are provided in a directory hierarchy where each degree of freedom has a separate directory. And the calibration data is the captured data used in the camera calibration. (ZIP) [file pone.0134029.s001.zip › S1_Dataset/Pitch Angle/(24,0,0,2).tif]

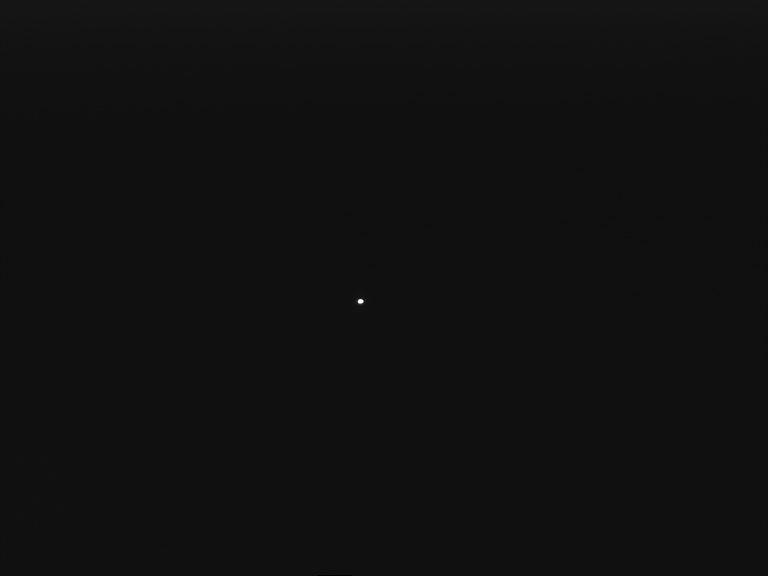

Supplement: S1 Dataset — This archive contains the captured data files used as the basis for the P4P solutions described in the manuscript. The data are provided in a directory hierarchy where each degree of freedom has a separate directory. And the calibration data is the captured data used in the camera calibration. (ZIP) [file pone.0134029.s001.zip › S1_Dataset/Pitch Angle/(24,0,0,3).tif]

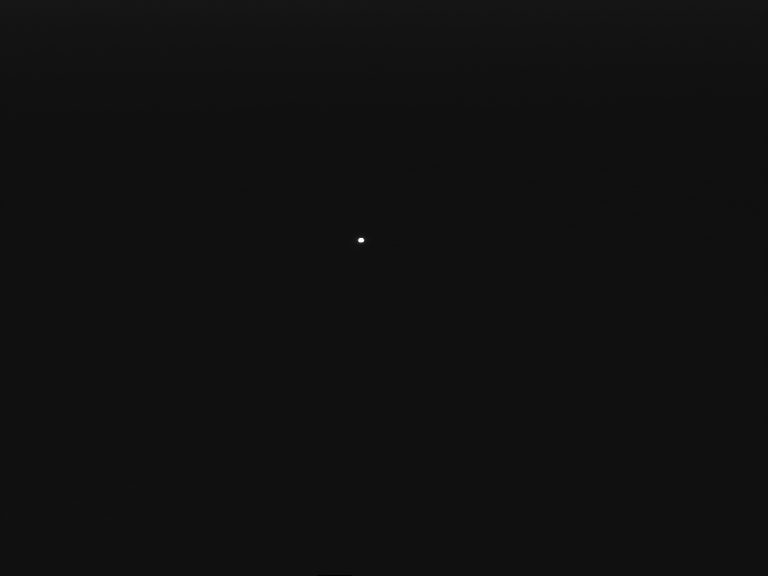

Supplement: S1 Dataset — This archive contains the captured data files used as the basis for the P4P solutions described in the manuscript. The data are provided in a directory hierarchy where each degree of freedom has a separate directory. And the calibration data is the captured data used in the camera calibration. (ZIP) [file pone.0134029.s001.zip › S1_Dataset/Pitch Angle/(25,0,0,0).tif]

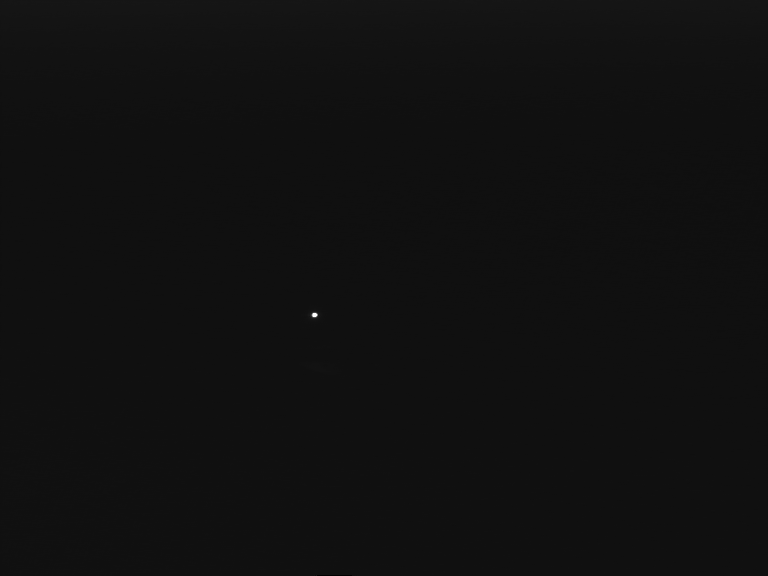

Supplement: S1 Dataset — This archive contains the captured data files used as the basis for the P4P solutions described in the manuscript. The data are provided in a directory hierarchy where each degree of freedom has a separate directory. And the calibration data is the captured data used in the camera calibration. (ZIP) [file pone.0134029.s001.zip › S1_Dataset/Pitch Angle/(25,0,0,1).tif]

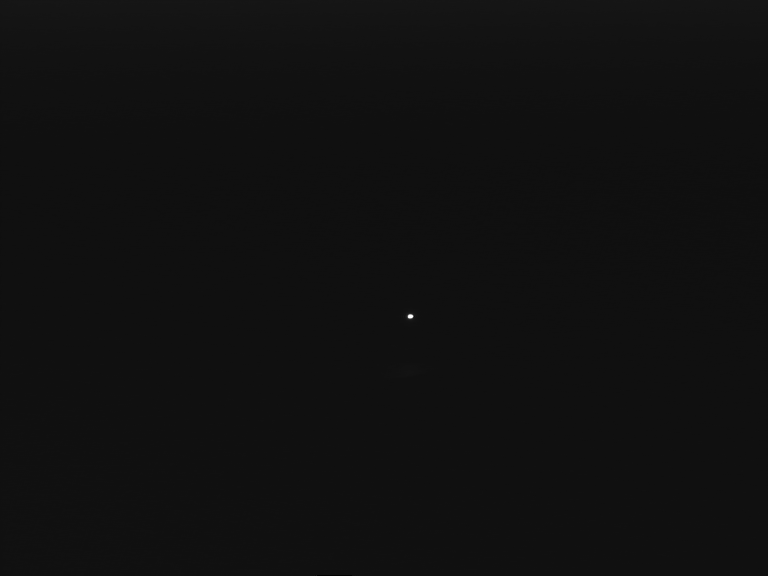

Supplement: S1 Dataset — This archive contains the captured data files used as the basis for the P4P solutions described in the manuscript. The data are provided in a directory hierarchy where each degree of freedom has a separate directory. And the calibration data is the captured data used in the camera calibration. (ZIP) [file pone.0134029.s001.zip › S1_Dataset/Pitch Angle/(25,0,0,2).tif]

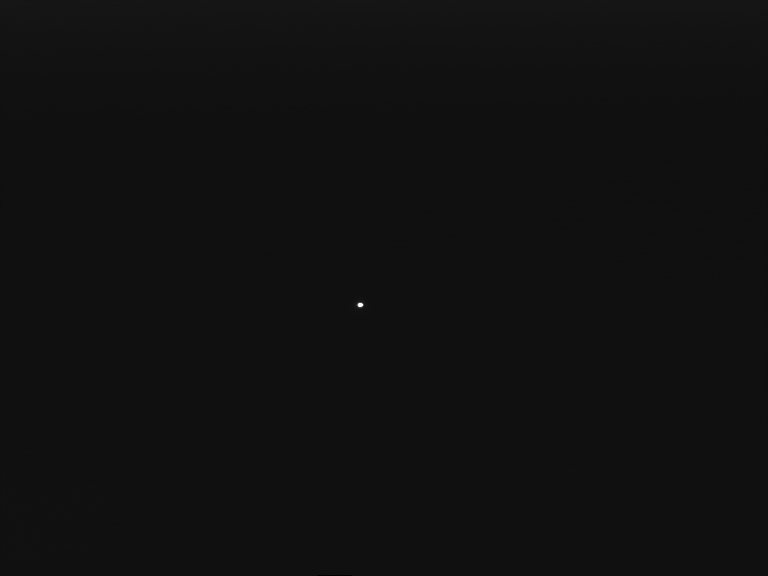

Supplement: S1 Dataset — This archive contains the captured data files used as the basis for the P4P solutions described in the manuscript. The data are provided in a directory hierarchy where each degree of freedom has a separate directory. And the calibration data is the captured data used in the camera calibration. (ZIP) [file pone.0134029.s001.zip › S1_Dataset/Pitch Angle/(25,0,0,3).tif]

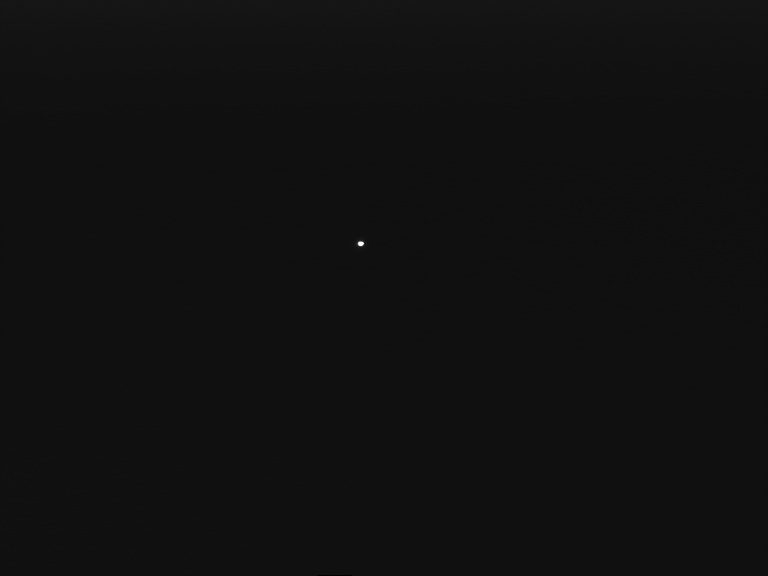

Supplement: S1 Dataset — This archive contains the captured data files used as the basis for the P4P solutions described in the manuscript. The data are provided in a directory hierarchy where each degree of freedom has a separate directory. And the calibration data is the captured data used in the camera calibration. (ZIP) [file pone.0134029.s001.zip › S1_Dataset/Pitch Angle/(26,0,0,0).tif]

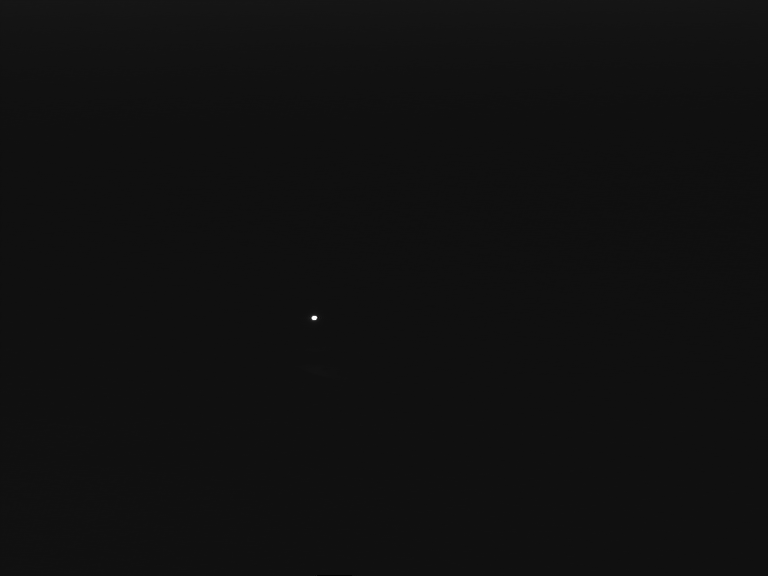

Supplement: S1 Dataset — This archive contains the captured data files used as the basis for the P4P solutions described in the manuscript. The data are provided in a directory hierarchy where each degree of freedom has a separate directory. And the calibration data is the captured data used in the camera calibration. (ZIP) [file pone.0134029.s001.zip › S1_Dataset/Pitch Angle/(26,0,0,1).tif]

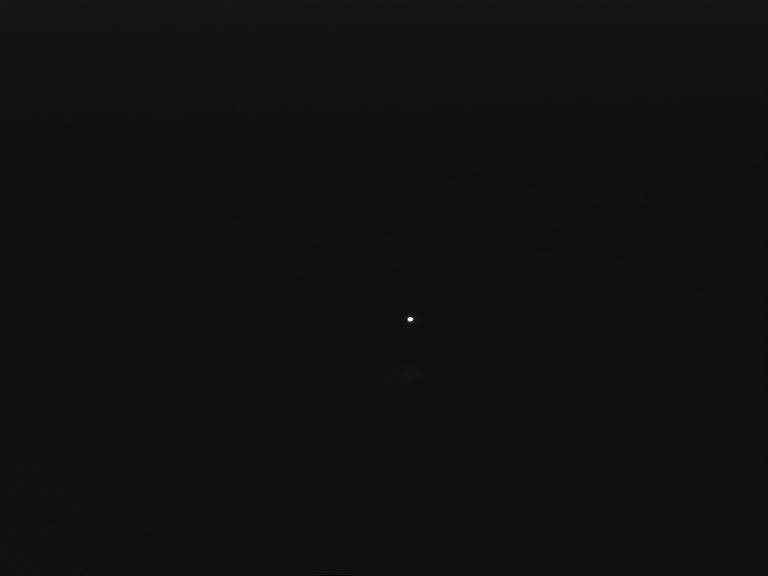

Supplement: S1 Dataset — This archive contains the captured data files used as the basis for the P4P solutions described in the manuscript. The data are provided in a directory hierarchy where each degree of freedom has a separate directory. And the calibration data is the captured data used in the camera calibration. (ZIP) [file pone.0134029.s001.zip › S1_Dataset/Pitch Angle/(26,0,0,2).tif]

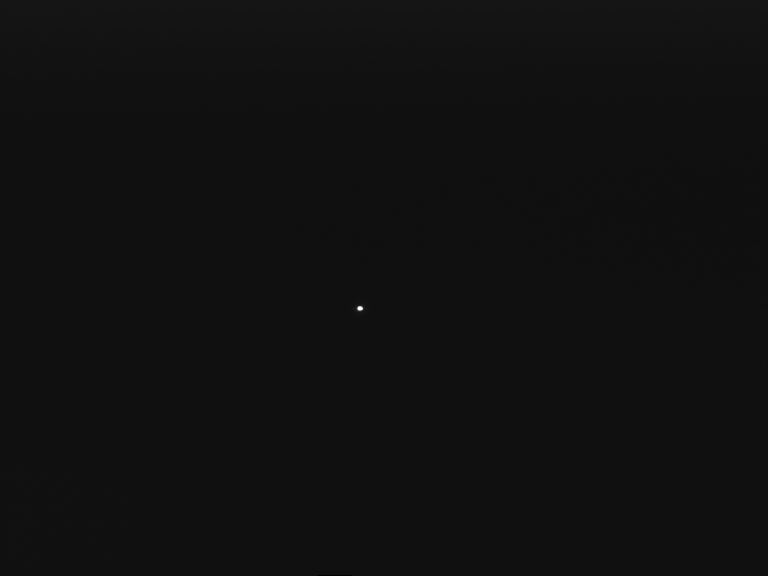

Supplement: S1 Dataset — This archive contains the captured data files used as the basis for the P4P solutions described in the manuscript. The data are provided in a directory hierarchy where each degree of freedom has a separate directory. And the calibration data is the captured data used in the camera calibration. (ZIP) [file pone.0134029.s001.zip › S1_Dataset/Pitch Angle/(26,0,0,3).tif]

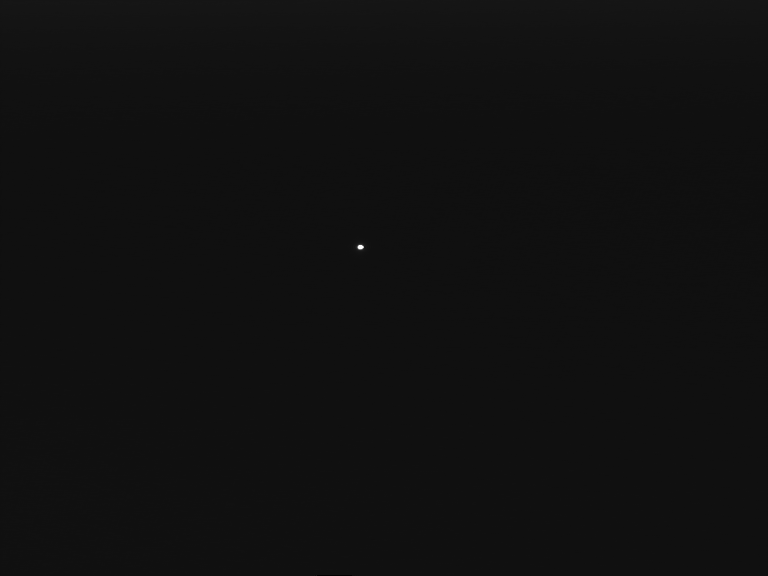

Supplement: S1 Dataset — This archive contains the captured data files used as the basis for the P4P solutions described in the manuscript. The data are provided in a directory hierarchy where each degree of freedom has a separate directory. And the calibration data is the captured data used in the camera calibration. (ZIP) [file pone.0134029.s001.zip › S1_Dataset/Pitch Angle/(27,0,0,0).tif]

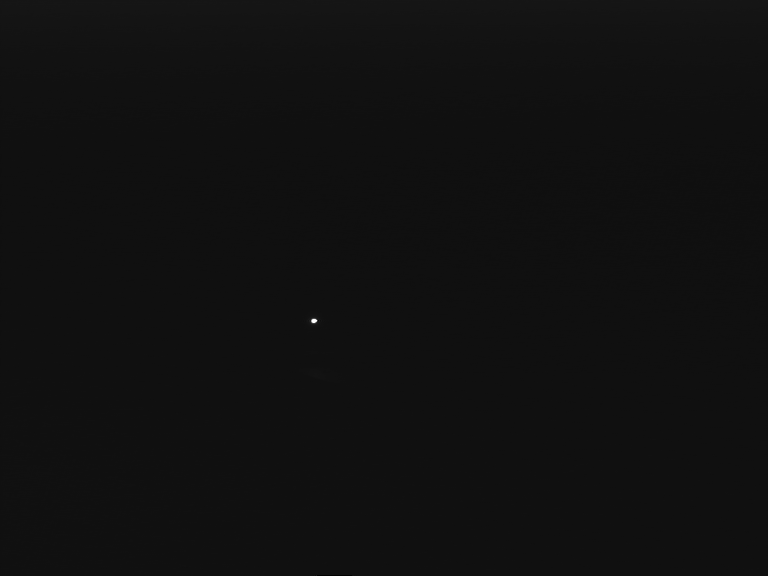

Supplement: S1 Dataset — This archive contains the captured data files used as the basis for the P4P solutions described in the manuscript. The data are provided in a directory hierarchy where each degree of freedom has a separate directory. And the calibration data is the captured data used in the camera calibration. (ZIP) [file pone.0134029.s001.zip › S1_Dataset/Pitch Angle/(27,0,0,1).tif]

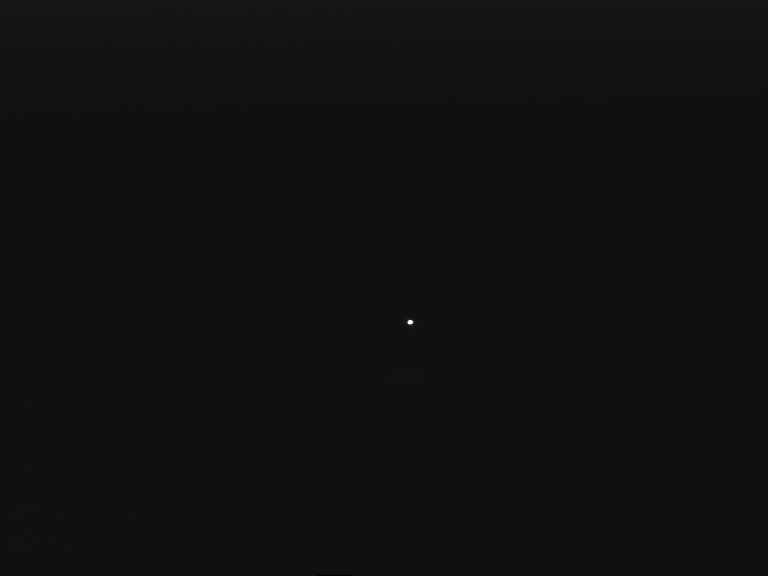

Supplement: S1 Dataset — This archive contains the captured data files used as the basis for the P4P solutions described in the manuscript. The data are provided in a directory hierarchy where each degree of freedom has a separate directory. And the calibration data is the captured data used in the camera calibration. (ZIP) [file pone.0134029.s001.zip › S1_Dataset/Pitch Angle/(27,0,0,2).tif]

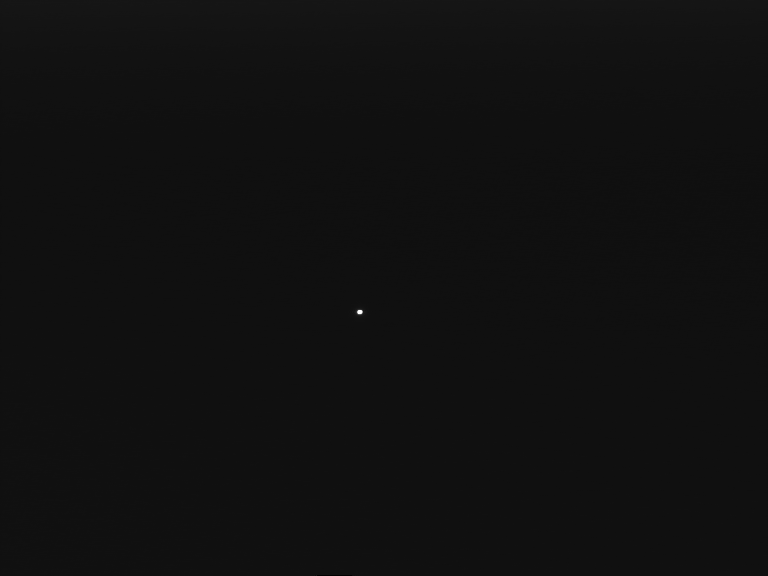

Supplement: S1 Dataset — This archive contains the captured data files used as the basis for the P4P solutions described in the manuscript. The data are provided in a directory hierarchy where each degree of freedom has a separate directory. And the calibration data is the captured data used in the camera calibration. (ZIP) [file pone.0134029.s001.zip › S1_Dataset/Pitch Angle/(27,0,0,3).tif]

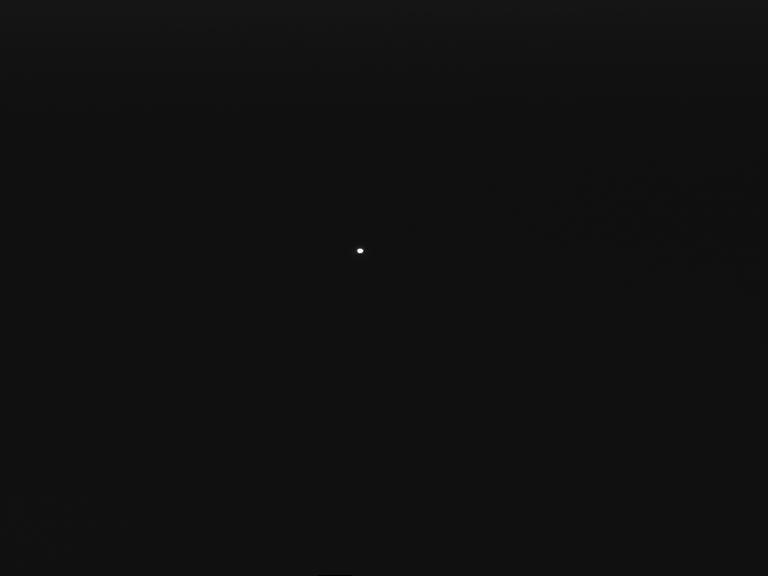

Supplement: S1 Dataset — This archive contains the captured data files used as the basis for the P4P solutions described in the manuscript. The data are provided in a directory hierarchy where each degree of freedom has a separate directory. And the calibration data is the captured data used in the camera calibration. (ZIP) [file pone.0134029.s001.zip › S1_Dataset/Pitch Angle/(28,0,0,0).tif]

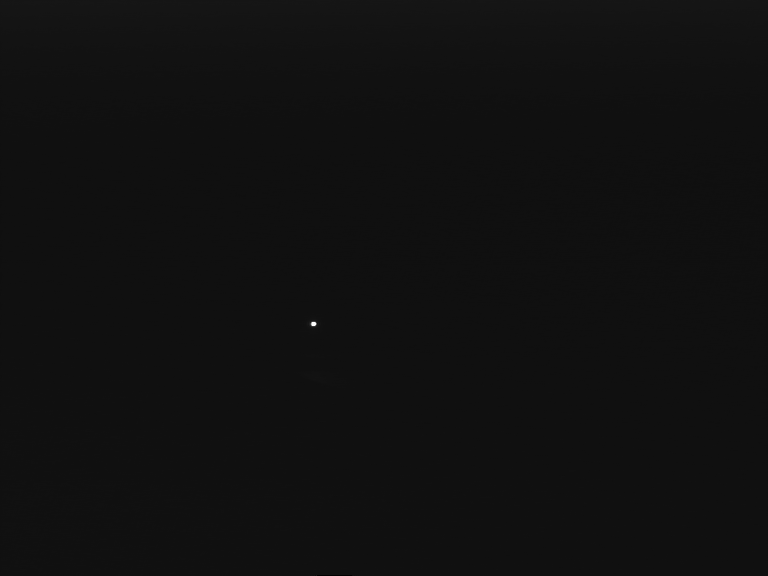

Supplement: S1 Dataset — This archive contains the captured data files used as the basis for the P4P solutions described in the manuscript. The data are provided in a directory hierarchy where each degree of freedom has a separate directory. And the calibration data is the captured data used in the camera calibration. (ZIP) [file pone.0134029.s001.zip › S1_Dataset/Pitch Angle/(28,0,0,1).tif]

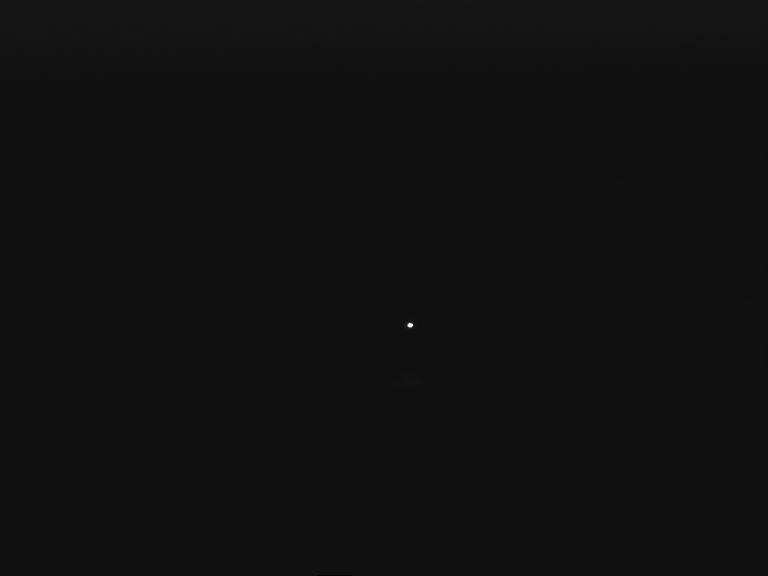

Supplement: S1 Dataset — This archive contains the captured data files used as the basis for the P4P solutions described in the manuscript. The data are provided in a directory hierarchy where each degree of freedom has a separate directory. And the calibration data is the captured data used in the camera calibration. (ZIP) [file pone.0134029.s001.zip › S1_Dataset/Pitch Angle/(28,0,0,2).tif]

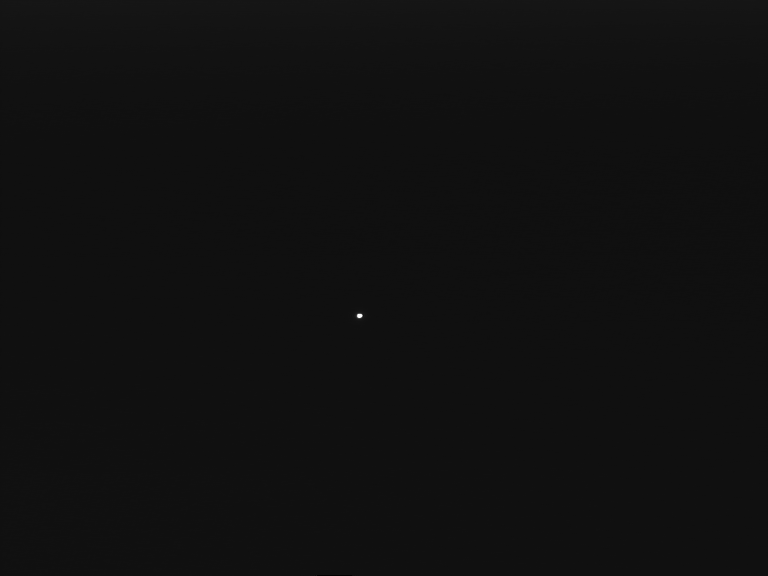

Supplement: S1 Dataset — This archive contains the captured data files used as the basis for the P4P solutions described in the manuscript. The data are provided in a directory hierarchy where each degree of freedom has a separate directory. And the calibration data is the captured data used in the camera calibration. (ZIP) [file pone.0134029.s001.zip › S1_Dataset/Pitch Angle/(28,0,0,3).tif]

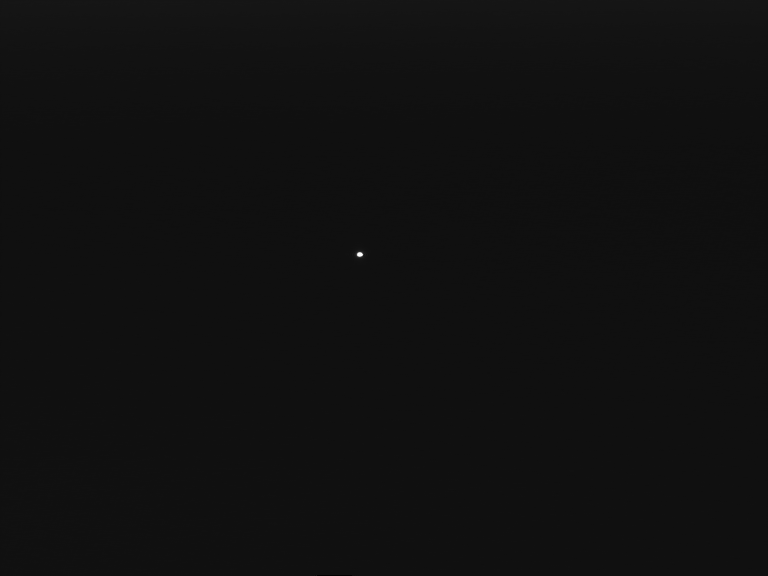

Supplement: S1 Dataset — This archive contains the captured data files used as the basis for the P4P solutions described in the manuscript. The data are provided in a directory hierarchy where each degree of freedom has a separate directory. And the calibration data is the captured data used in the camera calibration. (ZIP) [file pone.0134029.s001.zip › S1_Dataset/Pitch Angle/(29,0,0,0).tif]

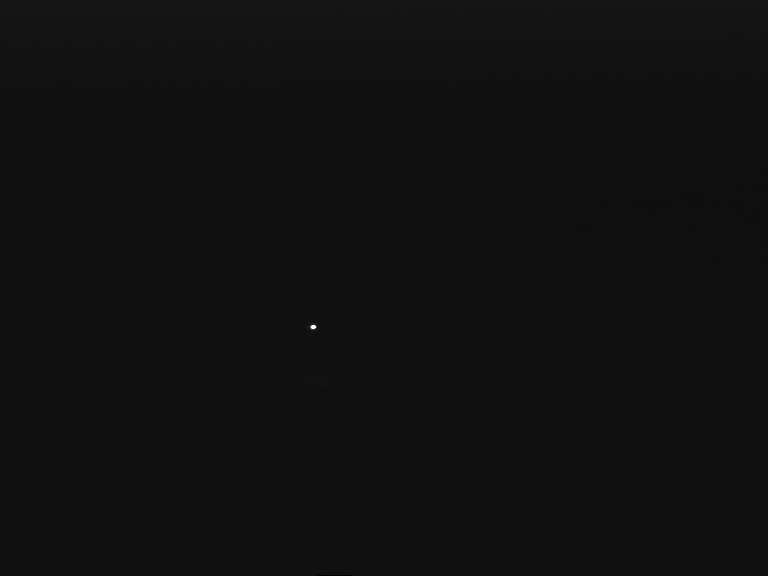

Supplement: S1 Dataset — This archive contains the captured data files used as the basis for the P4P solutions described in the manuscript. The data are provided in a directory hierarchy where each degree of freedom has a separate directory. And the calibration data is the captured data used in the camera calibration. (ZIP) [file pone.0134029.s001.zip › S1_Dataset/Pitch Angle/(29,0,0,1).tif]

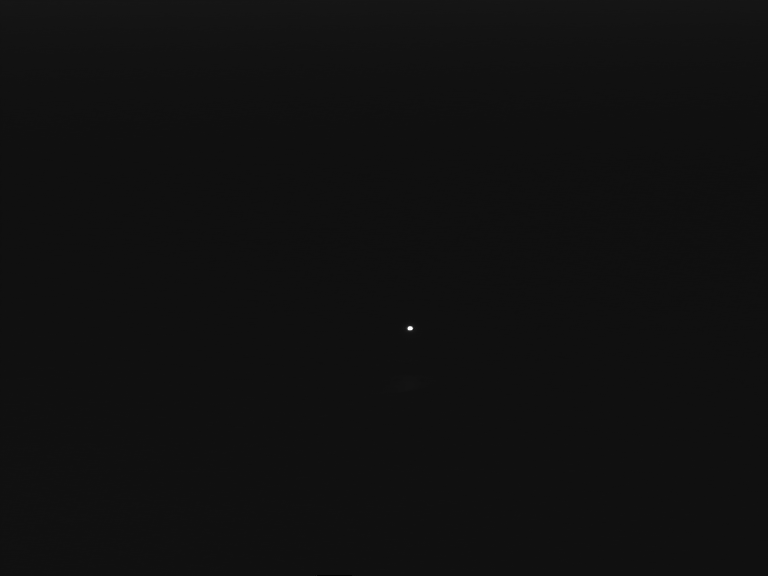

Supplement: S1 Dataset — This archive contains the captured data files used as the basis for the P4P solutions described in the manuscript. The data are provided in a directory hierarchy where each degree of freedom has a separate directory. And the calibration data is the captured data used in the camera calibration. (ZIP) [file pone.0134029.s001.zip › S1_Dataset/Pitch Angle/(29,0,0,2).tif]

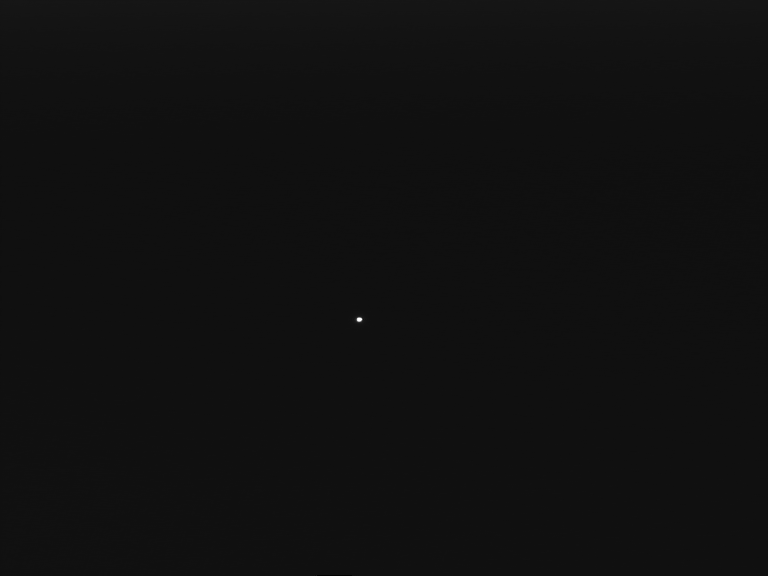

Supplement: S1 Dataset — This archive contains the captured data files used as the basis for the P4P solutions described in the manuscript. The data are provided in a directory hierarchy where each degree of freedom has a separate directory. And the calibration data is the captured data used in the camera calibration. (ZIP) [file pone.0134029.s001.zip › S1_Dataset/Pitch Angle/(29,0,0,3).tif]

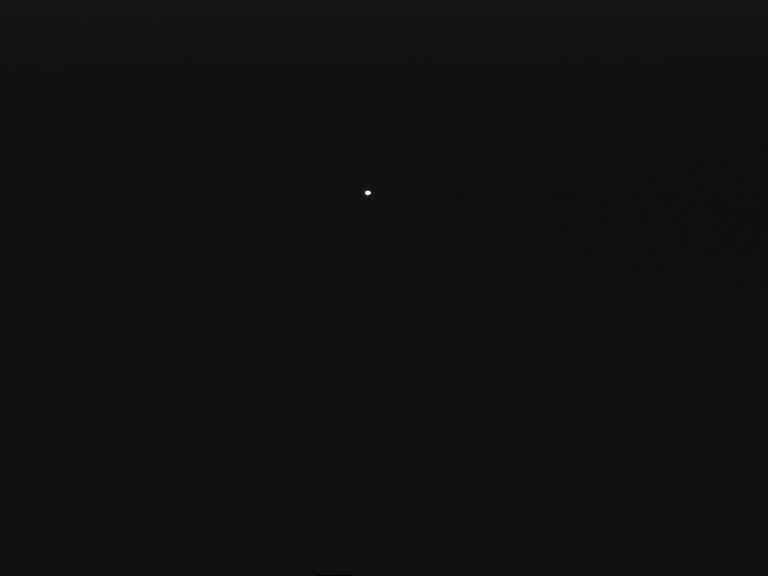

Supplement: S1 Dataset — This archive contains the captured data files used as the basis for the P4P solutions described in the manuscript. The data are provided in a directory hierarchy where each degree of freedom has a separate directory. And the calibration data is the captured data used in the camera calibration. (ZIP) [file pone.0134029.s001.zip › S1_Dataset/Pitch Angle/(3,0,0,0).tif]

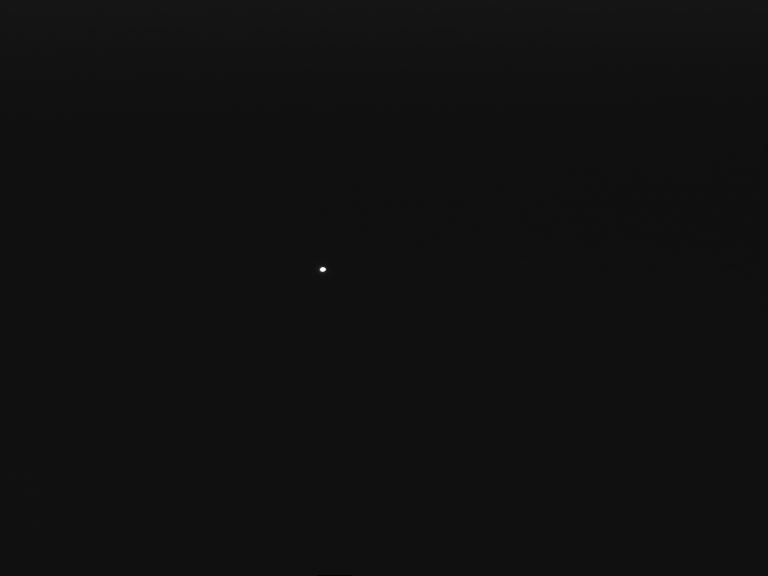

Supplement: S1 Dataset — This archive contains the captured data files used as the basis for the P4P solutions described in the manuscript. The data are provided in a directory hierarchy where each degree of freedom has a separate directory. And the calibration data is the captured data used in the camera calibration. (ZIP) [file pone.0134029.s001.zip › S1_Dataset/Pitch Angle/(3,0,0,1).tif]
